# Electron transfer from singlet fission dimers: possibilities and limitations

**DOI:** 10.1039/d6sc00259e

**Published:** 2026-05-11

**Authors:** Sree Chithra, Corentin Pigot, Claire Tonnelé, Ashley J. Redman, Sabine Richert, David Casanova, Victor Gray

**Affiliations:** a Department of Chemistry, Ångström Laboratory, Uppsala University Box 532 SE-751 20 Uppsala Sweden victor.gray@kemi.uu.se; b Donostia International Physics Center Donostia 20018 Euskadi Spain; c IKERBASQUE – Basque Foundation for Science Bilbao 48009 Euskadi Spain; d Institute of Physical Chemistry, University of Freiburg Albertstraße. 21 79104 Freiburg Germany; e Institute of Physical Chemistry II, Ulm University Albert-Einstein-Allee 47 89081 Ulm Germany

## Abstract

Photocatalytic production of H_2_ or hydrocarbons requires multiple electrons, and therefore the absorption of multiple photons and accumulation of photoexcited charges. Alternatively, multiexciton generation (MEG) could loosen this requirement. Despite extensive studies of singlet fission (SF), an MEG process, for solar cells, its use in photocatalysis has only recently been demonstrated. Herein, we present a detailed investigation of electron transfer (ET) from two tetracene based SF dimers, on time scales spanning the initial singlet excited state–triplet pair (TT) equilibrium and the longer-lived free triplet states. We find that the TT state behaves similarly to the singlet and thus can be used to increase the lifetime of singlet reactivity. We also show that in these covalently linked SF dimers, where TT recombines to form one free triplet, achieving more than 100% ET is challenging. Together with these findings and the input from theoretical calculations, we discuss the possibilities and limitations of ET from these dimers. By highlighting strategies to mitigate detrimental TT recombination pathways, these insights provide design principles for next-generation SF chromophores tailored for solar energy conversion and photoredox catalysis.

## Abstract 2

The singlet/triplet pair equilibrium in tetracene dimers can drive singlet‑like electron transfer but inevitably yields only one long-lived T_1_, limiting diffusion-controlled ET efficiencies from free triplets to <100% yield.

## Graphical abstract

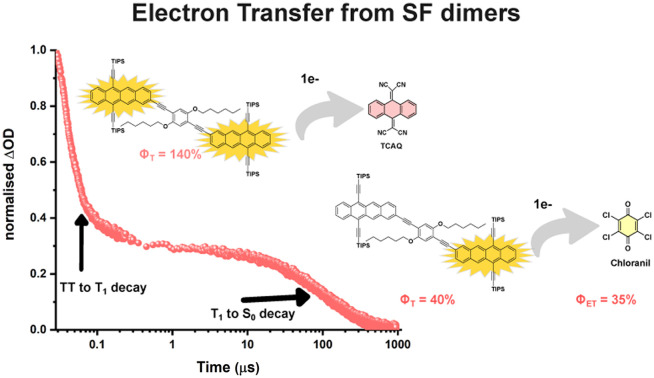

## Introduction

In 1912, Professor Giacomo Ciamician proposed that using sunlight to drive chemical reactions would be the best way to reduce humankind's reliance on fossil fuels.^1^ Over a century later, efforts towards this goal are as important as ever to mitigate the worst effects of climate change. A cornerstone in solar-to-fuel production is photocatalysis, which in turn often relies on photoinduced charge transfer (CT) events. However, for photocatalytic production of H_2_ or hydrocarbons, the chemical reactions require multiple electrons to proceed. Since photochemical processes, such as photoinduced CT, usually adhere to the Stark–Einstein photo equivalence law, *i.e.* for each photon only one primary photochemical event will occur^2^ multiple photons must be absorbed and the photoexcited charges (electrons and/or holes) accumulated to produce the final fuel. This challenge has similarities to thermalisation losses in solar cells, where regardless of the energy of the absorbed photon, only one electron–hole pair can be generated. One way of overcoming thermalisation losses in solar cells, which has gathered intense research interest, is multiexciton generation (MEG). MEG, where one photon results in the formation of multiple excited states, has been observed in both inorganic and organic materials.^3–8^ In organic materials MEG typically proceeds through singlet fission (SF), where an initially photoexcited singlet state is split into two triplet-excited states.^8–10^ The commonly accepted mechanism of SF is described as:^10^
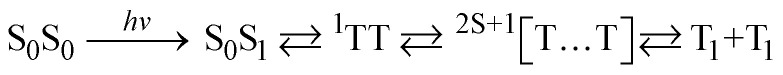
Here S_0_, S_1_, and T_1_, are the ground state singlet, first excited singlet and first excited triplet state, respectively. Upon photoexcitation of one chromophore, it can couple with a neighbouring molecule in the ground state to form a triplet pair state with overall singlet spin (^1^TT). This state can eventually lose electronic coupling to form a spin-correlated triplet pair ^1^[T…T], which upon loss of spin-coherence generates two triplet excited states (T_1_ + T_1_).^10^ Since these intermediate states are overall singlet in character, SF is spin-allowed and therefore can occur on ultrafast timescales, with triplet yields close to 200%.^9–12^ Hence, it is fundamentally different from intersystem crossing (ISC), which is spin-forbidden and proceeds with a maximum triplet yield of 100%. The weakly coupled triplet pair state can, however, interconvert between different spin-states (singlet, S = 0, triplet S = 1, and quintet S = 2). Since optical spectroscopy cannot directly distinguish between weakly and strongly bound triplet pair states, in the remaining text, we will refer to both types as the TT state.

Chromophores must meet certain criteria for the SF process to proceed. The primary energetic requirement is that the singlet excited state energy should exceed the combined energy of the two triplet excited states, according to the relation *E*_S_1__ ≥ 2*E*_T_1__. Another energetic requirement is that the energy level of higher lying triplet excited states should exceed 2 × *E*_T_1__, that is, *E*_T_*n*(*n*>1)__ ≥ 2*E*_T_1__ so as to avoid their formation by triplet–triplet annihilation (TTA).^9^ Together with these energetic considerations, the interacting chromophores must possess sufficient coupling to enable rapid and efficient singlet-to-triplet pair conversion, allowing it to compete effectively with other deactivation pathways such as fluorescence. Therefore, SF occurs most efficiently in solid state films, crystals, and in molecular dimers or oligomers in solution. However, the coupling should not be too large as then, the TT remains bound and deactivation will mostly occur by TTA. Hence a balanced interchromophoric coupling is desired to facilitate the dissociation of the TT to free triplet states.^11–14^ Another key factor for the dissociation of the TT is the possibility of triplet hopping, which enhances the entropic gain of the process. The feasibility for spatial separation of triplet excitons increases the efficiency of SF in solid-state systems.^13,15^

Even though SF has been studied intensely over the last decade for its use in solar cells, its use in photocatalysis has only recently been proposed and demonstrated in SF dimers.^16,17^ Covalently linked dimers offer strict control of distance and electronic coupling between two chromophores compared to crystal films and nanoparticles.^18–20^ Yet, the separation of the triplet pair into free triplets can be especially problematic in dimers. Conformational fluctuations are considered to drive the separation to free triplets from the intermediate TT state in some cases,^21,22^ however, competing pathways for the dissociation of the triplet pair have been identified:^23–25^^1^TT → S_0_S_0_ Singlet channel (loss of ≤100% of triplets)^3^TT → T_1_S_0_ Triplet channel (loss of ≤50% of triplets)^1^TT → T_1_S_0_ Intersystem crossing (loss of ≤50% of triplets)

Dimers, which allow for SF in solution, would be particularly relevant for photocatalysis. Hence, understanding how to control and use the triplet pair and free triplets for ET reactions is necessary to address fundamental questions such as what enables efficient multi-ET from SF dimers? Liu *et al.* highlighted that trimers and tetramers are more efficient than dimers for sequential two ET due to the increased separation between the formed charges.^26^ On the other hand, Nakamura *et al.* demonstrated photoinduced ET yields of 170–180% for a biphenyl-linked tetracene dimer with chloranil as the electron acceptor.^27^ Both of these examples established ET from the free triplets (T_1_ + T_1_), much less is known of what dictates ET from the triplet pair states and if it is possible to extract two electrons (holes) already from these states. In a pentacene-ironoxide cluster no ET was observed from the ^1^TT state, whereas it was observed for the free triplets, suggesting that the triplet pair and free triplets have distinct chemical properties.^28^ Interestingly, in 2018 Kim *et al.* suggested that concerted two ET from ^1^TT was possible from tetracyanomethylene quinoidal bithiophene derivatives.^29^ It has also been observed that charge separation from ^1^TT in solar cell blends is possible.^30^ Although the energy of the TT state is close to that of the singlet state, achieving photochemistry from this state would be valuable as the TT state can live longer as its recombination can be spin forbidden.^31^

One of the most studied acenes in SF, tetracene (Tc), is especially attractive for exploring ET processes relevant to photocatalysis. Fast SF in Tc-based chromophores yields triplet quantum yields close to 200%. Tetracene is also one of the few SF materials with a relatively high triplet energy of ∼1.3 eV, making it possible to study ET to a wider variety of electron acceptors.^32^

**Fig. 1:**
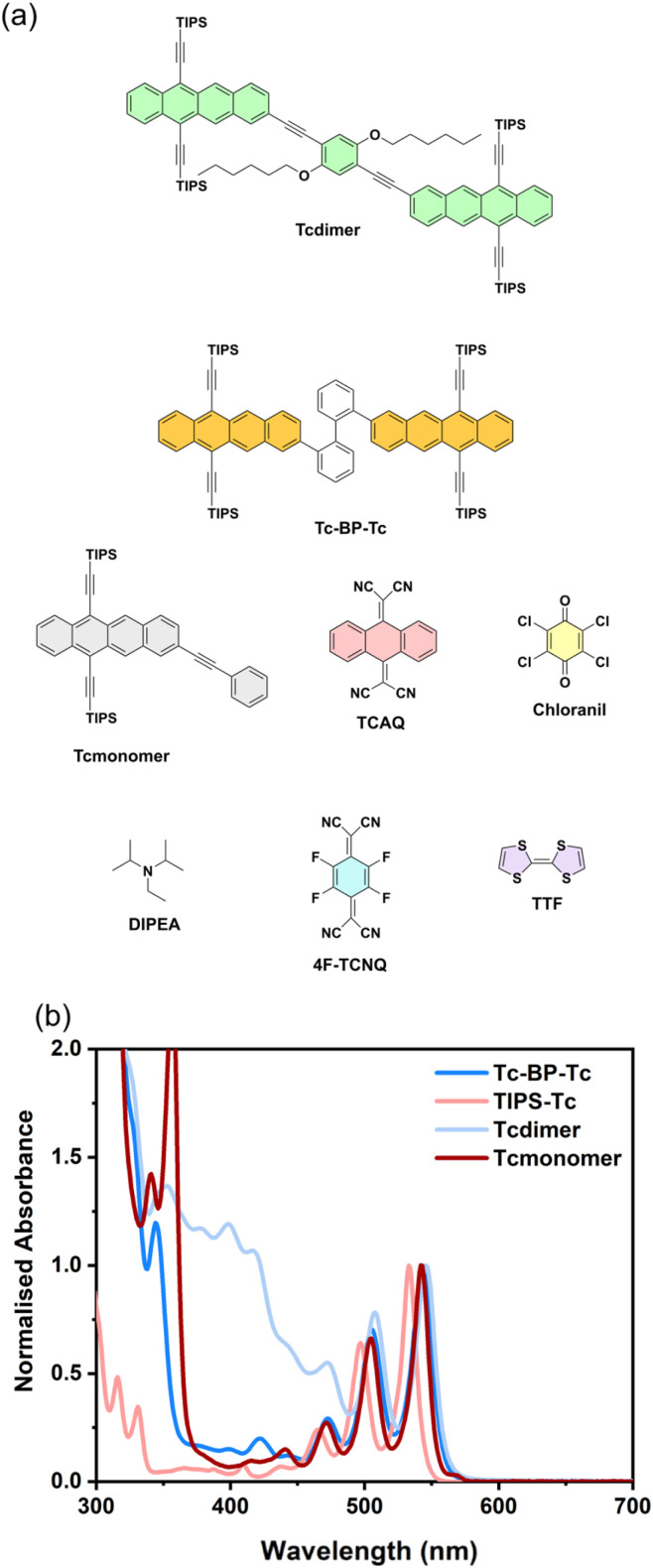
Structures of molecules (a) and steady state absorption of Tc based molecules (b) used in this study.

In this study, we clearly illustrate the difference in quenching between the TT/S_1_ equilibrium state and the free triplet state in two TIPS-Tc dimers, a newly synthesised Tcdimer and a previously reported^27^ Tc-BP-Tc, Fig. 1. This was done by using a range of electron and hole acceptors with varying redox potentials, enabling thermodynamically favourable CT quenching from either the TT or triplet states. Building on these findings, we discuss the possibilities and limitations of using such covalently linked SF systems for ET applications in photocatalysis and solar cells.

## Results and discussion

### Synthesis and design of the SF materials

Two dimers were synthesized for our study. As a reference Tc-BP-Tc was synthesized according to published procedures.^27^ To extend the scope of SF dimers we also designed a new Tcdimer. To enhance solubility, a phenyl linker with alkoxyhexyl side-groups was used. The linker L-3, composed of a phenyl unit modified with alkoxyhexyl groups was synthesized by nucleophilic substitution of phenolic groups (SI Section 2.1). After modification through standard Sonogashira coupling followed by deprotection with TBAF, linker L-3 was obtained with two acetylene functional groups, with an overall yield of 16% for the 3 steps (SI Section 2.1).

**Scheme 1:**
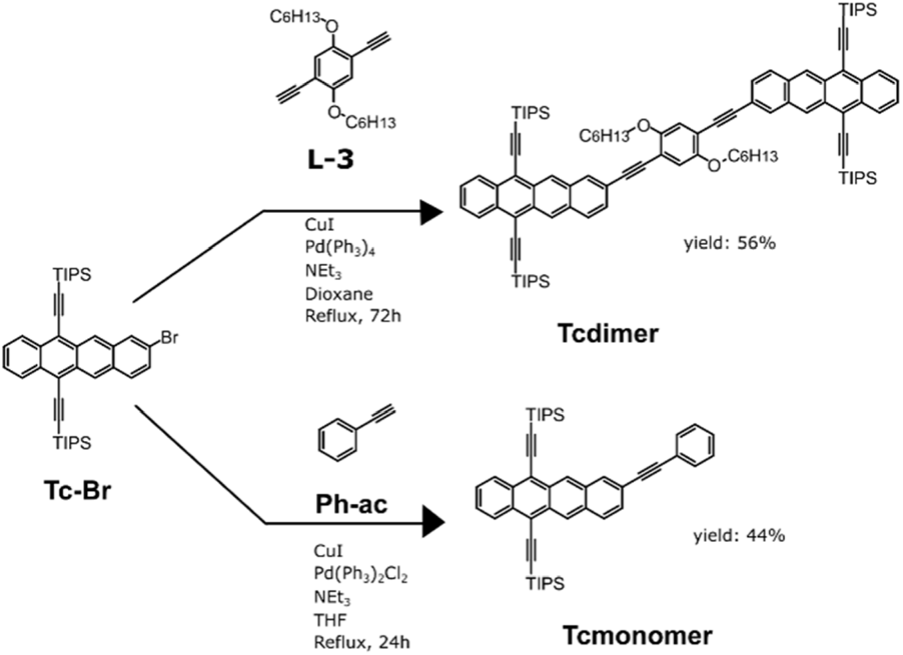
Synthetic routes for Tcdimer and Tcmonomer.

Subsequent coupling with two TIPS-Tc units through Sonogashira coupling conditions, afforded Tcdimer with 56% yield (Scheme 1). As a monomeric reference, Tcmonomer was synthesized in a similar manner using commercially available phenyl acetylene (Ph-ac) as the coupling partner (Scheme 1).

### Photophysical and electrochemical characterisation of Tcdimer

The steady state absorption spectrum of Tcdimer shows the characteristic vibronic peaks of tetracene between 450–550 nm (Fig. 1). These are slightly red shifted (∼5 nm) with respect to Tcmonomer. The strong absorption bands in the range 380–420 nm of the Tcdimer spectrum are absent in both the TIPS-Tc and the isolated linker (L-3) (Fig. S59), suggesting that the transitions responsible for these features arise from cooperative contributions of both components. Computational analysis of the electronic excitations in Tcdimer, in comparison with TIPS-Tc and the linker (Fig. S60–S62), reveals the presence of absorption bands that are unique to Tcdimer. These transitions are characterized by electronic excitations with partial CT character involving both TIPS-Tc moieties and the linker.

Whereas the fluorescence quantum yield of TIPS-Tc is around 80%,^33^ it drops to 23% in the case of Tcmonomer. This could be due to energy losses around the rotation of the phenylacetylene, although an increased rate of ISC was also observed for the Tcmonomer, revealed by the fast decay of the singlet state to form the triplet state within 6 ns (Fig. S28 and S30). Notably, the relative orientation of the two TIPS-Tc units in Tcdimer can give rise to two conformers, denoted syn and anti. Electronic structure calculations indicate that both are nearly isoenergetic (Fig. S63). Furthermore, the small computed energy barrier for their interconversion through rotation around the acetylene units of the linker (DE ≈ 1 kcal mol^−1^) suggests rapid dynamic interconversion. Furthermore, photophysical properties of the syn and anti-conformers are expected to be indistinguishable based on computed electronic excitation energies (Fig. S63). Nonetheless, the quantum yield of Tcdimer (16%) is lower than Tcmonomer. Time-resolved fluorescence (TCSPC) reveals monoexponential decays for TIPS-Tc and Tcmonomer, but a triexponential decay for Tcdimer (Fig. S20 and S21). The second and third decay components, much longer than the Tcmonomer lifetime, suggests that at least one additional long-lived state beyond the singlet would be involved. The steady state absorption, fluorescence and time-resolved fluorescence of Tcdimer in different solvents are given in (Fig. S20). The steady-state absorption spectra exhibit only minor solvent-dependent variations, with shifts of approximately 2 nm between THF and toluene, and about 4 nm between toluene and benzonitrile (PhCN). In contrast, the fluorescence spectrum in PhCN shows a modest red shift of roughly 10 nm relative to the other two solvents. Additionally, the second component of the fluorescence lifetime is noticeably shorter in PhCN, indicating faster nonradiative decay of the long-lived state in the more polar solvent environment.

#### Transient absorption and EPR of Tcdimer

**Fig. 2:**
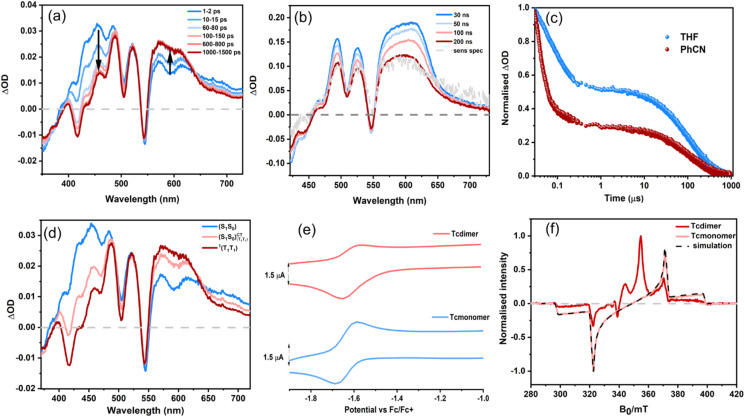
(a) fsTA spectra of Tcdimer (60 µM) in THF excited at 500 nm. (b) nsTA spectra of Tcdimer in THF (10 µM) excited at 545 nm. (c) The corresponding time profile of Tcdimer at 525 nm in THF and PhCN. (d) Evolution associated spectra (EAS) of the fs-TA spectra shown in (a). (e) Cyclic voltammogram of the reduction wave of Tcdimer (133 µM) in DCM in 0.1 M TBAPF_6_ with 100 mvs^−1^ scan rate (upper) and cyclic voltammogram of the reduction wave of Tcmonomer (133 µM) in DCM in 0.1 M TBAPF_6_ with 100 mvs^−1^ scan rate (lower) (f) Transient EPR measurements on Tcdimer and Tcmonomer, recorded at 80 K, the spectra are integrated over 0.4–0.6 µs after photoexcitation. The experimental spectra are scaled to the maximum absolute intensity. The data for the Tcmonomer are presented alongside a numerical simulation, see SI for simulation details.

To further probe the excited state dynamics of Tcdimer and to understand the long-lived state(s) involved, we now turn to transient absorption both on the femtosecond (fsTA) and nanosecond (nsTA) timescales. The fsTA spectra of Tcdimer in THF (Fig. 2a), upon exciting at 500 nm, show ESA between 400–750 nm, with overlapping ground state bleaching (GSB) signals at 425 nm, 500 nm and 545 nm, and stimulated emission (SE) at 600 nm. The red part of the spectra (550–630 nm) rises rapidly within 100 ps along with a corresponding decrease in the blue part (400–450 nm). A persistent GSB signal at 545 nm, together with two isosbestic points at 370 and 640 nm can be seen during the spectral evolution, suggesting a quantitative conversion between two species. A satisfactory fit is obtained using global analysis with a three-state consecutive model. The evolution-associated spectra thus acquired are provided in (Fig. 2d and S29). We assign the spectral evolution to the S_1_ state conversion to the TT state *via* a CT intermediate (S_1_S^CT^_0TT_), based on the similarities to a previously reported Tc dimer.^34^ The CT intermediate is rationalised by us and others, by the increased rate of formation in polar PhCN.^34^ This mechanism is also supported by the CT component in the initially excited S_1_ state, which has been shown to facilitate CT coupling with the triplet pair and rapid SF.^35,36^ We do not attribute the observed kinetics to different conformers, as our calculations of the major conformers (Section S14.2) show that their relevant electronic states are nearly isoenergetic. Despite the equilibrium between the TT and singlet states suggested by the repopulation observed in the PL data, we employ a three-state consecutive model for the fsTA analysis because the repopulation occurs on timescales significantly longer than those captured in the fsTA measurements.

Nevertheless, the data suggest that the S_1_ state converts to the TT state within 200 ps. In contrast, the fsTA spectra of Tcmonomer (Fig. S28) show a much slower decay of the singlet (∼6 ns).

Further evidence for SF in Tcdimer can be obtained from transient electron paramagnetic resonance spectroscopy (tr-EPR). This technique can detect distinct triplet sublevel populations and thereby distinguish between ISC and SF-born triplets. Particularly, it can detect the quintet state (S = 2), which is unique to SF. The tr-EPR spectrum of Tcmonomer and Tcdimer are compared in Fig. 2f. The Tcmonomer shows a broad triplet state spectrum with an eeeaaa polarisation pattern; the spectral width/splittings are determined by the zero-field (ZF) interaction matrix and the electron-spin polarisation arises from the transformation of the spin states developed during the intersystem crossing process to the high-field eigenstates, which is mediated by spin–orbit coupling interactions and is selective to the ZF spin sublevels.^37^ The tr-EPR spectrum of Tcdimer consists of outer and inner features, both of which are present at the earliest timescales accessible by the experiment. The inner features are assigned to a ^5^TT state, the field positions of the intense emissive and absorptive peaks are consistent with the |2, ±1〉 ↔ |2, 0〉 transitions for the *xy* orientations, assuming collinear ZF-interaction matrices of the two triplets in the TT state. The outer features are assigned to free triplet states, the resemblance of the electron-spin polarisation pattern across this region to the Tcmonomer spectrum, suggests the presence of ISC born triplets. These assignments are supported by pulse transient nutation experiments that clearly indicate the presence of both triplet and quintet states, see SI (Fig. S58). After the first ten microseconds following photoexcitation, the trEPR signal has largely decayed and, during this time, the overall spin-polarisation patterns for the triplet and quintet contributions are preserved. The exact mechanism responsible for the genesis of the spin polarisation observed in the quintet state is an open question. It was recently suggested that ^1^TT can undergo ISC to form T_1_, which could be a possibility in the current case.^25^. Simulations considering linear combinations of triplet states (formed either *via* ISC or from a high-field precursor) and quintet triplet pair states (with either high-field eigenstate or singlet-character population schemes and considering different coupling strengths) have, so far, been unable to adequately and uniquely model the entire spectrum for Tcdimer.

In the nsTA spectra of Tcdimer in THF (Fig. 2b), we see features similar to the last spectra seen in fsTA. These spectra also resemble the spectra of the sensitized triplet. Hence these features arise from a triplet-like species. The decay monitored at 525 nm (Fig. 2c) is clearly biphasic. An initial fast component, with a decay constant of 107 ns, is followed by a plateau at approximately 50% of the initial intensity. This intermediate state then decays more slowly, with a time constant of 240 µs. The longer decay is assigned to the decay of one free triplet. Support for this interpretation is provided by the decay of the triplet plateau being similar to the lifetime of an isolated triplet, as observed for the triplet decay of Tcmonomer (Fig. S33). In the case of a decoupled T_1_ + T_1_ state, the decay would proceed with a distinct, shorter lifetime due to TTA.^38^ Since the conformational fluctuations that would give rise to the decoupled triplet states are reversible, geminate TTA is expected to enhance the decay of the triplet features compared to the monomer without TTA, which is not observed in this case.

We also performed low temperature nsTA at 100 K in methyl-THF (MeTHF) to allow direct comparison with the EPR data, Fig. S54. Interestingly, the fast decay of the nsTA kinetics at 525 nm, occurs on the same time scale as the quintet triplet pair features from tr-EPR decay. Hence, we assign the first shorter decay seen in nsTA kinetics (Fig. 2c and S54) to that of the TT state. However, since the decay in tr-EPR arises not only from decay to the ground state, but also through loss of spin-polarization, we cannot rule out that the nsTA signal also includes other EPR-silent states.

To summarize, SF to form the TT state occurs over 200 ps. This is followed by triplet pair (TT) decay into only one free triplet over 100 ns. The remaining free triplet then decays monoexponentially over 240 µs. Similar TT decay into one free triplet has been reported for many moderately and strongly coupled dimers, with the proposed mechanism involving spin evolution of the ^2S+1^[T…T] state to ^3^TT, followed by internal conversion to T_1_.^38–42^ Another possible pathway is ^1^TT decay to T_1_*via* ISC, which was recently demonstrated by Ali *et al.*^25^ in anthradithiophene films. The kinetics in THF thus suggest a triplet yield less than 100% and we quantify the triplet yield using three independent methods (SI Section 8). First, from the fsTA spectra, the S_1_ and TT basis spectra are constructed and then normalised to the GSB they share. The resulting quantitative population dynamics suggest 100% formation of the TT from the S_1_ state (200% triplets). Based on the nsTA spectra, and assuming that the GSB contributions of the TT and T_1_ GSB scale with the number of monomers they occupy (*i.e*., 2 : 1), we quantify the population dynamics (see SI Section 8.2.1 for details). The analysis suggests close to 100% (95–145%) free triplet formation out of a maximum possible yield of 200%. A second method (SI Section 8.2.2) using ZnTPP as a reference at low excitation powers, gave rise to a free triplet yield of 72 ± 17% at 700 ns. Finally, combining the measured spot size with the triplet molar extinction coefficient gave a yield of 120–140% at 8 ns (SI Section 8.2.3). Considering that the TT state signal at 8 ns then decays to half its intensity over the next 100 ns, the actual free triplet yield would correspond to ∼70% if one assumes that the molar extinction coefficient of the TT state is roughly twice that of a free triplet. However, reports suggest that the extinction coefficient of the TT state is typically less than twice that of the free triplet, and depends strongly on the strength of the coupling in the pair state.^43,44^ Thus, the free triplet yield in this case would be slightly higher than 70%, consistent with the other estimates.

Together, these approaches consistently suggest a free triplet yield less than or close to 100%. These yield measurements were conducted in toluene; however, as both the dynamics and the final triplet intensity are nearly identical in THF (Tables S3, S4 and Fig. S29b and S32), we infer that the triplet yields are effectively the same in both solvents. However, in PhCN we see the triplet intensity becomes approximately 27% of the initial intensity (Fig. 2c). The triplet yield in this case was found to be 33 ± 8.2% at 700 ns using ZnTPP as a reference (SI Section 8.2.2).

#### Cyclic voltammetry of Tcdimer

To understand the redox properties and the potential for multi-electron redox activity we use cyclic voltammetry. As can be seen in the cyclic voltammograms in Fig. 2e and S23, there is no additional peak associated to multiple redox events of Tcdimer compared to Tcmonomer. We thus infer weak electronic coupling between the Tc units of Tcdimer, which is supported by calculations, *vide infra*. Importantly, the ratio of the integrated areas of the reduction waves of the Tc unit is approximately 1 : 2 for Tcmonomer : Tcdimer. This indicates that the Tc units in Tcdimer behave like two independent monomers, similar to what has been reported for Tc-BP-Tc.^27^ This also suggests that for these dimers, multi-redox events could be performed at the same potential, which could be beneficial for multi-ET reactions.

### ET studies using Tcdimer

#### ET from the TT state

Since the TT state of Tcdimer persists for up to 100 ns, we investigate whether it can take part in ET reactions or not. The acceptor 11,11,12,12-tetracyano-9,10-anthraquino-dimethane (TCAQ) can undergo concerted two-electron reduction,^45^ making it a suitable acceptor to investigate two ET from the TT state of SF dimers. In our case, however, TCAQ does not significantly quench the free T_1_ state significantly, (Fig. S40a). This is consistent with the positive value of Δ*G*_PET_ (Table 1). However, since SF in tetracene is isothermal, and if we assume that the TT state energy is close to that of the S_1_ state, driving force calculations suggest TCAQ can quench the TT state of Tcdimer.

**Table 1:** Estimated driving force for ET from/to the T_1_ and TT state of the Tcdimer to the quencher and if it is observed or not

Quencher	Δ*G*^a^ in eV (T_1_, substrate)	Δ*G* in eV (TT, substrate)	T_1_ quenching	TT quenching
4F-TCNQ	−0.67, 0.0	−1.67, −0.98	Yes	No
Chl	−0.12	−1.12	Yes	No
DIPEA	0.92	−0.08	No	Yes
TCAQ	0.48, 1.36	−0.52, 0.36	No	Yes
TTF	0.36, 0.66	−0.64, −0.34	No	Yes

a Driving force for the first ET, and where quenchers can accept/donate a second electron the driving force for this transfer is listed as a second vale.

**Fig. 3:**
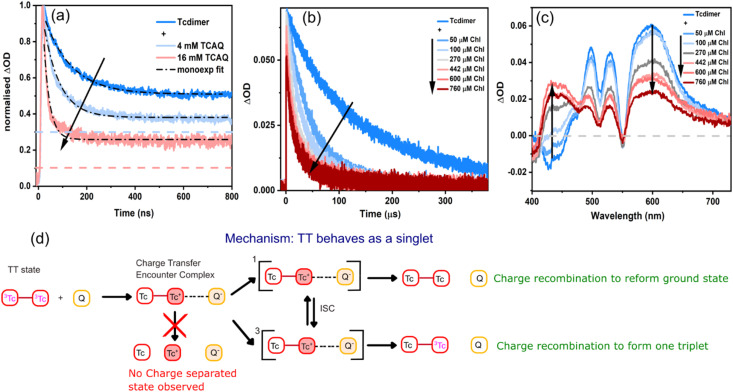
(a) Time profiles of Tcdimer (15 µM) at 525 nm with increasing concentrations of TCAQ in THF, with a collision rate of 1.45 × 10^9^ M^−1^ s^−1^ at 4 mM TCAQ. The collision rate is calculated as per the equation *k*_TT_ = *k*_TT_^0^ + *k*_c_[Q], with *k*_TT_ as the decay of the triplet pair in the presence of quencher, *k*_TT_^0^ represents the decay of the unquenched triplet pair, *k*_c_ represents collision rate, [Q] is the concentration of the quencher. The colored dashed lines show the expected triplet intensity based on the quenching efficiency. (b) The time profiles of Tcdimer (10 µM) at 600 nm with increasing concentrations of Chl in PhCN. The collision rate up to 100 µM was calculated to be 2 × 10^8^ M^−1^ s^−1^ (details provided in SI, Fig. S53). (c) The corresponding nsTA spectra of Tcdimer with increasing concentrations of Chl in PhCN at 10 µs. (d) Scheme showing a possible mechanism of oxidative quenching from the TT state with pathways for charge recombination and charge separation.

Indeed, quenching of the TT signal is observed in the nsTA experiments with increasing concentrations of TCAQ in THF (Fig. 3a). No spectral evolution is observed during quenching (Fig. S42b and c), suggesting no formation of long-lived charge separated species, with the remaining signal corresponding to the free triplet spectra.

Interestingly, the long-lived triplet signal that remains after TT quenching is significantly larger than what would be expected from the quenching efficiency. For example, with 4 mM TCAQ, the signal at 525 nm (corresponding to the TT and T_1_ ESA), shows a reduction in TT lifetime from 107 ns to 66 ns. The quenching efficiency (QE) can be calculated from the following equation:QE = 1 − *τ*/*τ*_0_where *τ*_0_ is the lifetime in the absence of any quencher (in this example, 107 ns) and *τ* is the lifetime in the presence of the quencher (in this example, 66 ns). This yields a QE of ∼40%, which means that ∼60% of the TT are unquenched. Upon quenching, the Tc–Tc^+^ species is formed, which quickly recombines to the ground state and does not contribute to the triplet signal. Therefore, the remaining signal arise solely from the unquenched TT population. In THF, where the TT signal is reduced to 50% of its initial value (Fig. 2c and 3a), the expected signal at 525 nm should be ∼30% of the initial intensity (*i.e.*, half of the ∼60% unquenched TT population, as indicated by the dashed lines in Fig. 3a). However, the experimentally observed signal in Fig. 3a is closer to 40% of the initial intensity. The same feature, a larger proportion of T_1_ signal than expected based on TT quenching, is observed with 16 mM TCAQ and during quenching with electron donors TTF and DIPEA. (Fig. S40 and Table S9). The calculations for the expected signal intensity are shown in Fig. S41).

Since the TT state forms within 200 ps, the quenching observed on the nanosecond timescale is attributed to the TT state rather than the S_1_. Yet, we cannot exclude quenching from S_1_ through the S_1_/TT equilibrium. Furthermore, since ET from/to T_1_ is energetically uphill (see SI Section 9), we rule out the possibility of single ET from the TT state leaving behind one unquenched triplet state. In other words, ET leading to triplet pair separation and oxidation of one tetracene unit is implausible in this case. On the other hand, CT from the TT state could result in complete quenching of the triplets, as shown in Fig. 3d, suggesting that the TT state behaves as a singlet state in this case.

Now we turn to discuss why the quenching with these (TCAQ, TTF and DIPEA) electron and hole acceptors in THF and toluene does not result in any observable long-lived charge-separated species (Fig. S42). Both one- and two-electron reduced TCAQ have absorption features within our detection window (400–700 nm),^46,47^ as such features are absent in our data, it indicates that charge-separated species are not formed efficiently. Together with this observation, we also see an unexpectedly high remaining triplet signal. To rationalize this behaviour, we propose the following explanation (Fig. 3d). Once the quencher diffuses to the TT excited Tcdimer, they first form a CT encounter complex. The CT encounter complex can be singlet or triplet in nature and since they lie close in energy, can interconvert through intersystem crossing. Since no charged species are observed, yet quenching is significant, the recombination within the CT encounter complex must be efficient before diffusional separation occurs. Such recombination from the singlet CT complex could yield the ground state species. Recombination from the triplet CT complex can result in CT-enhanced ISC to reform T_1_.^48–51^ Fast CT recombination would yield no or few long-lived charge-separated species and, if the formation of T_1_ is non-negligible, would explain the observed high triplet signal. In this case, quenching from TT resembles ET from a photoexcited singlet state with a maximum of 100% ET yield.

Thus, we also looked into the ET from Tcmonomer and TIPS-Tc to TCAQ and TTF (Fig. S43–S46). At high TCAQ concentrations (16 mM), the S_1_ states of Tcmonomer and TIPS-Tc are both quenched efficiently, yet no significant amount of charge-separated species was observed. With TCAQ and TIPS-Tc there was no increased triplet formation, indicating negligible reformation of T_1_ (see SI for details). Quenching of Tcmonomer with TCAQ also shows lower triplet yields, yet not to the extent of S_1_ quenching, suggesting some amount of CT enhanced ISC. Experiments with TTF also suggest possible charge recombination (CR) to form T_1_. Hence, we conclude that CR to form T_1_ is a plausible explanation for the increased T_1_ signal observed. Our results suggest that the TT state behaves similarly to the S_1_ state (*i.e.,* maximum 100% ET yield) when quenched by TCAQ, TTF, and DIPEA. Hence, SF dimers with relatively long-lived TT states can be used as a way of increasing the lifetime of singlet reactivity. It is challenging to unambiguously determine if ET proceeds from the TT or S_1_ state since they are in equilibrium. Consequently, ET from the S_1_ state would also lead to quenching of the TT state. Our results still suggest that the TT/S_1_ equilibrium effectively prolongs the lifetime of this reactivity which differs from free triplet reactivity.

#### ET from the T_1_ state

Next, we studied ET from the free triplets by using Chloranil (Chl) as the electron acceptor. In THF, quenching of the triplets was not observed (Fig. S47); hence, a more polar solvent, PhCN, was used to stabilize the charged products. Fig. 3b shows the triplet lifetime getting quenched with increasing concentrations of Chl. At higher concentrations (>100 µM), the triplet decay becomes biexponential. This might arise from some pre-associated populations or from some TT states that could also take part in the ET. If the latter were the case, then the second ET would be slower due to the already existing charge on the dimer.^26^ While this remains a possible explanation, we cannot confirm it. In any case, the overall ET yield determined below indicates that such double reduction is, if present, only minor.

In contrast to the TT state quenching reported above for TCAQ, in the case of Chl, we see clear spectral evolution (Fig. 3c). The decrease in triplet ESA is accompanied by the concomitant rise of the Chl radical anion at 450 nm. Using ZnTPP as a reference at low excitation powers, we determined the ET yield at 720 µM concentration of Chl to be 35 ± 7.1%, This agrees well with the free triplet yield of Tcdimer in PhCN (calculations given in SI, Fig. S38). We also see no visible quenching of the TT state at this concentration, (Fig. S48), supporting the claim that we are quenching only the free triplets in this case. Hence this corroborates the finding that the TT state gives rise to only one free triplet (see SI Section 11.1 for more details). In Tc-BP-Tc, the other covalently linked SF Tc dimer, the reported ET yield is (170 ± 10)%.^27^ We therefore closely look into its photophysics on the ns timescale and compare it with that of Tcdimer.

### Photophysical properties and ET behaviour of Tc-BP-Tc

#### Transient absorption and EPR

**Fig. 4:**
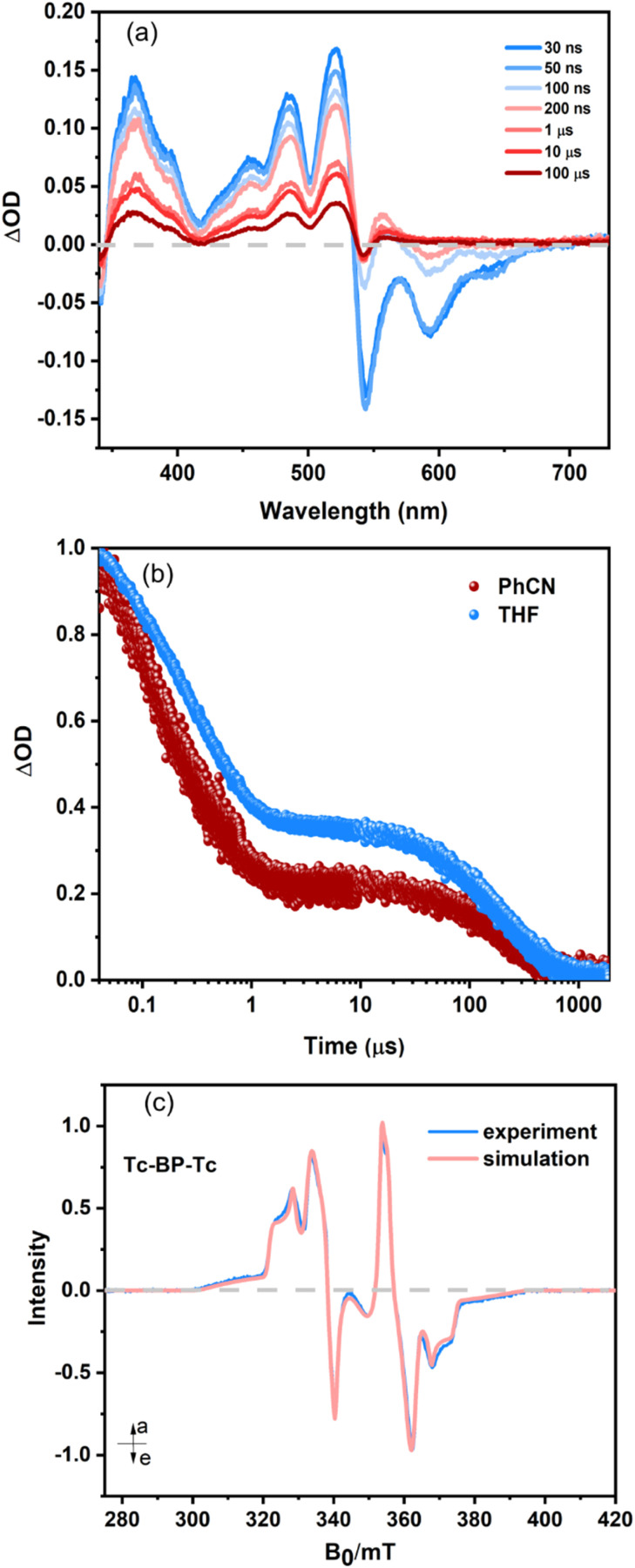
(a) nsTA spectra of Tc-BP-Tc (10 µM) in THF excited at 543 nm. (b) The corresponding time profile of Tc-BP-Tc at 520 nm in THF. Also shown in the Fig. are the time profiles at 525 nm in PhCN. (c) Transient EPR measurement on Tc-BP-Tc, recorded at 80 K, the spectrum is integrated over 0.4–0.5 µs after photoexcitation. For the simulation details, refer to SI.

Fig. 4a shows the nsTA spectra of Tc-BP-Tc in THF when excited at 543 nm. It is similar to the spectra reported in PhCN^27^ with the characteristic triplet ESA bands in the 500–520 nm range. On probing the triplet ESA band at 520 nm (Fig. 4b), we find that the signal intensity becomes ∼35% of the initial value within 400 ns and then persists for 240 µs. In PhCN, the signal at the plateau becomes 20% of its initial signal intensity and then decays with the same time constant of 240 µs. This also resembles the kinetic traces previously reported.^27^ From these observations, we conclude that the TT state of Tc-BP-Tc lives for up to 400 ns, almost 4 times longer than in Tcdimer, before recombining to form a single free T_1_. We note that the reported triplet yield of 180 ± 10% for Tc-BP-Tc was determined at early times (∼1 ns), which refers to triplets in the triplet pair.^27^ The greater loss of triplet signal, *i.e.*, a decrease to 35% triplet signal compared to 50% in Tcdimer in THF, could be because of multiple pathways of recombination occurring within the TT, *e.g.*, ^1^TT to S_0_S_0_ in addition to ^1^TT (*via*^5^TT to ^3^TT) to T_1_.^40,42^

tr-EPR measurements, performed at 80 K, confirm the existence of a ^5^TT state, Fig. 4c. Furthermore, modelling of the tr-EPR suggests strong coupling in the Tc-BP-Tc dimer, consistent with electronic structure calculations identifying stable conformers that exhibit Tc–Tc stacking (Fig. S68 and SI Section 14), possibly explaining the increased ^1^TT to S_0_ decay. The ^5^TT state is observed for several to tens of microseconds, and there is no evidence for the formation of spin-polarised free triplets, from either ISC or separation of the ^5^TT state. The characteristic spectral shape, which is reproduced by simulations, arises when the eigenstates of the coupled triplet pair are populated according to their singlet character. This results in a population of the ^5^TT state, where the populations of the magnetic sublevels display a complex dependence on the relative orientation of the molecule, and thereby its ZF interaction matrices, and the magnetic field, *B⃑*_0_. The presence of this population scheme, as far as we are aware, has only previously been unambiguously demonstrated in a limited number of intra-molecular SF materials and, although not fully understood, is proposed to arise in systems that display a fluctuating but large, relative to the ZF interaction, exchange interaction.^52–56^ Low temperature (100 K) nsTA for Tc-BP-Tc in MeTHF (Fig. S54) indicates that the initial decay component, assigned to triplet pair decay, is extended to 3.5 µs, compared to 400 ns at RT measurements. This time-scale is similar to the time scale of ^5^TT decay of Tc-BP-Tc in the tr-EPR spectra.

#### ET from Tc-BP-Tc

**Fig. 5:**
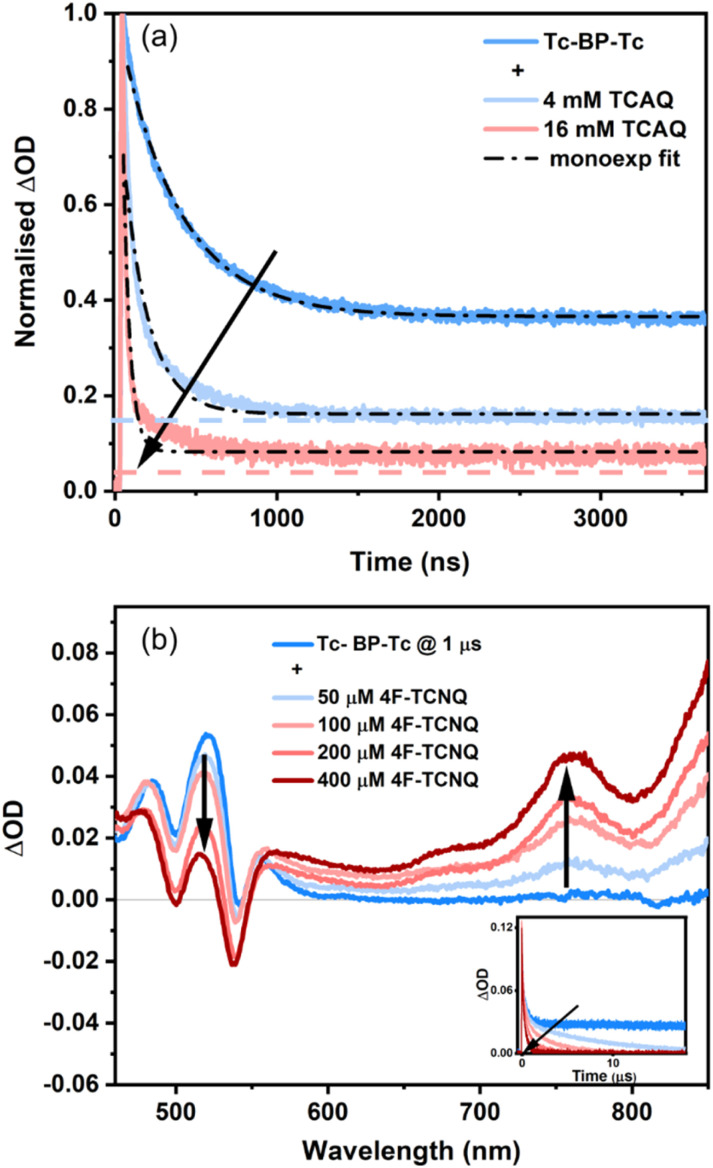
(a) Time profiles of Tc-BP-Tc (15 µM) at 520 nm with increasing concentrations of TCAQ in THF when excited at 543 nm. The collision rate at 4 mM TCAQ was found to be 9.61 × 10^8^ M^−1^ s^−1^. The coloured dashed lines show the expected triplet intensity based on the quenching efficiency. (b) nsTA spectra of Tc-BP-Tc (15 µM) in PhCN with increasing concentrations of 4F-TCNQ at 1 µs, when excited at 548 nm. The inset shows the corresponding time profiles at 520 nm. The collision rate in this case was found to be 6 × 10^9^ M^−1^ s^−1^ (calculations provided in SI, Fig. S53).

Quenching of the TT state of Tc-BP-Tc with TCAQ (Fig. 5a) resulted in no spectral evolution, yet a significant reduction of TT lifetime was observed, just as with Tcdimer. Here too the triplet signal is slightly greater than expected based on the TT quenching efficiency at 16 mM TCAQ, possibly due to CR to form T_1_, but the enhancement is not as pronounced as in Tcdimer (Table S14 and Fig. S52).

Next, we look at the quenching of free T_1_ using Chl in PhCN. As previously seen with the Tcdimer, we see spectral evolution with the decay in triplet ESA followed by the corresponding rise at 450 nm due to the Chl radical anion. However, unlike the case of Tcdimer (where triplet absorption is negligible at 450 nm, the wavelength used to monitor the differential absorption of the reduced chloranil anion), Tc-BP-Tc exhibits considerable overlapping absorption at this wavelength making it challenging to accurately determine the yield. Hence, we conducted quenching experiments with another electron acceptor, 2,3,5,6-tetrafluoro-7,7,8,8-tetracyanoquinodimethane (4F-TCNQ). Unlike the reduced Chl radical anion, whose absorption overlaps with that of the Tc-BP-Tc triplet ESA, the reduced 4F-TCNQ anion exhibits distinct features in the near infrared at 760 nm and 850 nm (Fig. 5b). Since the triplet ESA shows no signals in this region, the overlap is negligible, allowing for a cleaner and more reliable estimate of the triplet yield. Our results for a 400 µM concentration of 4F-TCNQ give an ET yield of 45 ± 10% (see SI, S11.2, for more details). At this concentration, all the triplets have been quenched and what remains is the TT decay (Fig. 5b, inset). The ET yield is in sound agreement with the observation that we see only about 20% of the initially formed triplets (180%)^27^ in PhCN (Fig. 4b). Hence, determining the ET yield at 100% T_1_ quenching is another approach of determining the free triplet yield in this dimer. Overall, these results are coherent with the observation that the TT state gives rise to only a single free triplet, which is then quenched on the microsecond timescale.

### Possibilities and limitations of SF dimers in multi-ET reactions

Our findings highlight a well-known limitation of covalently linked SF dimers for multielectron reactions; the correlated triplet pair (TT), confined to adjacent chromophores, has a high probability of recombination. This leads to reduced free-triplet yields, which undermines their potential for applications. A common strategy to address this limitation is the incorporation of additional repeating units. Increasing the oligomer length facilitates spatial separation of the triplet pair, enabling formation of the weakly correlated state (^1^T…T) that mediates free-triplet generation. Such behaviour has been demonstrated in trimers, tetramers, and hexamers, where the triplet yield rises markedly with increasing oligomer size.^38,57–59^

Another possibility is to directly link the electron acceptor with the SF dimer, which would enable intramolecular ET and override the diffusion limit. In this case, it might even be possible to extract two electrons directly from the TT state, which has also been looked into recently.^31^ The possibility for such a mechanism is suggested by experimental observations of triplet energy transfer directly from the triplet pair state.^42^ We investigated the possibility of such a stepwise two-ET through electronic structure calculations (See Section 14 in the SI for full details). Using the reported materials as an example, we find that both electron acceptors Chl and TCAQ preferentially interact with the tetracene backbone through π–π stacking interactions, which facilitate ET (Fig. S64 and S65). In the Tcdimer-TCAQ system, quenching of an individual triplet from the TT state is energetically unfavourable as the CT state lies significantly higher in energy (Table S17). In this case, the CT state and the triplet pair state are energetically close, consistent with single-ET leading to the formation of the Tc–Tc^+^–Q^−^ encounter complex, as described above in Fig. 3d. In contrast, in the Tcdimer-Chl and Tc-BP-Tc-Chl dyads, the CT state is nearly degenerate with the localized triplet state on one of the Tc moieties, possibly enabling the quenching of an individual triplet from the TT state forming ^1^Tc*–Tc^+^ + Q^−^ (See Section 14 in the SI for full details). Experimentally, however, we see no evidence for this in both Tc-BP-Tc and Tcdimer. With 400 µM of 4F-TCNQ and full quenching of the T_1_ signal, the fast decay that we assign to TT decay is not quenched (Fig. 5b, inset). This suggests that no ET occurs from the TT state to 4F-TCNQ. It is possible that the strong coupling of the TT state observed for Tc-BP-Tc hinders such ET. Interestingly, for the case of 4F-TCNQ we also see no evidence for ET from TT as if it behaved as a singlet state, forming Tc–Tc^+^–Q^−^. The driving force for this single ET is of the same order as that for single ET from the TT in Tcdimer to 4F-TCNQ, around −1.7 eV (Table 1), which likely lies in the Marcus-inverted region. Further studies to fully understand these observations are needed. Similar observations can also be seen in the case of Tcdimer, where the TT remains unquenched at 720 µM of Chl. (Fig. S48).

Another issue that must be understood for SF dimers to be applied to multi-ET reactions, is the fate of the remaining triplet after the first ET occurs. Hetzer *et al.* reported that in their pentacene dimer and tetramer, ET from one triplet quickly leads to loss of the remaining triplet through radical-enhanced ISC (RE-ISC), as they observe half of the expected ET yield.^60^ However, in their analysis they have not considered the 50% loss of signal when assigning evolution-associated spectra to TT and T_1_ + T_1_. A 50% loss in signal is similar to that observed by us and others^38–42^ (Fig. 2c and 4b), and indicates loss of one triplet, *i.e.*, TT → T_1_ + S_0_. If re-interpreted as such, the ET yield reported by Hetzer *et al.* would be consistent with efficient CT from the remaining T_1_. It is, thus, still unclear how problematic RE-ISC might be in SF-based ET schemes.

## Conclusions

Herein, we have, in detail, studied ET from two Tc-based SF dimers on timescales spanning both the singlet state-TT equilibrium and triplet states. This was performed by employing electron and hole acceptors with varying driving forces for ET to or from the TT and triplet states. Our results show that the TT state behaves analogously to a singlet when the quencher it encounters has sufficient driving force to quench the singlet state, enabling electron-transfer reactions that benefit from the prolonged reactivity associated with the TT state. Moreover, we demonstrate that because the TT state recombines to yield a single T_1_ in these dimers, achieving an electron-transfer yield exceeding 100% in diffusion-limited reactions is inherently challenging. Drawing on these conclusions, the possibilities and limitations of multi-ET reactions using these systems have been discussed. These are key considerations needed to design next-generation SF chromophores for applications in solar cells and photoredox catalysis.

## Author contributions

S. C. carried out the steady-state and transient spectroscopic measurements (fsTA and nsTA), performed data analysis, contributed to the mechanistic interpretation and writing of the manuscript. C. P. carried out the syntheses of the molecules involved, planning of the research and contributed to the writing and reviewing of the manuscript. A. J. R. carried out the tr-EPR measurements and the corresponding data analysis and writing of the manuscript. C. T. carried out the theoretical calculations. C. T. and D. C. designed the computational study, analysed the results, and contributed to the writing and reviewing of the manuscript. S. R. guided the tr-EPR measurements and contributed to the reviewing of the manuscript. V. G. conceived the research idea, devised both the synthetic and spectroscopic studies, guided the mechanistic interpretation and contributed to the writing of the manuscript.

## Conflicts of interest

There are no conflicts to declare.

## Supplementary Material

SC-OLF-D6SC00259E-s001

## Data Availability

Data underlying the figures and conclusions in this publication is available at Zenodo data repository at: https://doi.org/10.5281/zenodo.13869022. Supplementary information (SI): additional experimental details, data and details for the synthesis, additional spectroscopic data (TCSPC, transient absorption), details on triplet and electron yield calculations, and additional computational details and results. See DOI: https://doi.org/10.1039/d6sc00259e.
