# Supplementary material for: Electron transfer from singlet fission dimers: possibilities and limitations
Source: Chem Sci. 2026 May 11;17(26):12970–80. doi: 10.1039/d6sc00259e (PMC13195404; doi:10.1039/d6sc00259e)
Supplement: SC-017-D6SC00259E-s001 [file SC-017-D6SC00259E-s001.pdf]

Supplementary Information (SI) for Chemical Science.
This journal is © The Royal Society of Chemistry 2026

Electronic Supplementary Information for: Electron transfer from singlet fission dimers: possibilities and limitations†

Sree Chithra‡[a], Corentin Pigot‡[a], Claire Tonnelé[b,c], Ashley J. Redman[d,e], Sabine Richert[d,e], David Casanova[b,c] and Victor Gray*[a]

[a]Department of Chemistry, Ångström Laboratory, Uppsala University, Box 532, SE-751 20 Uppsala, Sweden

[b] Donostia International Physics Center, 20018 Donostia, Euskadi, Spain

[c] IKERBASQUE – Basque Foundation for Science, 48009 Bilbao, Euskadi, Spain

[d]Institute of Physical Chemistry, University of Freiburg, Albertstraße. 21, 79104 Freiburg, Germany

[e]Institute of Physical Chemistry II, Ulm University, Albert-Einstein-Allee 47, 89081 Ulm, Germany

Table of Contents

1. GENERAL INFORMATION 3

1.1 Spectroscopic measurements 3
1.1.1 Nanosecond transient absorption (nsTA) measurements 3
1.1.2 Femtosecond transient absorption (fsTA) measurements 3
1.1.3 Transient electron paramagnetic resonance measurements (tr-EPR) and pulsed EPR 3

1.2 Cyclic voltammetry measurements 5

1.3 UV-visible spectroelectrochemistry measurements 5

2. SYNTHESIS 5

2.1 Synthesis of Tcdimer: 5
2.1.1 Synthesis of L-1:[7] 5
2.1.2 Synthesis L-2 7
2.1.3 Synthesis L-3: 8
2.1.4 Synthesis Tcdimer: 10
2.1.5 Synthesis Tcmonomer: 13

2.2 Synthesis of Tc-BP-Tc: 15
2.2.1 Synthesis of Tc-boronicacid pinacole ester (Tc-Bpin): 15
2.2.2 Synthesis of Tc-BP-Tc: 16

3. UV-VIS ABSORPTION AND FLUORESCENCE 20

4. ELECTROCHEMISTRY 21

5. FSTA DATA OF TCDIMER AND TCMONOMER 22
6. GLOBAL ANALYSIS OF FSTA 23
24
7. NSTA OF TCDIMER 25
8. DETERMINATION OF THE TRIPLET YIELD OF TCDIMER 26
8.1 Determination of the triplet extinction coefficient: 26
8.2 Determination of the triplet yield in Tcdimer: 28
8.2.1 Method I: 28
8.2.2 Method II: 29
8.2.3 Method III: 31
9. CALCULATIONS OF DRIVING FORCE FOR ELECTRON TRANSFER 32
9.1 Oxidative quenching: 32
9.2 Reductive quenching: 33
10. ADDITIONAL QUENCHING EXPERIMENTS 34
11. DETERMINATION OF THE ELECTRON TRANSFER YIELD: 39
11.1 ET yield from Tcdimer to Chl 39
11.2 ET yield from Tc-BP-Tc to 4F-TCNQ 40
11.3 Collision rate calculations: 42
12. LOW TEMPERATURE NSTA EXPERIMENTS: 43
13 ADDITIONAL TR-EPR DATA: 44
14. COMPUTATIONAL DETAILS 48
14.1 Absorption of Tcdimer, TIPS-Tc and linker 48
14.2 Tcdimer anti/syn conformers 51
14.3 Photophysical properties of anticonformers of Tcdimer with chloranil and TCAQ 52
14.4 Photophysical properties of *syn* conformers with chloranil 55
14.5 Photophysical properties of *Tc-BP-Tc and Tc-BP-Tc-Chl* 57
14.6 Modelling the second electron transfer in Tcdimer-Chl and Tc-BP-Tc-Chl 63
15 REFERENCES 63

1. General Information

All reagents and solvents were purchased from Aldrich, Fluorochem or TCI Europe and used as received without further purification. ^{1}H and ^{13}C NMR spectra were determined at room temperature in 5 mm o.d. tubes on a Bruker Avance 400 spectrometer of the Spectropole: ^{1}H (400 MHz) and ^{13}C (100 MHz). The ^{1}H chemical shifts were referenced to the solvent peak $CDCl_3$ (7.26 ppm) and the ^{13}C chemical shifts were referenced to the solvent peak $CDCl_3$ (77 ppm). **Tc-BP-Tc** dimer was synthesized as previously reported in the literature,[1] without modification and in similar yields.

1.1 Spectroscopic measurements

Unless specified otherwise, the samples were prepared inside an argon glovebox with oxygen levels below 10 ppm. The solvents were obtained from a Solvent Purification System (SPS) from Inert and then freeze pump thawed before bringing them inside the glovebox. The steady state UV-visible absorption was performed using Agilent Cary 5000 and the steady-state fluorescence spectra were obtained using Fluorolog 3-222 emission spectrophotometer (Horiba Jobin-Yvon). Spectra were acquired with FluorEssence software using a right-angle detector geometry (90° angle) along with corrections for variations in the lamp output and detector response. Fluorescence quantum yields were measured using Rhodamine 6G in ethanol $\phi_F = 0.95$ as the reference.[2] Time-resolved fluorescence measurements were carried out using FS5 system (Edinburgh instruments). The samples were excited using the picosecond pulsed light-emitting diode (EPLED) at 470 nm. The measurement range was 200 ns with the peak preset at 10^4 counts in 1024 channels. The IRF was recorded using dilute LUDOX solution in de-ionized water at 470 nm excitation.

1.1.1 Nanosecond transient absorption (nsTA) measurements

The nsTA measurements were carried out using a Nd:YAG laser (Ekspla, NT342B laser) as the source of excitation of the samples with an OPO set at 545 nm having energies of 7-9 mJ/pulse. The spectrometer (LP920, Edinburgh Instruments) comprises of a pulsed 450 W ozone-free Xe arc lamp, a symmetrical Czerny-Turner monochromator (TMS300) with 5 nm bandwidth and detectors for both single kinetic traces (LP900 photomultiplier, with Tektronix TDS3012C oscilloscope) and entire spectra (Andor SH720 ICCD camera). The kinetic traces were fit using Origin.

1.1.2 Femtosecond transient absorption (fsTA) measurements

The fsTA measurements were carried out using a Ti:sapphire-based amplifier along with an integrated oscillator and pump lasers (Coherent Libra). A beam splitter splits the laser fundamental (800 nm, 3 kHz, fwhm 40fs) into a pump and probe which were then directed towards the UV–vis–NIR TA spectrometer (TAS, Newport Corp.). Optical parametric amplifiers (TOPAS NirUVis, Light Conversion) were used to generate the excitation wavelength (pump-500 nm). The pump was passed through a waveplate that was set at magic angle and attenuated using a neutral density filter before reaching the sample. A calcium fluoride crystal was used to generate the probe supercontinuum (UV–vis) and an optical delay ($t_{window} \leq 8$ ns) was used to vary the path, thereby recording the transient spectra at varying pump–probe delay times on a silicon diode array (Newport custom-made). The samples were kept in 1 mm quartz cuvettes and had an absorbance of 0.2-0.3 at 500 nm. A pump power of 1mW was used for the measurements and focused on the sample in an approximately 0.05 mm^2 spot. The TA datasets were background and chip corrected using SurfaceXplorer software.

1.1.3 Transient electron paramagnetic resonance measurements (tr-EPR) and pulsed EPR

Sample Preparation: For the EPR measurements the tetracene compounds were prepared as toluene solutions, having a UV-Vis absorbance of 0.2-0.3 in a 2 mm cuvette. The solutions were loaded into 3.8 mm outer-diameter and 3 mm inner-diameter clear-fused quartz tubes (QSIL Ilmasil PN). The samples were rapidly frozen in liquid nitrogen prior to spectrometer insertion—a glassy solid was confirmed by visual inspection.

Tr-EPR: LASER excitation at 544–548 nm, as indicated, was provided by an Ekspla NT230 series tuneable diode-pumped LASER system at a repetition of 50 Hz (pulse duration ≈5 ns). Excitation energies were ≈1 mJ/pulse incident on the optical window of the cryostat. After the last turning mirror, the light was depolarised using an achromatic depolariser. A Stanford Research Systems digital delay generator (DG645) was used for synchronisation of the LASER system and EPR spectrometer (SpecJet TRIG IN). Transient EPR experiments were performed at the X-band on a Bruker ELEXSYS E580 spectrometer equipped with a critically coupled Bruker ER-4118X-MD5-W1 dielectric resonator. The experiments were performed at 80 K, using liquid nitrogen in combination with a continuous-flow cryostat (CF935, Oxford Instruments) and temperature controller (ITC4, Oxford Instruments). The data were acquired by direct-detection with the transient recorder (SpecJet-II digitiser) without lock-in amplification using a microwave power of 0.09–1.5 mW (corresponding to a attenuator settings of 32–20 dB). The data were acquired in cw mode using the diode standard pre-amplifier output with acAFC; the signal was

amplified using a Stanford Research Systems low-noise voltage preamplifier (SR560) in a 3 kHz–1 MHz bandpass prior to entering the SpecJet Ch2 input. To minimise complicated background signals/artefacts, the trEPR experiments were acquired over several repeats/field sweeps, typically limiting the acquisition of each sweep to 15–20 min. The dataset repeats were acquired using the Python XeprAPI, saving the sweeps individually. The data were processed using lab-written Python routines. During dataset/repeat aggregation, the data were compensated for minor drifts in the mw frequency and interpolated along the field abscissa. The DC offset and LASER backgrounds were removed by two successive 1D baseline-corrections based in the pre-LASER time points as well as the low- and high-field off-resonance transients. The time abscissa was shifted to account for the LASER pulse position and the field abscissa was frequency-corrected to 9.75 GHz and calibrated with a standard carbon fibre sample, with a known g-factor (g = 2.002 644).[3]

Transient EPR simulations: These were performed in MATLAB using functions from the EasySpin package.[4,5] The spectra were simulated using the pepper function. The **Tcmonomer** system was considered as a triplet state formed via spin–orbit coupling mediated intersystem crossing (spin_system.initState = {[...],'xyz'}). The **Tc– BP–Tc** dimer system was considered as a pair of coupled triplets, for simplicity the spin Hamiltonian parameters of the individual triplets were taken from the **Tcmonomer** best fit parameters. It was found that, in general, simulations with a ferromagnetic coupling ($J > 0$, for $\widehat{H}_{ex} = +J\widehat{S_A^T}\widehat{S_B^T}$ provided more faithful reproductions of the experimental spectrum; furthermore, it was found that the spectral changes observed for exchange couplings larger than approximately 120 GHz were modest. The triplet pair states were populated by their singlet character using the short-hand notation spin_system.initState = 'singlet'. For the **Tc–BP–Tc** simulations all interaction matrices were assumed to be collinear.

Table S1: *Parameters for the tr-EPR simulations presented in the main text.*

	**Tcmonomer**	**Tc-BP-Tc**
g_{iso}	2.0028	2.0028
D_T, E_T / MHz	[1412, −18.0]	[1412, −18.0]
dip_{TT} / MHz	-	[39.1, −45.6]
J_{TT} / GHz	-	263
initState	[0.468, 0.532, 0], 'xyz'	'singlet'
HStrain / MHz	[37.6, 22.5, 38.4]	[40.1, 27.2, 60.6]

Pulse EPR: Pulse EPR experiments were performed on a Bruker ELEXSYS E580 X-/Q-band spectrometer. Measurements were performed at the Q-band with a Bruker EN 5107-D2 resonator and a 50 W solid-state amplifier (Bruker) at a temperature of 80 K using liquid nitrogen with a continuous-flow cryostat and (Oxford Instruments CF935) and a temperature control system (Oxford Instruments ITC 4). The extent of resonator over-coupling was chosen to maximise sensitivity, balancing the excitation bandwidth and sufficiently minimising the ringing observed for the chosen τ-value. For pulse EPR measurements at the Q-band, the sample was excited through the top of the sample holder with depolarised light at 548 nm using an optical fibre with a diameter of 0.8 mm. The excitation energy was ≈0.5 mJ/pulse, measured at the fibre output, at a repetition rate of 50 Hz (pulse duration ≈5 ns).

An echo-detected field sweep was recorded using a two-pulse primary echo sequence with pulse lengths of $t_{\pi/2}$ = 12 ns and t_π = 24 ns, an inter-pulse delay, τ, of 180 ns, and an echo integration gate of 80 ns. A two-step phase cycle, [+(+x) − (−x)], was applied on the first pulse ((x)x). The mw pulse sequence was positioned 700 ns after the LASER pulse.

Field-dependent transient nutation measurements used the sequence hv − DAF − β − τ − t_{24} ns − τ − echo. During the experiment the flip angle of the first pulse (β) was varied by increasing the pulse length in steps of 4 ns, starting at 12 ns. The mw pulse amplitudes were optimised to maximise the p_{12} ns − τ − p_{24} ns echo at circa 1208 mT, one of the field positions that displayed the highest nutation frequency. A four-step phase cycle x[x] with linear combination coefficients +1, −1, +1, −1, i.e. [·+(+x) −(+y) +(−x) −(−y)] was used to remove unwanted signals, including possible contributions from the (−, −) pathway. Echo integration used an 80 ns gate, wide relative to the mw excitation, in an attempt to reduce off-resonance effects. The data were baseline corrected using a polynomial background function, apodised with a Hamming window, zero-filled and the cross-term averaged FFT was calculated, with the absolute-value spectra presented. The frequency abssica was normalised by division with a reference frequency obtained for a spin-1/2.

1.2 Cyclic voltammetry measurements

Cyclic coltammetry (CV) was performed using a 3 mm glassy carbon as the working electrode, a platinum wire counter electrode, Ag wire as the reference electrode and an AUTOLAB potentiostat (PGSTAT302). It was corrrected for junction potentials by using the ferrocene couple (Fc^+/Fc) as the internal reference. A solution of 0.1 M $TBAPF_6$ in THF containing the sample was purged with N_2 for 15 min after which the voltammograms were recorded.

1.3 UV-visible spectroelectrochemistry measurements

These were performed in a diode array spectrophotometer (Agilent 8453) using a quartz cell with a pathelength of 1 cm having pencil lead electrodes with reticulated vitreous carbon foam as the working and counter electrodes along with the Ag wire mentioned above as the reference electrode inside the glovebox. An AUTOLAB potentiostat (PGSTAT302) was used to perform controlled potential electrolysis of the sample and the time-resolved spectra was recorded.

2. Synthesis

2.1 Synthesis of Tcdimer:

The phenylacetylene linker **L-3** was synthesised in 3 steps from 2,5-dibromobenzene-1,4-diol. Brominated TIPS-tetracene **Tc-Br** was synthetized as described in the literature[6] and coupling to the linker to form **Tcdimer** was done through Sonogashira coupling conditions.

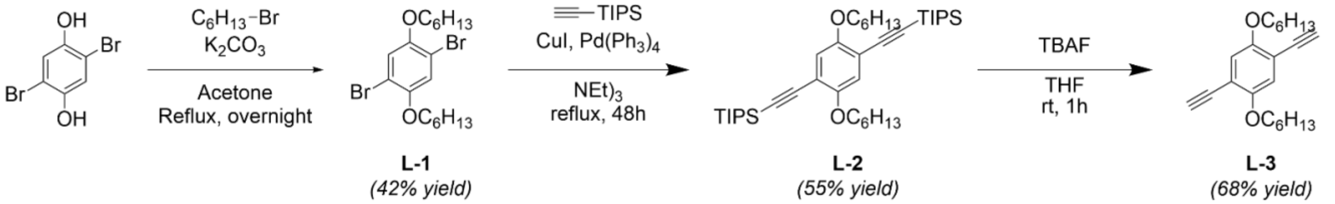

2.1.1 Synthesis of L-1:[7]

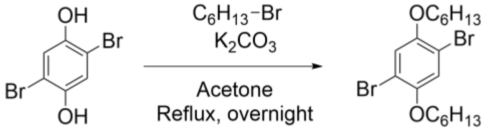

In a 250 mL RBF, 2,5-dibromo-1,4-hydroquinone (5.36 g, 20 mmol, 1 eq.) was added followed by 100ml of acetone. K_2CO_3 (13.8 g, 0.1 mol, 5 eq.) was added to this suspension and stirred for 10 min. Then 13.8 mL (98 mmol, 5 eq.) of bromohexane were added and the mixture was refluxed overnight. Evaporation of the volatiles afforded a solid which was dissolved in ethyl acetate and washed twice with water then with brine. Filtration on silica followed by evaporation afforded a brown solid. It was then purified by column chromatography (silica gel, pentane/DCM, 5/1) yielding a white powder (3,72 g, 42% yield).

^{1}H NMR (400 MHz, Chloroform-D) δ 7.08 (s, 2H), 3.94 (t, J = 6.5 Hz, 4H), 1.85 – 1.76 (m, 4H), 1.53 – 1.44 (m, 4H), 1.34 (dq, J = 7.2, 3.6 Hz, 9H), 0.93 – 0.89 (m, 6H) ppm.

^{13}C NMR (101 MHz, Chloroform-D) δ: 150.20, 118.57, 111.24, 70.41, 31.61, 29.21, 25.73, 22.70, 14.15 ppm.

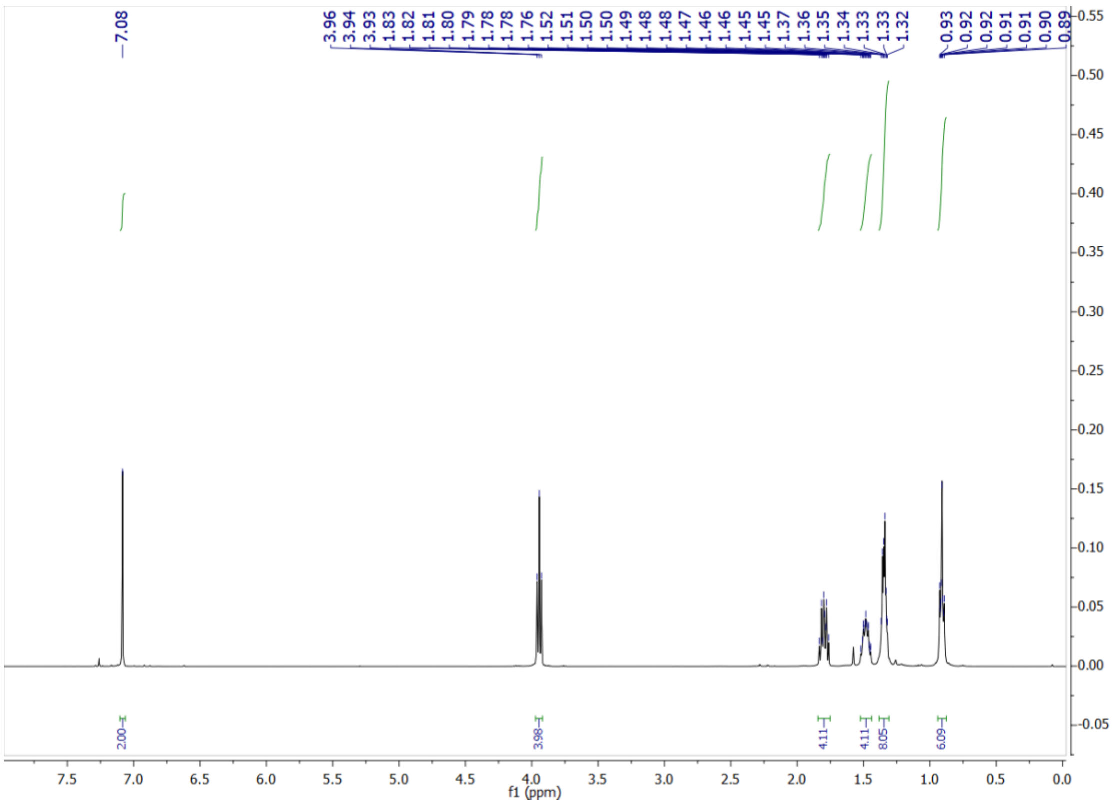

Figure S1: 1H NMR of ***L-1*** *in CDCl3*

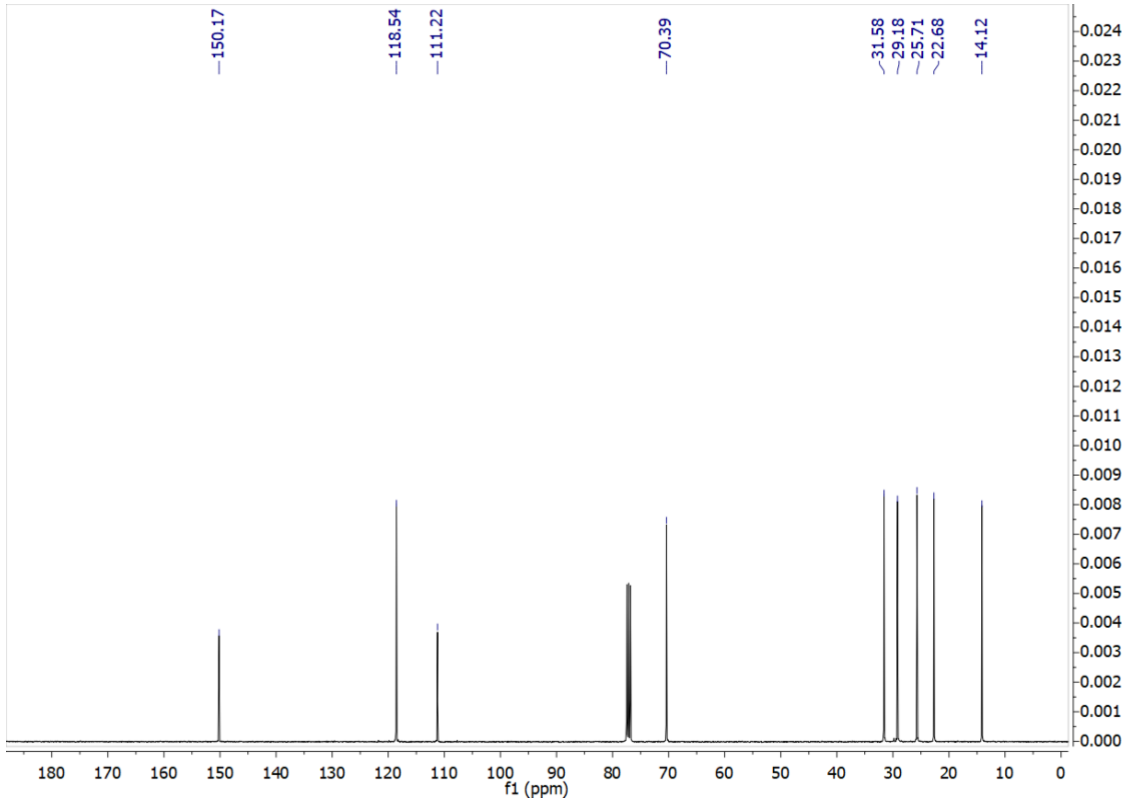

Figure S2: ^{13}C *NMR of* ***L-1*** *in* $CDCl_3$

2.1.2 Synthesis L-2

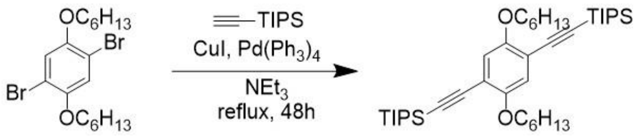

To 1,4-dibromo-2,5-bis(hexyloxy)benzene (2 g, 6.76 mmol) was added Pd(PPh3)4 (64 mg, 0.09 mmol, 2 mol %), CuI (15 mg, 0.7 mmol, 4 mol %) and triisopropylsilylacetylene (3.7 mL, 16.5 mmol, 3.5 eq). NEt_3 (20 mL) was then added, and the reaction mixture was refluxed for 60 h under an inert Ar environment. After the reaction, the black mixture was evaporated and the crude residue was purified by column chromatography (pentane then DCM:pentane, 50:50) followed by a recrystallization in MeOH affording a yellow powder (2.35g, 55% yield).

^{1}H NMR (400 MHz) δ: 7.09 (s, 2H), 3.95 (t, J = 6.5 Hz, 4H), 1.84 – 1.76 (m, 4H), 1.52 – 1.44 (m, 4H), 1.39 – 1.29 (m, 8H), 0.91 (t, 6H) ppm.

^{13}C NMR (101 MHz, Chloroform -D) δ: 154.14, 117.20, 114.12, 103.17, 96.30, 69.45, 31.79, 29.53, 25.93, 22.73, 18.79, 14.17, 11.46 ppm.

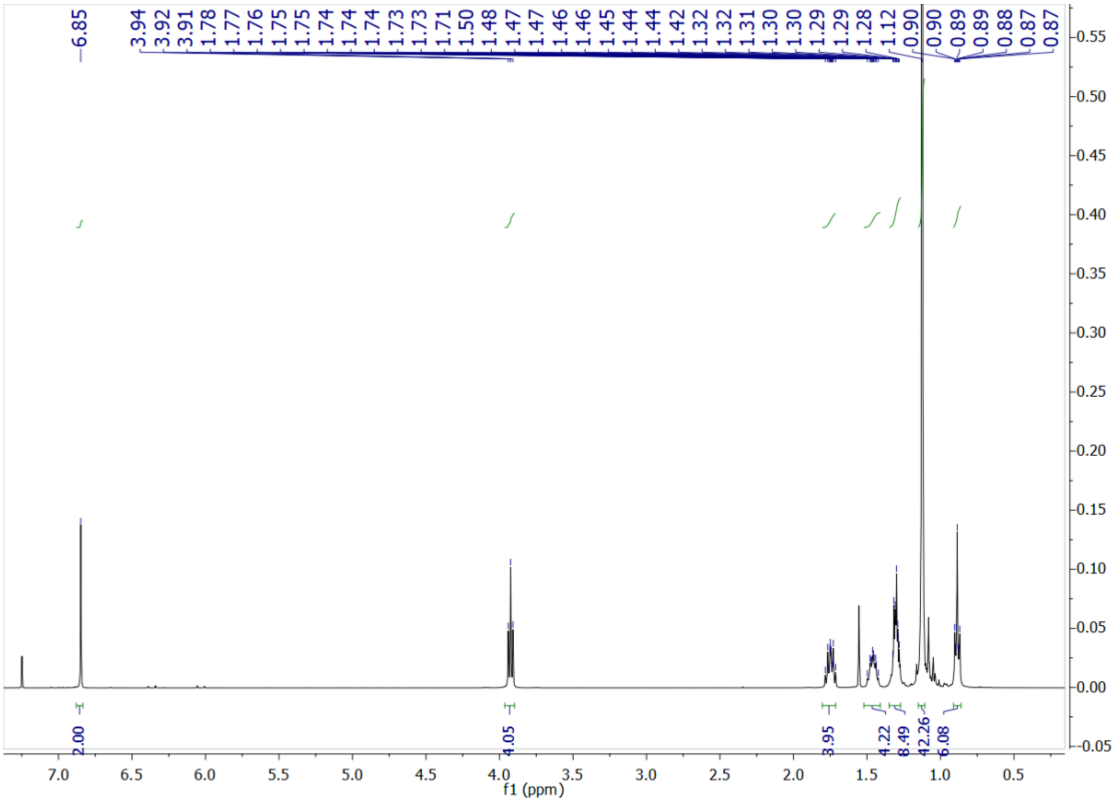

*Figure S3: ^{1}H NMR of **L-2** in $CDCl_3$*

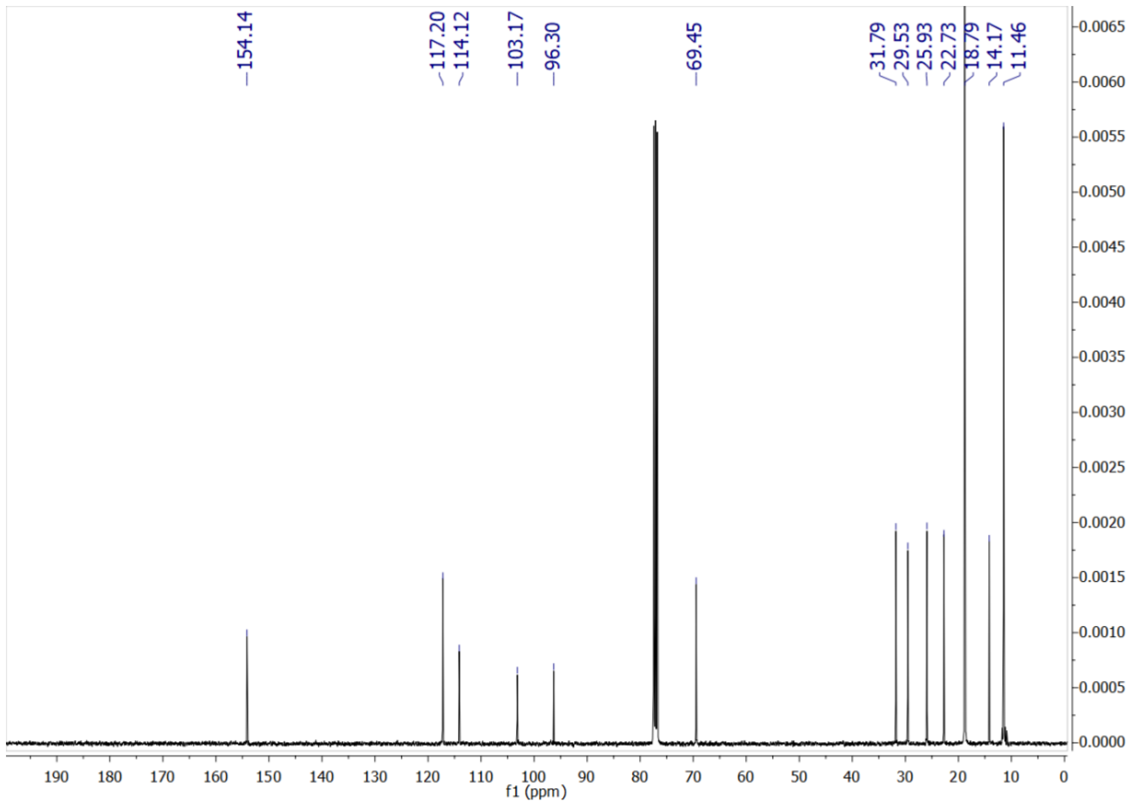

*Figure S4: ^{13}C NMR of **L-2** in $CDCl_3$*

2.1.3 Synthesis L-3:

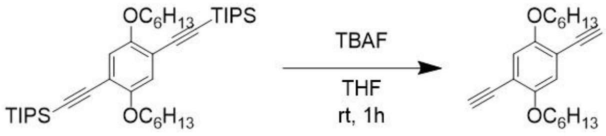

The reactant (1 g, 1,56 mmol, 1eq.) was dissolved in THF (200 mL) and a 1 M tetrabutylammonium fluoride was added to the solution (3.13 mL, 3.13 mmol, 2 eq.). The solution was stirred at room temperature for 1 h The mixture was extracted with dichloromethane and washed with water (2 x 50 mL). The organic phase was dried with Na2SO4 and evaporated. The crude was then purified by column chromatography (Ethyl acetate: pentane, 2:8) to yield a yellow powder (68% yield).

^{1}H NMR (400 MHz, Chloroform-d) δ 6.94 (s, 2H), 3.95 (t, J = 6.6 Hz, 4H), 3.32 (s, 2H), 1.78 (dq, J = 8.3, 6.7 Hz, 4H), 1.50 – 1.43 (m, 4H), 1.36 – 1.29 (m, 8H), 0.91 – 0.85 (m, 6H) ppm.

^{13}C NMR (101 MHz, Chloroform -D) δ: 154.09, 117.82, 113.35, 82.55, 79.90, 69.77, 31.64, 29.20, 25.70, 22.71, 14.15 ppm.

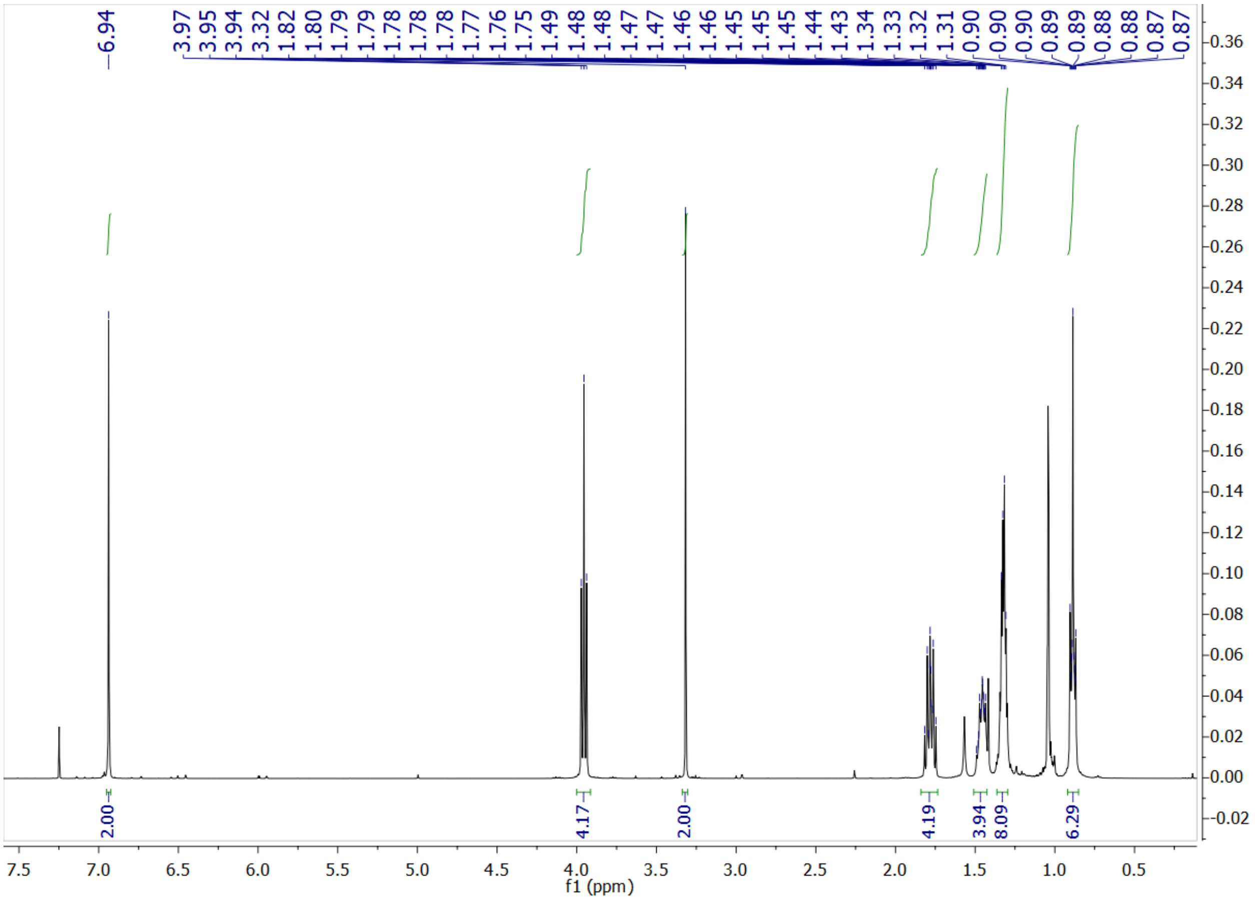

Figure S5: [1]*H NMR of* ***L-3*** *in CDCl*$_3$

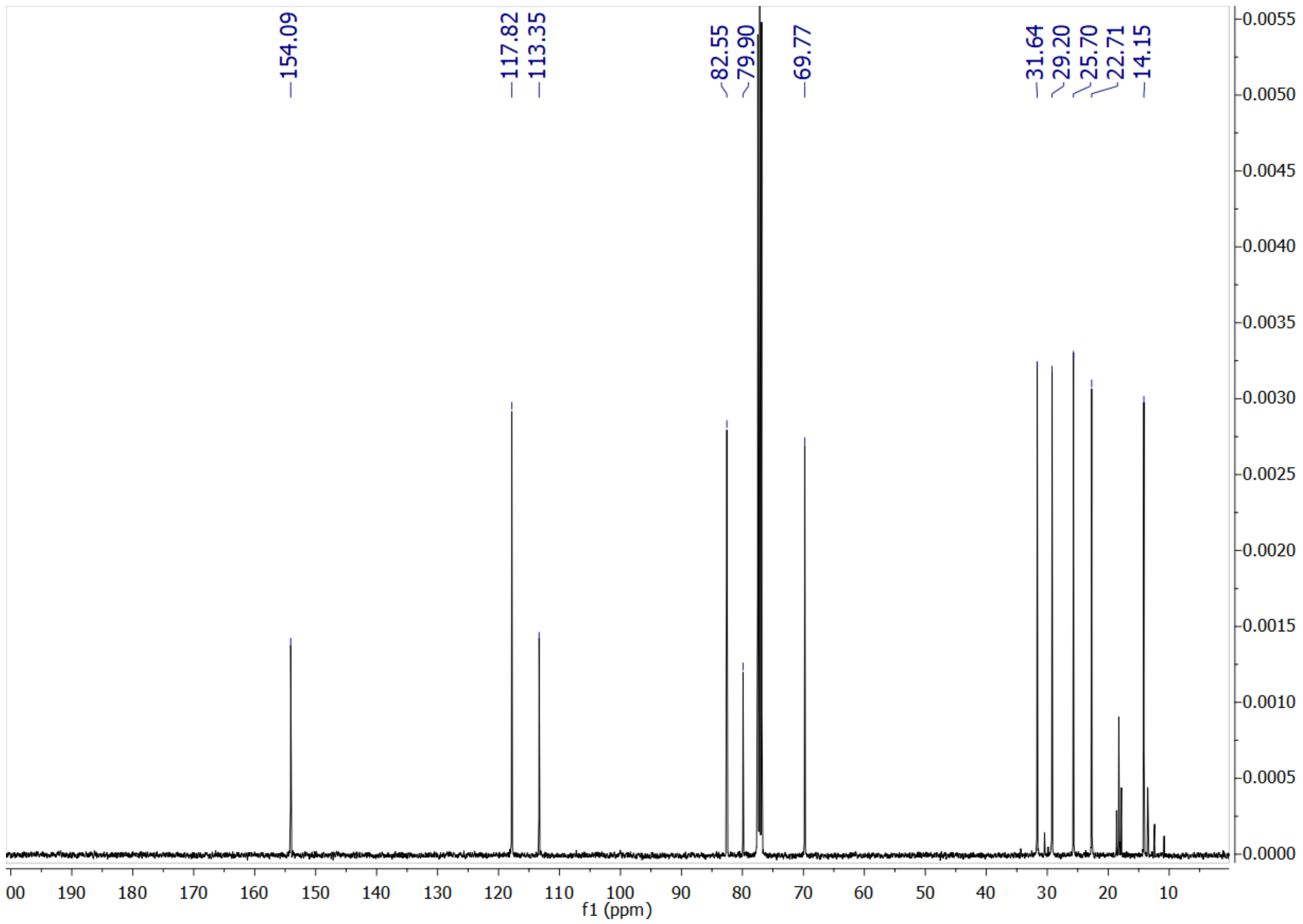

*Figure S6: ^{13}C NMR of **L-3** in $CDCl_3$*

2.1.4 Synthesis Tcdimer:

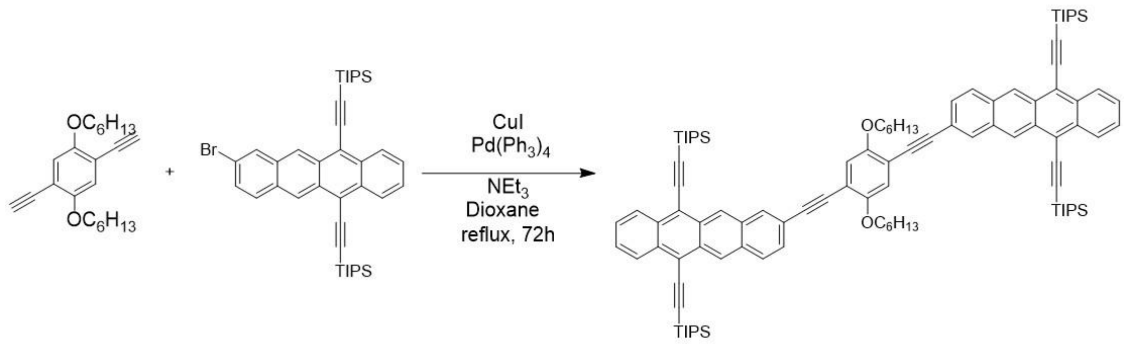

Tc-Br (204mg, 0.3 mmol, 2 eq) was dissolved in 15 mL of dioxane. Once complete dissolution, $Pd(Ph_3)_4$ (10.5 mg, 0.01 mmol, 5 mol%) and CuI (1.5 mg, 0,02 mmol, 6 mol%) were added to the media, followed by 1,4-diethynyl-2,5-bis(hexyloxy)benzene (50 mg, 1 eq, 0.15 mmol) and 10 mL of triethylamine. The media was then heated at 80ºC for 72 h, under inert Ar environment. After the reaction, the black mixture was evaporated to remove the solvent, followed by dissolution in DCM and washing with brine. The crude residue was purified by column chromatography (DCM:heptane, 2:8) to yield a dark red solid (129 mg, 56 % yield).

^{1}H NMR (400 MHz, Chloroform-d) δ: 9.28 (d, J = 3.4 Hz, 4H), 8.65 – 8.62 (m, 4H), 8.24 (s, 2H), 7.99 (d, J = 8.9 Hz, 2H), 7.59 – 7.56 (m, 4H), 7.56 (s, 1H), 7.54 (d, J = 1.5 Hz, 1H). δ 7.17 (s, 2H), 4.15 (t, J = 6.5 Hz, 4H), 1.96 (q, J = 8.3, 6.5 Hz, 4H), 1.62 (td, J = 9.3, 8.5, 4.5 Hz, 4H), 1.50 – 1.39 (m, 8H), 1.37 – 1.31 (m, 96H), 0.91 (t, J = 7.1 Hz, 6H) ppm.

^{13}C NMR (101 MHz, Chloroform -D) δ: 153.97, 132.95, 132.90, 132.21, 131.65, 131.15, 130.82, 130.78, 128.79, 128.31, 127.51, 127.01, 126.56, 126.48, 120.91, 118.84, 117.00, 114.17, 106.30, 106.13, 103.83, 96.01, 87.88, 69.81, 31.72, 29.41, 25.87, 22.79, 19.07, 19.05, 14.16, 11.68 ppm.

IR (cm^{-1}): 2960, 2940, 2860 (C-H: bending aromatic); 2145, 2120 (C-C triple bound : bending)

HRMS (MALDI-TOF) *m*/*z*: theor: 1498.91 found: 1499.917 ($[M]^{+\cdot}$ detected).

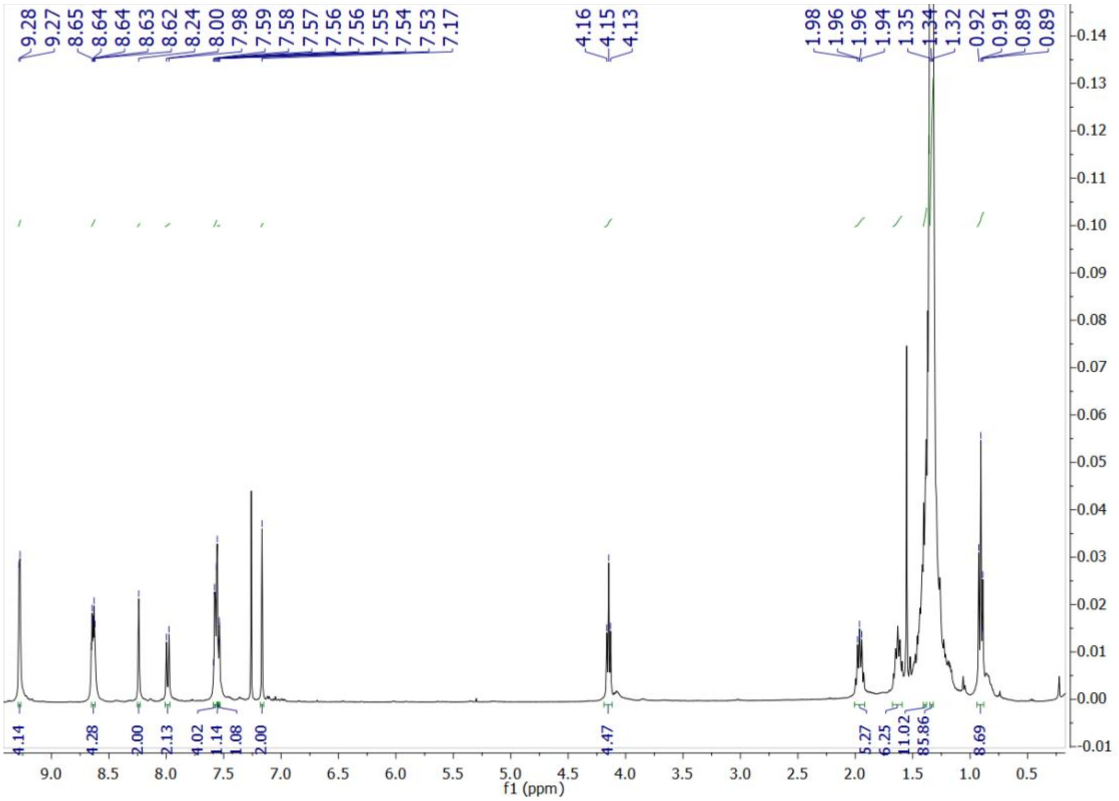

*Figure S7: ^1H NMR of **Tcdimer** in $CDCl_3$*

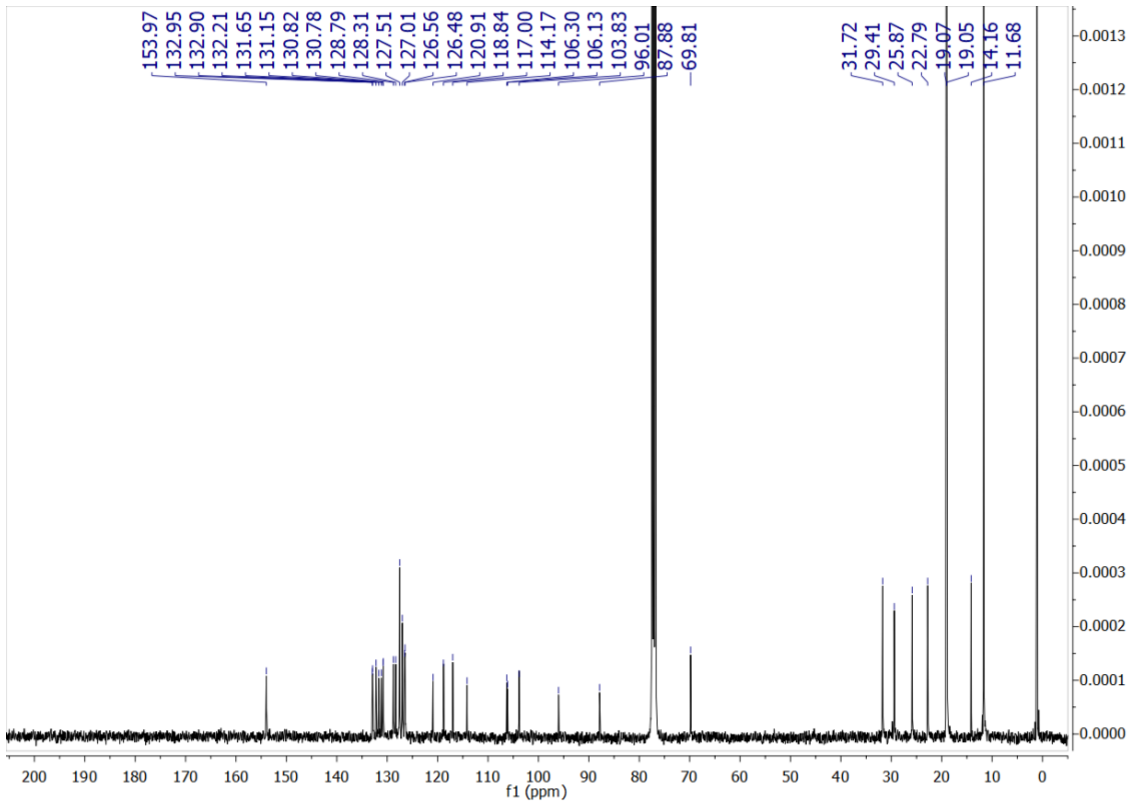

*Figure S8: ^{13}C NMR of **Tcdimer** in $CDCl_3$*

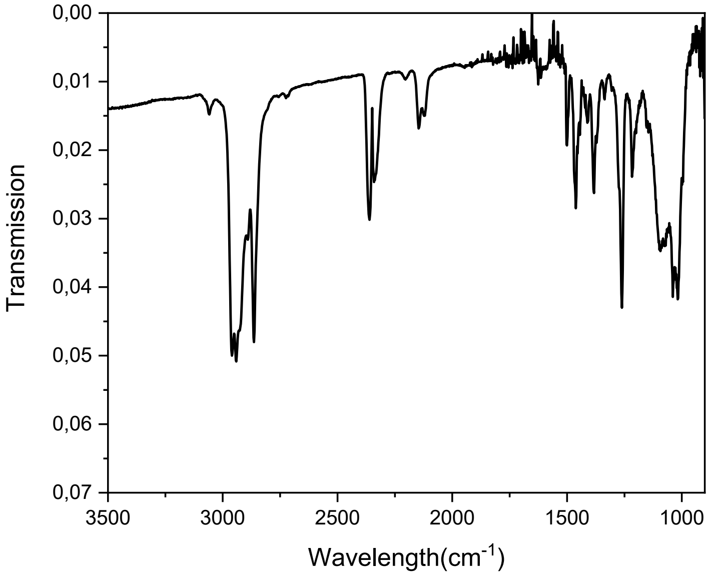

*Figure S9: FTIR spectrum of **Tcdimer***

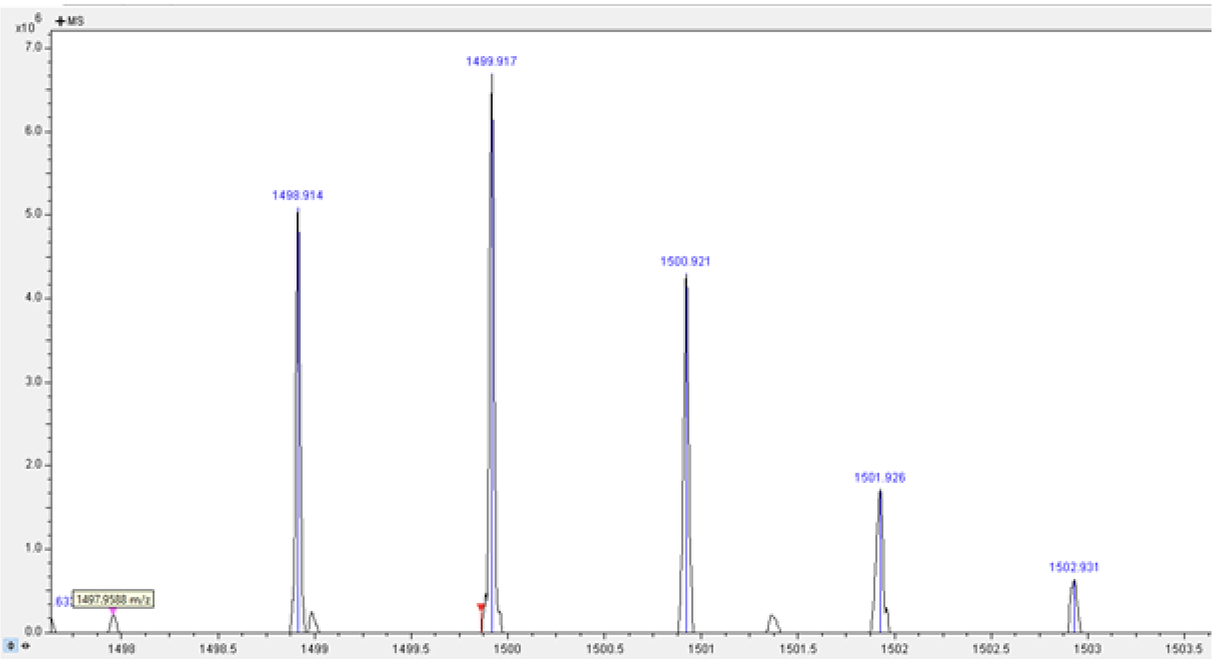

*Figure S10: HRMS (MALDI-TOF) results of **Tcdimer***

2.1.5 Synthesis Tcmonomer:

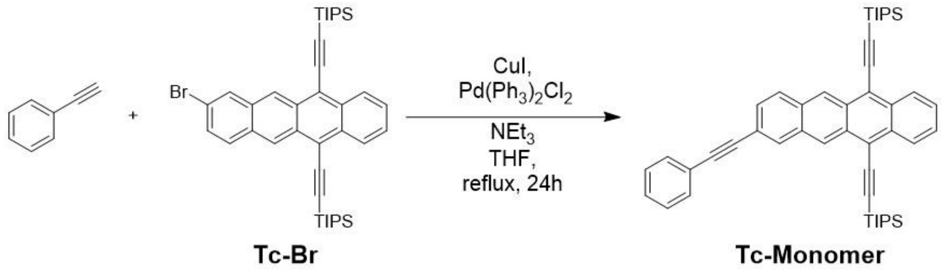

Tc-Br (100 mg, 0.15 mmol, 1 eq) was dissolved in 15 mL of THF. Once complete dissolution, $Pd(Ph_3)_4$ (10.5 mg, 0.01 mmol, 5 mol%) and CuI (1.5 mg, 0,02 mmol, 6 mol%) were added to the media, followed by phenylacetylene (15 mg, 1.1 eq, 0.15 mmol) and 15 mL of triethylamine. The media was then heated at reflux overnight, under inert Ar environment. After the reaction, the black mixture was evaporated to remove the solvent, followed by dissolution in DCM and washing with brine. The crude residue was purified by column chromatography (DCM:heptane, 2:8) followed by a recrystallisation in a toluene/ACN mixture (60/40) to yield a dark red solid (45 mg, 44 % yield).

^{1}H NMR (400 MHz, Chloroform-d) δ 9.26 (d, J = 3.8 Hz, 2H), 8.62 (d, J = 7.6 Hz, 2H), 8.20 (s, 1H), 7.97 (d, J = 8.9 Hz, 1H), 7.63 (m, 2H), 7.55 (dd, J = 6.8, 3.2 Hz, 2H), 7.51 (d, J = 9.0 Hz, 2H), 7.39 (d, J = 6.5 Hz, 3H) ppm.

^{13}C NMR (101 MHz, Chloroform -D) δ: 132.89, 132.27, 131.85, 131.60, 131.12, 130.77, 128.84, 128.54, 128.25, 127.51, 127.01, 126.52, 123.26, 120.71, 118.87, 106.34, 103.80, 90.26, 19.07, 19.04, 11.67 ppm.

HRMS (MALDI-TOF) *m*/*z*: theor: 688.39 found: 688.39166 ($[M]^{+\cdot}$ detected).

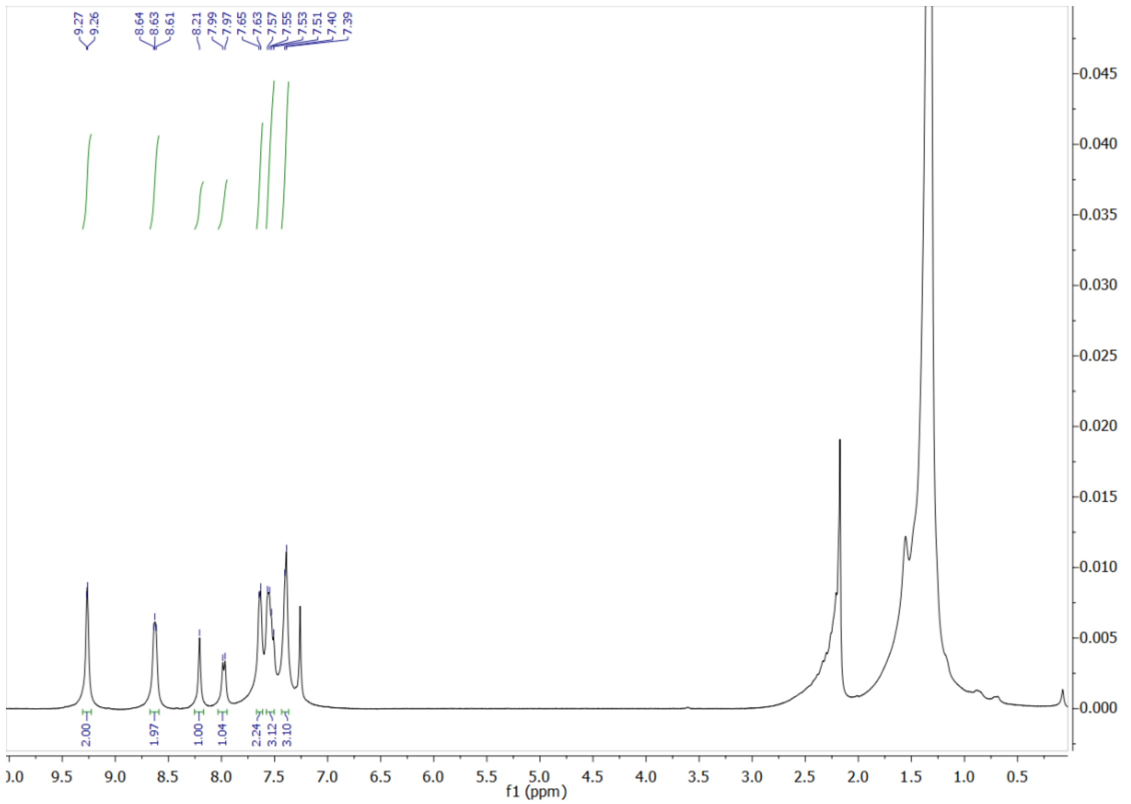

*Figure S11: ^{1}H NMR of **Tcmonomer** in $CDCl_3$*

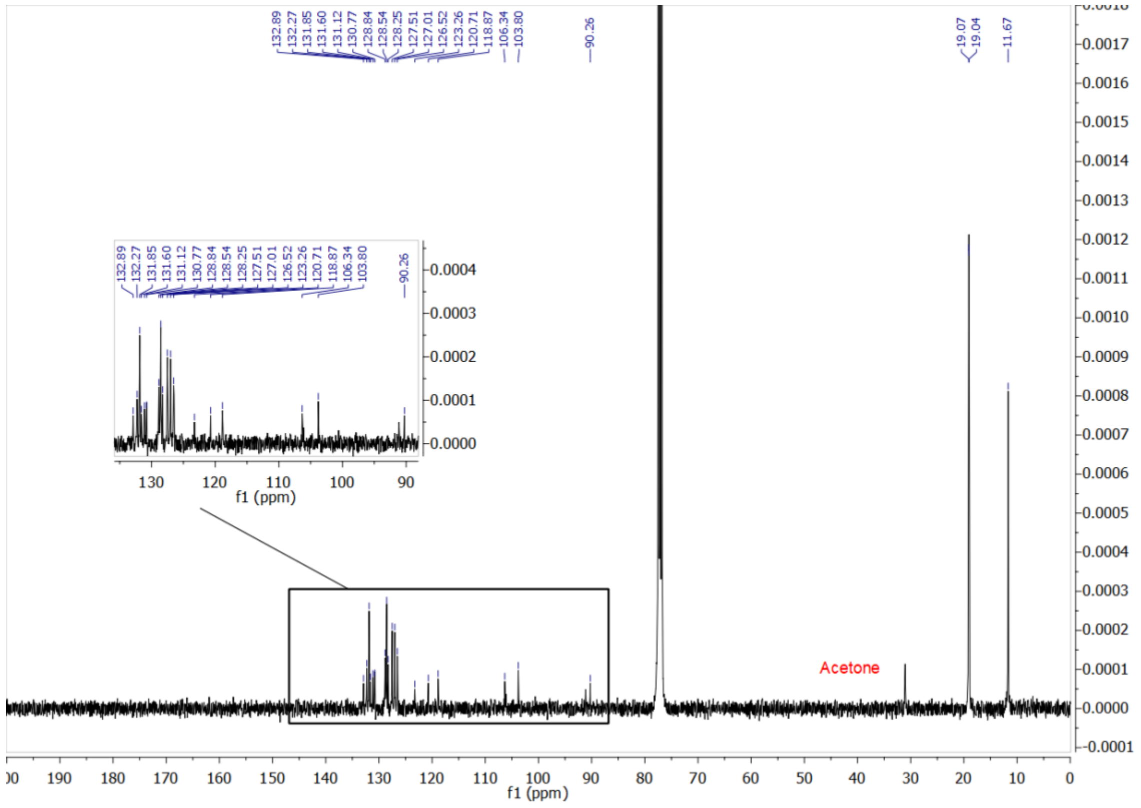

*Figure S12: ^{13}C NMR of **Tcmonomer** in $CDCl_3$*

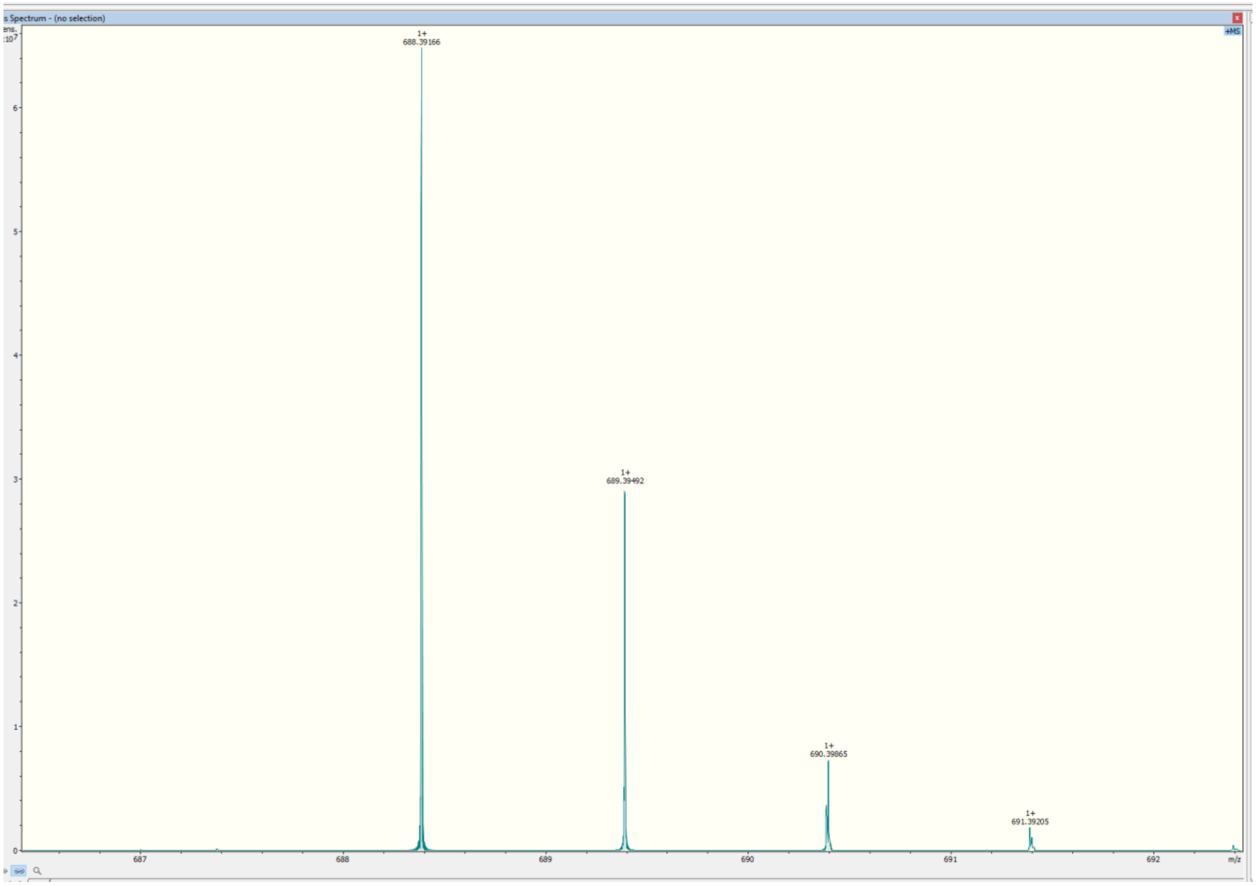

Figure S13: HRMS (MALDI-TOF) results of ***Tcmonomer***

2.2 Synthesis of Tc-BP-Tc:

For the biphenyl tetracene dimer **Tc-Br** was first converted to the boronicacid pinacoleester following Sanders et al.[6] and then used in a Suzuki coupling reaction with 2,2'-dibromobiphenyl following the procedure by Nakamura et al.[1]

2.2.1 Synthesis of Tc-boronicacid pinacole ester (Tc-Bpin):

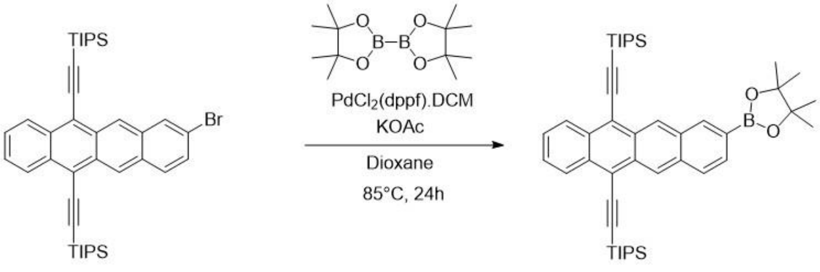

Tc-Br **Tc-BPin**

Tc-Br (1.0 g, 1.5 mmol), bis(pinacolato)diboron (0.6 ml, 2.3 mmol), Pd(dppf)Cl_2(DCM) (60 mg, 0.07 mmol) and KOAc (0.52 g, 5.3 mmol) was added to a dry Schlenck tube. The tube was degassed by sequential vacuum and N_2, followed by the addition of dry and degassed dioxane (7 ml). The mixture was heated to 85 °C overnight in the dark. The reaction mixture was transferred to 50 mL DCM and washed with water. The aqueous layer was extracted with DCM (50 mL). The combined organic phase was dried over Na_2SO_4, filtered and solvent removed under reduced pressure. The crude reaction mixture was purified by silica column chromatography using a mixture of hexanes and DCM as eluent to yield 715 mg (32% yield) of bright red product. Spectrocopic data conformed to literature.[6]

^{1}H NMR (400 MHz, Chloroform-d) δ: 9.32 (s, 1H), 9.27 (s, 1H), 8.66 – 8.60 (m, 2H), 8.55 (s, 1H), 7.96 (d, J = 8.6 Hz, 1H), 7.78 – 7.74 (m, 1H), 7.56 – 7.51 (m, 2H), 1.43 (s, 12H), 1.34 – 1.30 (m, 38H) ppm.

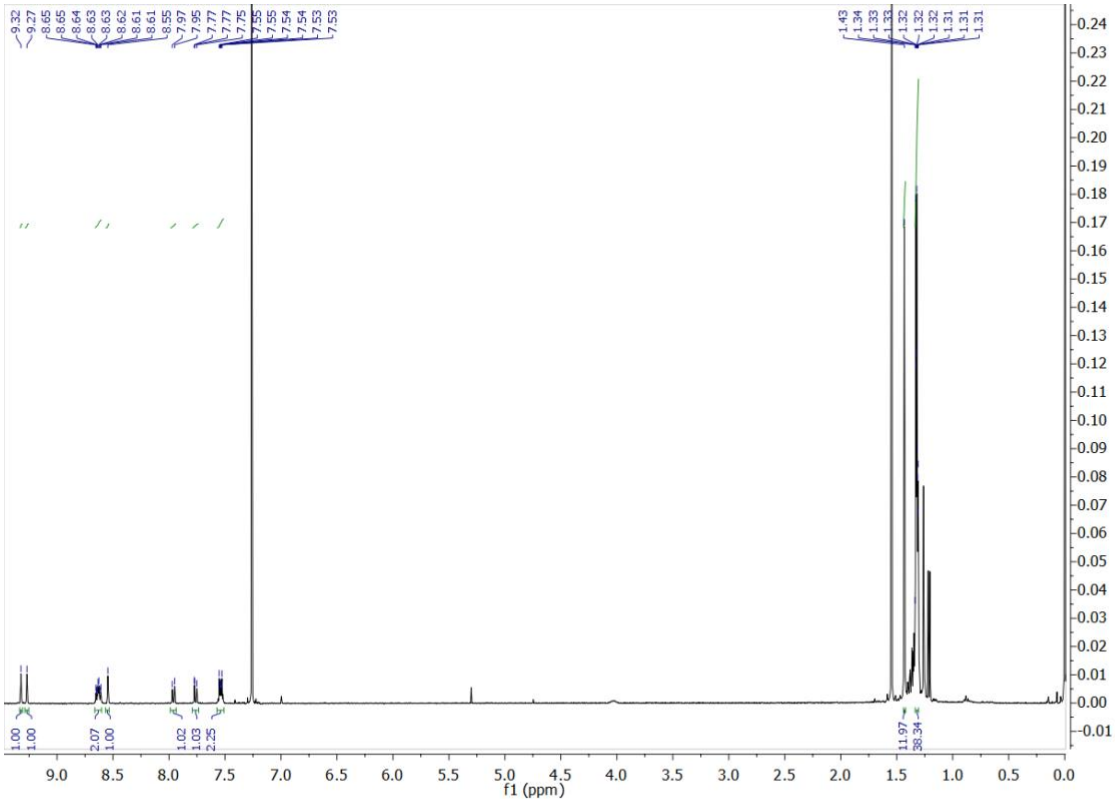

*Figure S14: ^{1}H NMR of **Tc-Bpin** in $CDCl_3$*

2.2.2 Synthesis of Tc-BP-Tc:

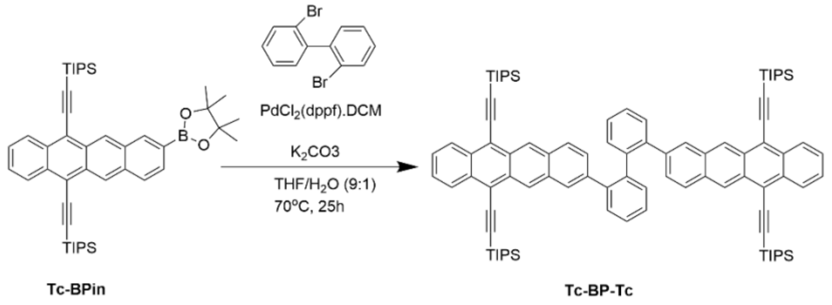

Tc-BPin (200 mg, 0.28 mmol), 2,2'-dibromobiphenyl (29.4 mg, 0.094 mmol), $PdCl_2(dppf)(DCM)$ (40 mg, 0.05 mmol), and K_2CO_3 (664 mg, 4.480 mmol) were transferred to a Schlenck flask and the atmosphere exchanged for N_2 through repeated vacuum and N_2. Deaerated THF (30 mL) and deaerated H_2O (3 mL) was added to the flask. The reaction mixture was purged with N_2 for an additional 20 min and then stirred for 25 h at 70 °C in the dark. After cooling to room temperature, the reaction was extracted with DCM and washed with water, the organic phase was dried over Na_2SO_4 and solvent was removed by evaporation. The obtained crude was purified by silica gel column chromatography using mixture of hexane and DCM as an eluent (hexane/DCM = 20/1, v/v). The product was purified by recrystallization and 70 mg (55%) was obtained as a red powder. HRMS, UV-Vis and transient absorption spectra conformed to expected and reported data, however ^{1}H NMR did not match that reported by Nakamura et al, and the reported C-NMR is very noisy rendering comparison difficult.[1] However, our ^{1}H NMR and ^{13}C NMR together with COSY-NMR strongly agree with the suggested dimer structure (see assignment and

figures S15-S18 below). In the ^{13}C 31 signal could be found which is 1 less than expected. It is likely that the signal at 130.51ppm is an overlay of two signals since its intensity is significantly higher than for other aromatic signals.

^{1}H NMR (400 MHz, Chloroform-d) δ: 9.15 (s, 2H), 8.86 (s, 2H), 8.68 – 8.47 (m, 4H), 7.59 (d, J = 9 Hz, 2H), 7.55 – 7.48 (m, 6H), 7.42 – 7.31 (m, 8H), 6.79 (dd, J = 9, 1.7 Hz, 2H), 1.29 (s, 42H), 1.12 (bs, 42H) ppm.

^{13}C NMR (101 MHz, Chloroform -D) δ: 140.66, 140.05, 138.56, 132.61, 132.56, 132.03, 132.00, 130.93, 130.51, 130.47, 128.75, 128.29, 127.89, 127.78, 127.75, 127.51, 127.43, 126.63, 126.59, 126.29, 125.69, 118.61, 118.52, 105.78, 105.69, 104.08, 103.82, 19.01, 18.91, 11.67, 11.53 ppm.

HRMS (MALDI-TOF):) *m/z*: theor: 1326.76791 found: 1326.76805 ($[M]^{+\cdot}$ detected).

Table S2*. Assignment of ^{1}H-NMR peaks of Tc-biphenyl dimer as labeled in Figure S15, note the color coding.*

Shift, ppm	Multiplicity	Coupling constant, Hz	Integral	Assignment
**9.15**	s		1	12
**8.86**	s		1	1
**8.68-8.47**	m		2	10, 7
**7.59**	d	9	1	3
**7.55-7.48**	m		3	9, 8 and 6 or 3
**7.42-7.31**	m		4	5, 4 and 6 or 3 and 5
**(7.40)**	s		1	5, visible in COSY only
**6.79**	dd	9, 1.7	1	4
**1.29**	bs		21	TIPS
**1.12**	bs		21	TIPS

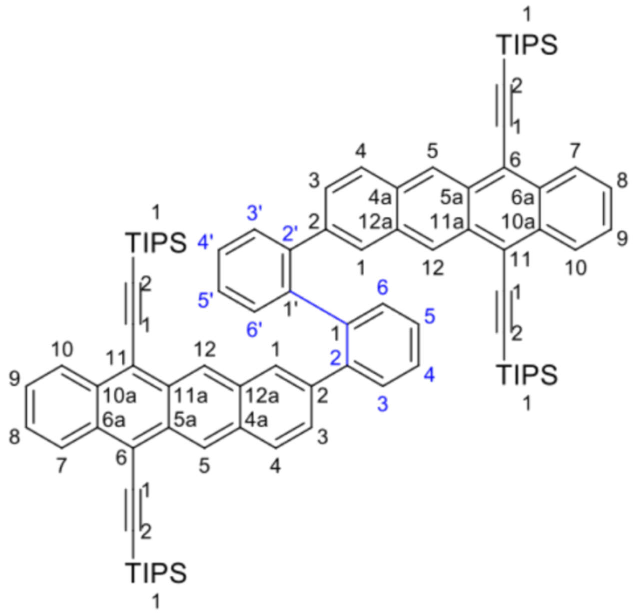

Figure S15: Atom labeling of ***Tc-BP-Tc***

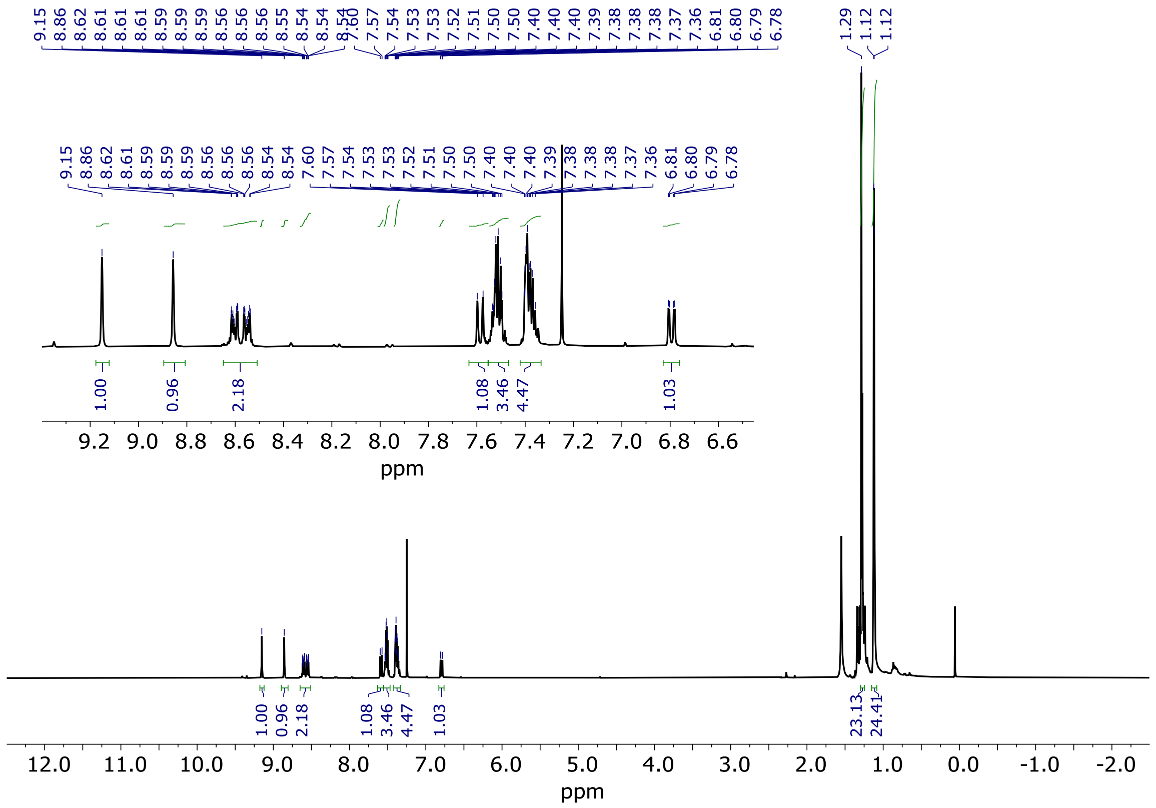

*Figure S16: ^{1}H-NMR of **Tc-BP-Tc** in $CDCl_3$.*

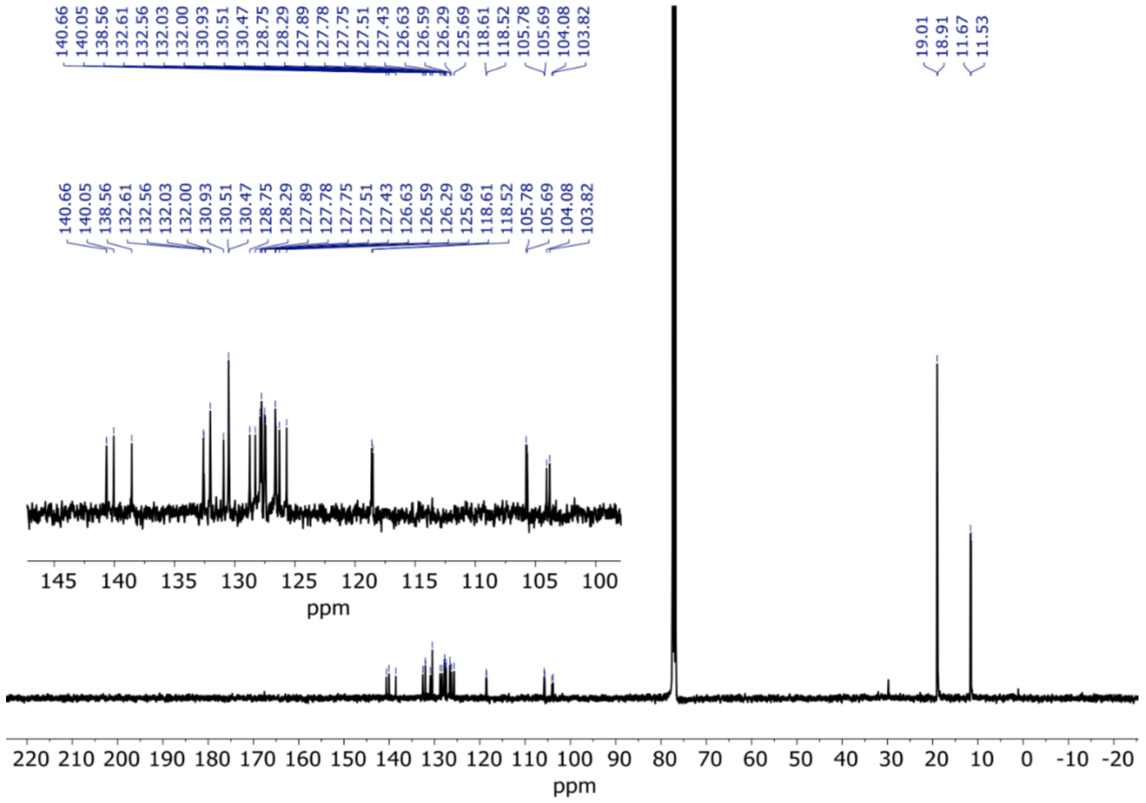

*Figure S17: ^{13}C-NMR of **Tc-BP-Tc** in $CDCl_3$.*

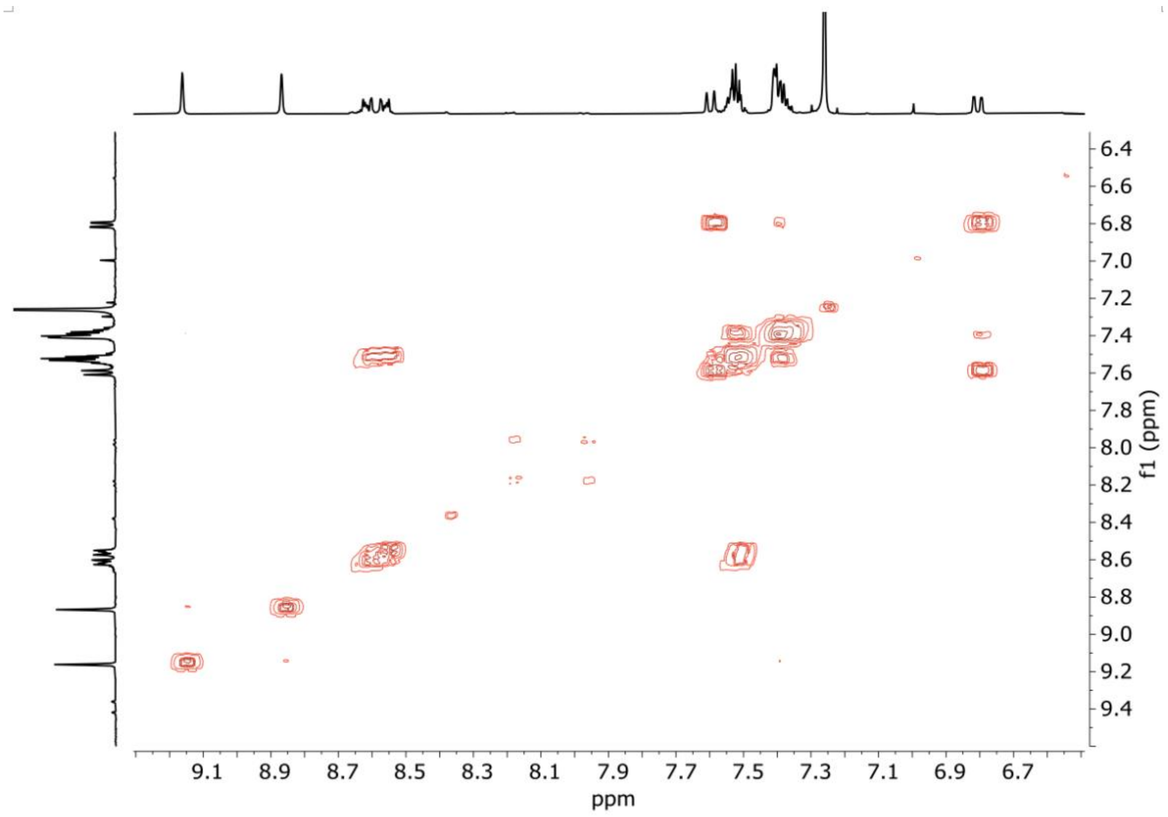

*Figure S18: COSY-NMR of **Tc-BP-Tc** in $CDCl_3$.*

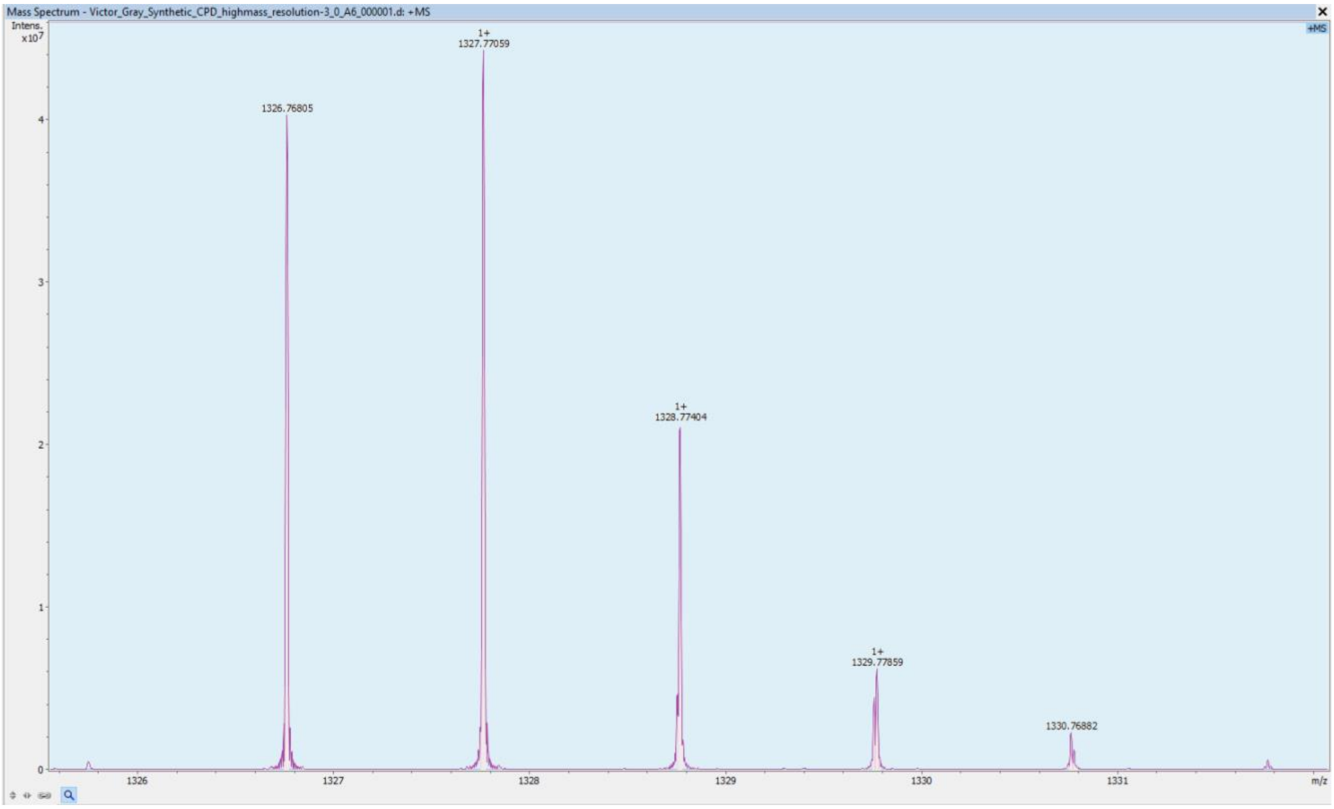

*Figure S19 : HRMS (MALDI-TOF) results of **Tc-BP-Tc***

3. Uv-Vis absorption and fluorescence

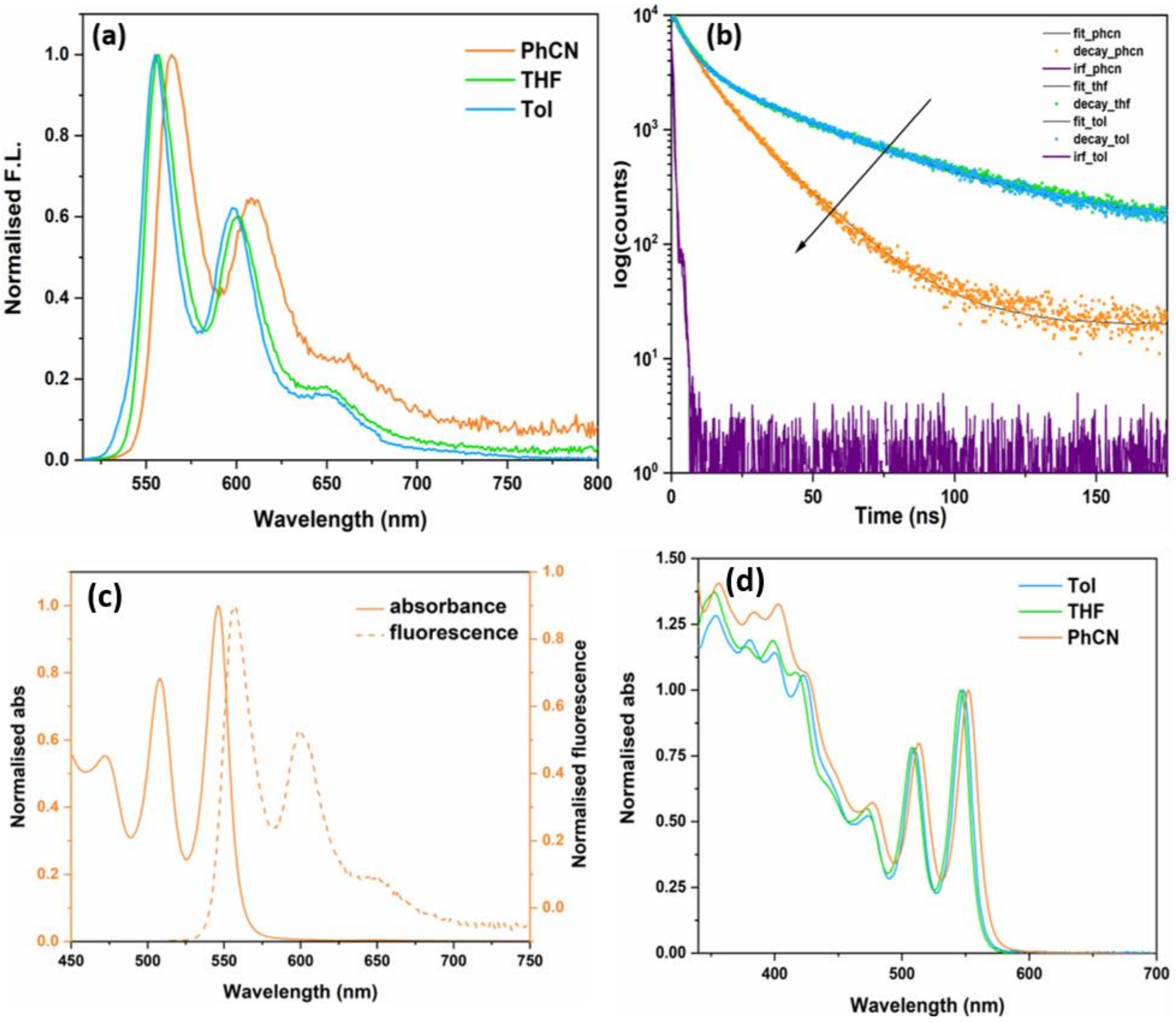

*Figure S20: (a) Normalised fluorescence spectra of **Tcdimer** in different solvents. (b) Time-resolved fluorescence decays of **Tcdimer** in different solvents (c) Normalised absorption and fluorescence of **Tcdimer** in THF, the 0-0 transition occurs at 551 nm.(d) Steady state absorption of **Tcdimer** in different solvents.*

***Table S3**. Fluorescence quantum yields and lifetimes of **Tcdimer** in different solvents.*

Solvent	F.L. QY (%)	Lifetimes (ns)	Relative amplitudes
Toluene	20.6 ± 2.4	6.29, 32.66, 101.52	0.57,0.35,0.069
THF	16.5 ± 1.6	6.44, 32.85,104.73	0.63, 0.29,0.065
PhCN	14.5 ± 1.2	6.2, 16.47,105.04	0.44, 0.54, 0.0067

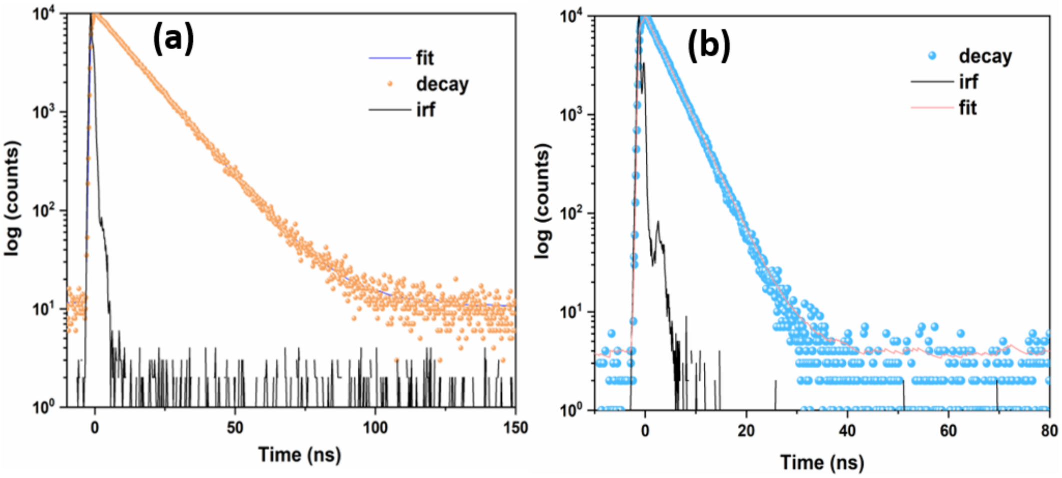

*Figure S21: Time resolved fluorescence decays of **TIPS-Tc** (a) and **Tcmonomer** in THF. (b). Monoexponential fits yield lifetimes of (a) 12.8 ns and (b) 3.85 ns.*

4. Electrochemistry

Cyclic voltammograms of **TCAQ**, and **Tcdimer** are shown in figures S22 and S23, respectively. The spectra of the reduced **Tcdimer** is shown in Figure S24.

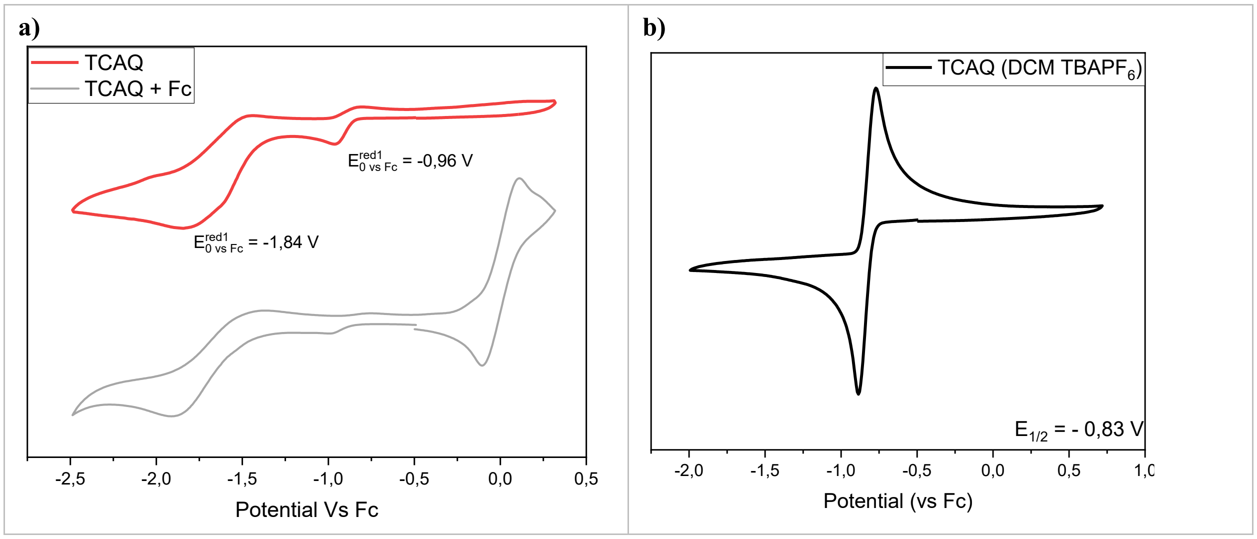

*Figure S22: Cyclic voltammetry of Tetracyanoantraquinone (**TCAQ**) in a) THF with 0.1M of TBAPF$_6$ b) DCM with 0.1M of TBAPF$_6$*

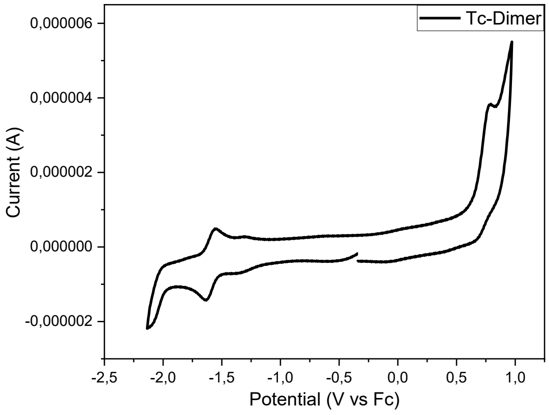

*Figure S23: Cyclic voltammetry of **Tcdimer** in THF with 0.1M of $TBAPF_6$*

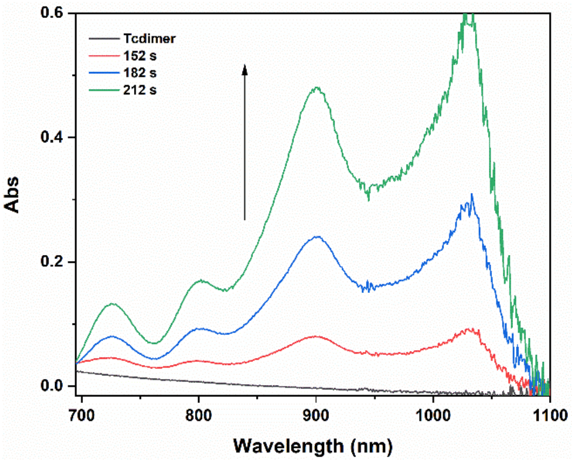

*Figure S24: Spectra of oxidized **Tcdimer** (600 µM, cell length 1 cm) in THF. A potential of 0.8 V vs Fc+/Fc was applied to the solution and the change in spectra was recorded at different times.*

5. fsTA data of Tcdimer and Tcmonomer

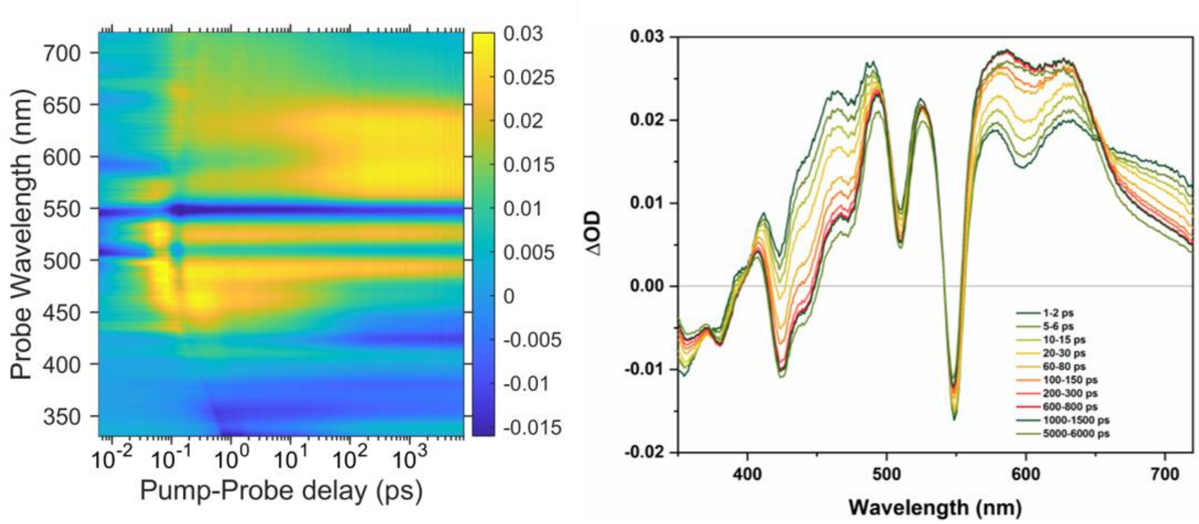

*Figure S25: fsTA 2D map of **Tcdimer** in toluene excited at 500 nm (left) and spectra at selected time points (right).*

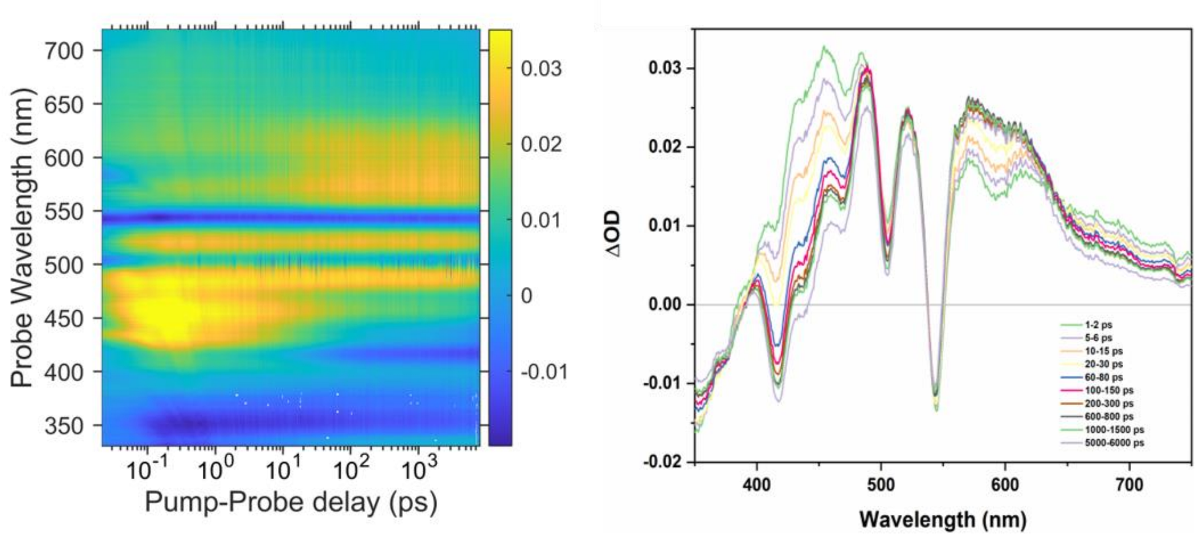

*Figure S26: fsTA 2D map of **Tcdimer** in THF excited at 500 nm (left) and spectra at selected time points (right).*

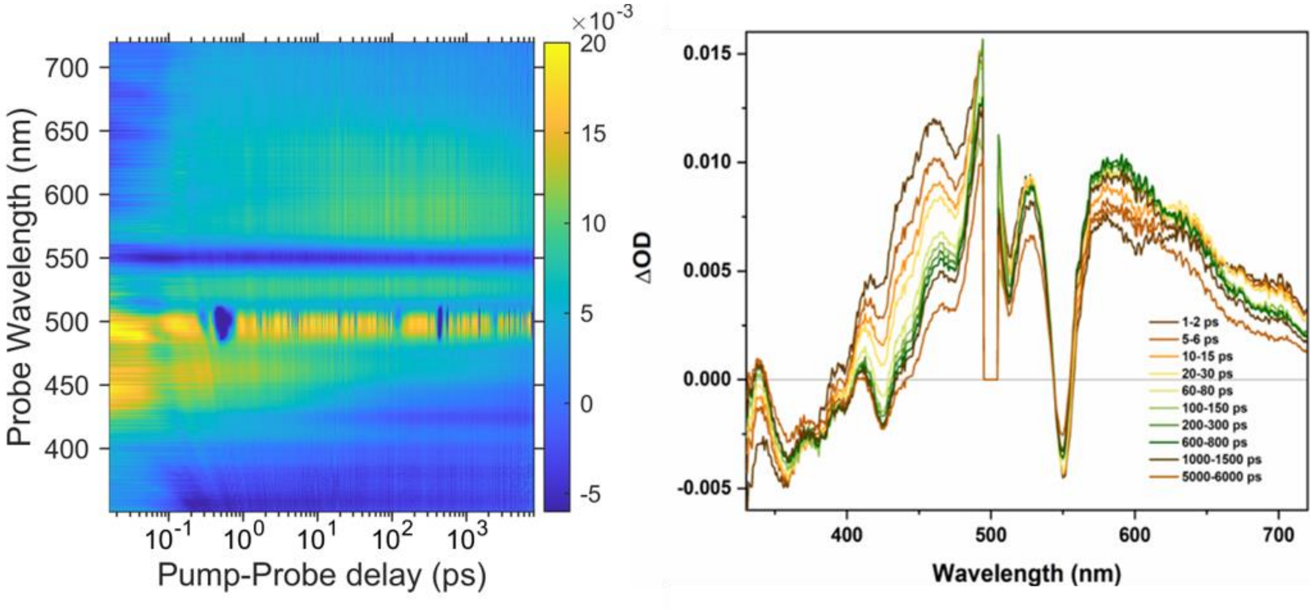

*Figure S27: fsTA 2D map of **Tcdimer** in PhCN excited at 500 nm (left) and spectra at selected time points (right).*

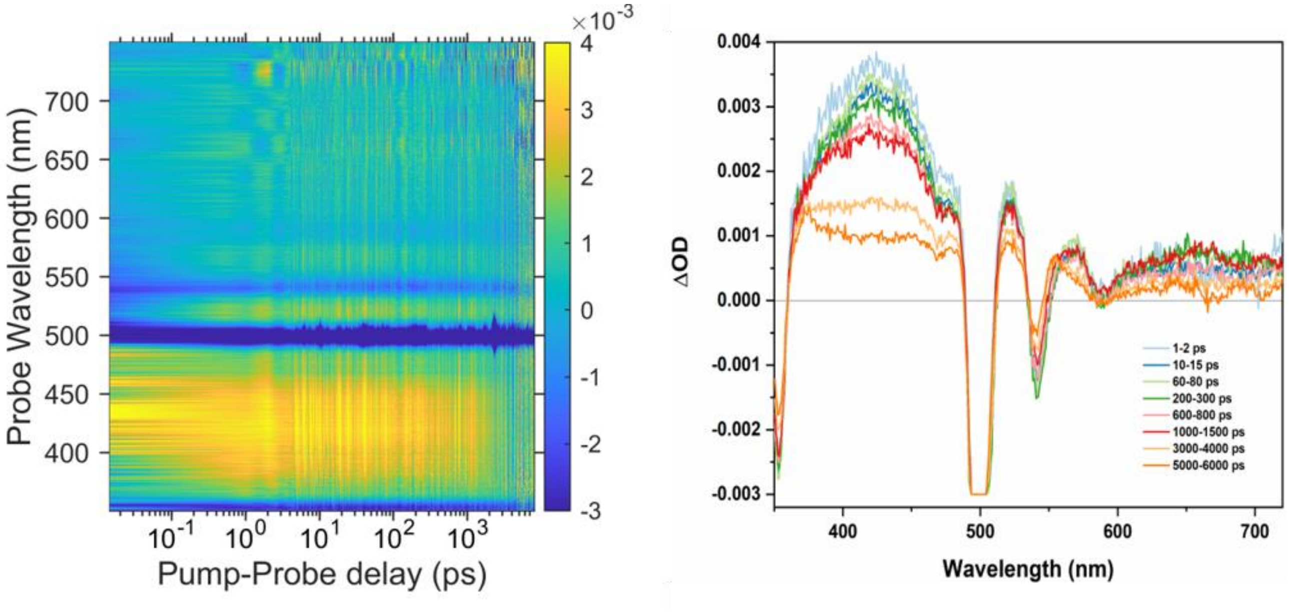

*Figure S28: fsTA 2D map of **Tcmonomer** in THF excited at 500 nm (left) and spectra at selected time points (right).*

6. Global Analysis of fsTA

R package TIMP and its GUI Glotaran was used to perform global analysis by the least-squares fitting method. A three component sequential model (A→B→C) based on a previous paper[8] was used to fit the data. The evolution associated spectra (EAS) are shown in the main text and Figure S29. Based on the interpretation of Rapp *et al.*[8] of a similar tetracene dimer we assign the intermediate component to a charge transfer intermediate $S_1S_0{}^{CT}_{TT}$. This interpretation is supported by the faster formation of $S_1S_0{}^{CT}_{TT}$ and subsequent slower ^1TT formation in the polar solvent PhCN, Table S4. However, if the requirement of this intermediate in the fitting is indeed a result of a stepwise ^1TT formation via a charge transfer state, or a result of rotational dynamics and populations of slightly different coupling is beyond the scope of the current investigation.

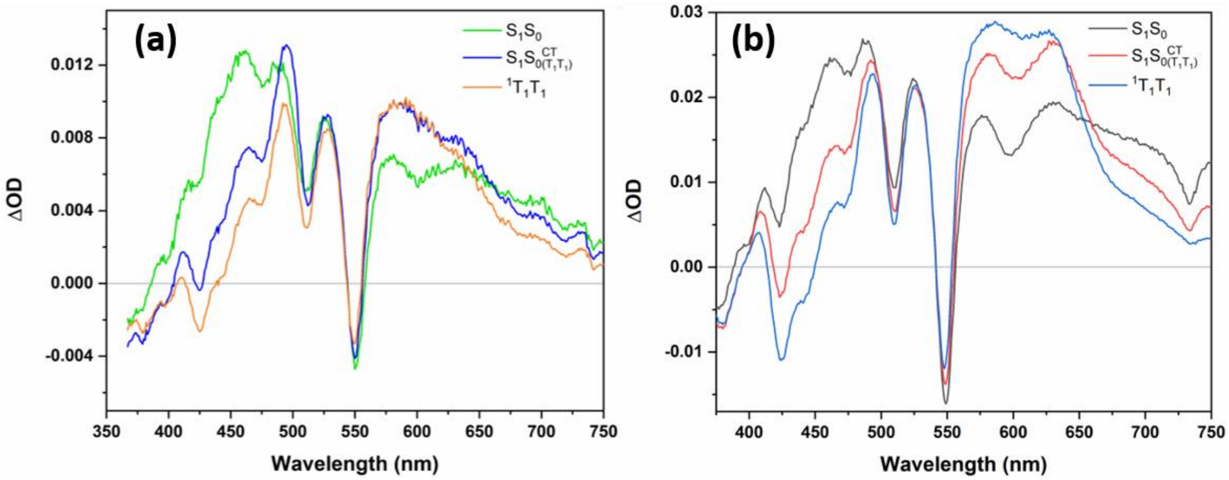

Figure S29: Three state EAS obtained from global analysis of the fsTA data of ***Tcdimer*** *in (a) PhCN and (b) Toluene upon excitation at 500 nm.*

Table S4*: Time constants summarized from fitting by global analysis of fsTA data of* ***Tcdimer*** *in different solvents*

Solvent	(S_1S_0) (ps)	$S_1S_0{}_{TT}^{CT}$ (ps)	$^1(T_1T_1)$ (ns)
Toluene	19.2	188.7	87.1
THF	14.2	151.5	53.3
PhCN	11.2	515	22.9

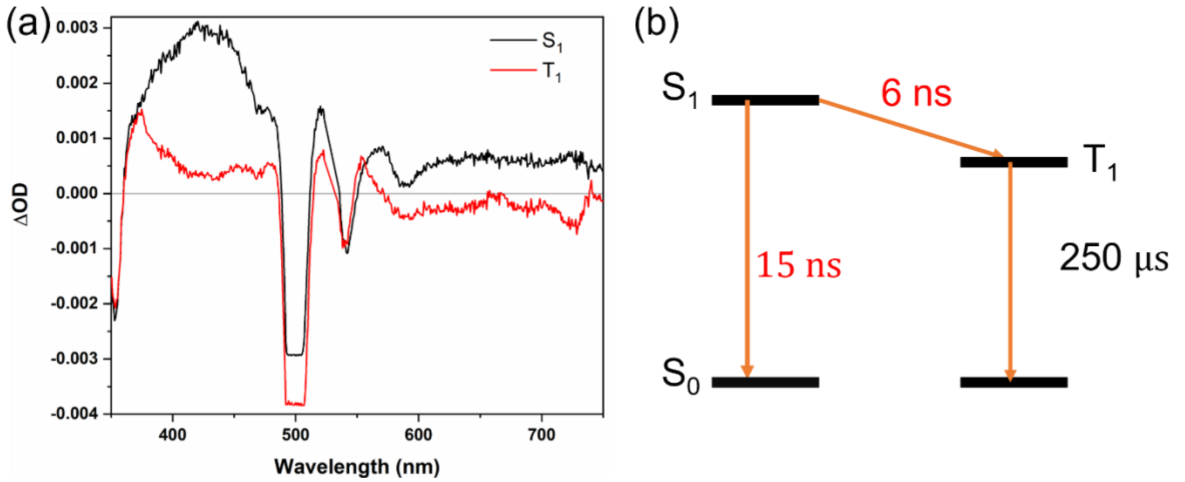

Figure S30: (a) Two state EAS obtained from global analysis of the fsTA data of ***Tcmonomer*** *in THF (b)Model used to fit the data, the values in red represent the time constants that were fitted and the value in black was kept fixed.*

7. nsTA of Tcdimer

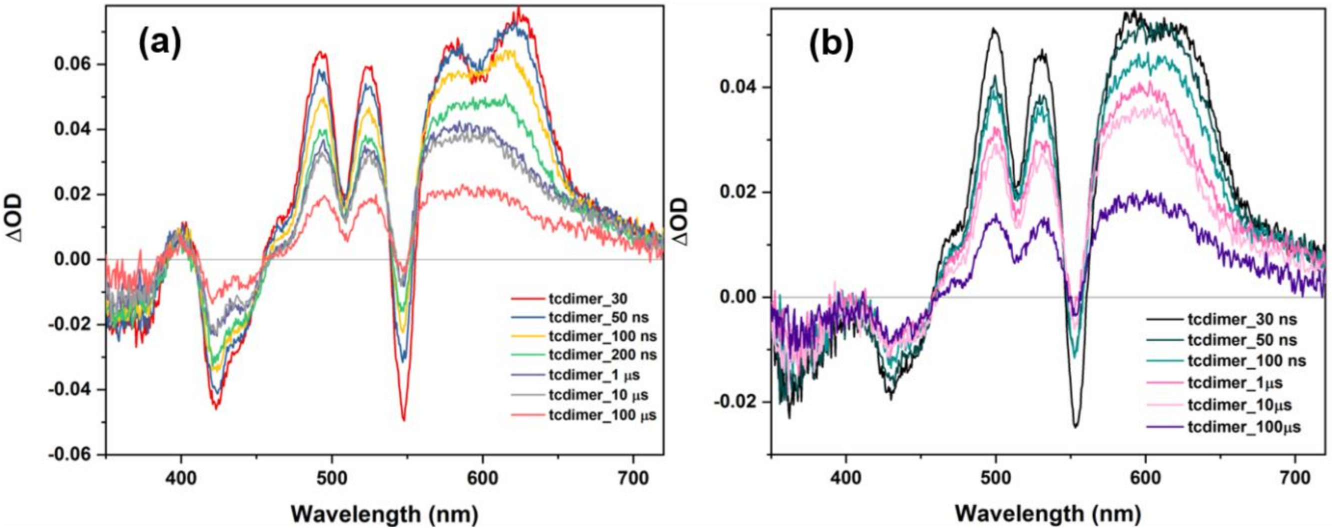

*Figure S31: nsTA spectra of **Tcdimer** when excited at 545 nm in (a) Toluene (b) PhCN*

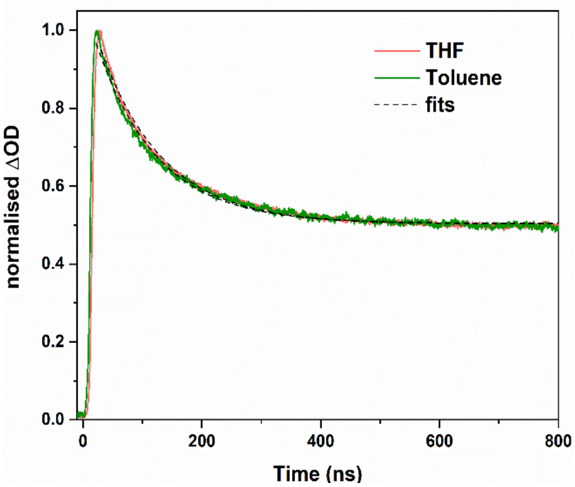

Figure S32: nsTA kinetic profiles at 525 nm of Tcdimer in THF and Toluene, indicating similar final triplet intensity in both solvents.

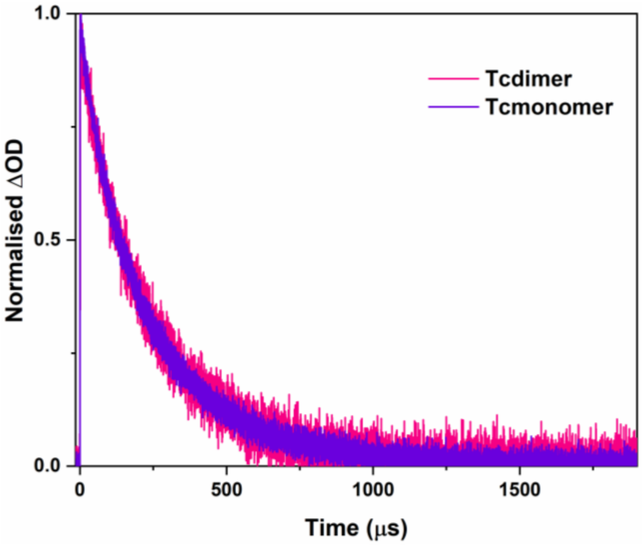

*Figure S33: The time profiles at 525 nm for **Tcdimer** and at 516 nm for **Tcmonomer** show that they decay with a similar time constant of 240 μs in THF.*

8. Determination of the triplet yield of Tcdimer

These nsTA measurements were carried out in 1cm×1cm cuvette in toluene as the solvent. The calculation of triplet yield and triplet extinction coefficients was done at low powers, because at high powers of 6 mJ/ pulse, the triplet decay of the zinc porphyrins (Zinc octaethyl porphyrin, ZnOEP, and Zinc tetraphenyl porphyrin, ZnTPP) was found to be biexponential due to triplet-triplet annihilation, Figure S34a. Hence the experiments were done at low powers of around 120 μJ/pulse, where monoexponential decays were observed, Figure S34b, so that the triplet yield can be estimated more accurately.

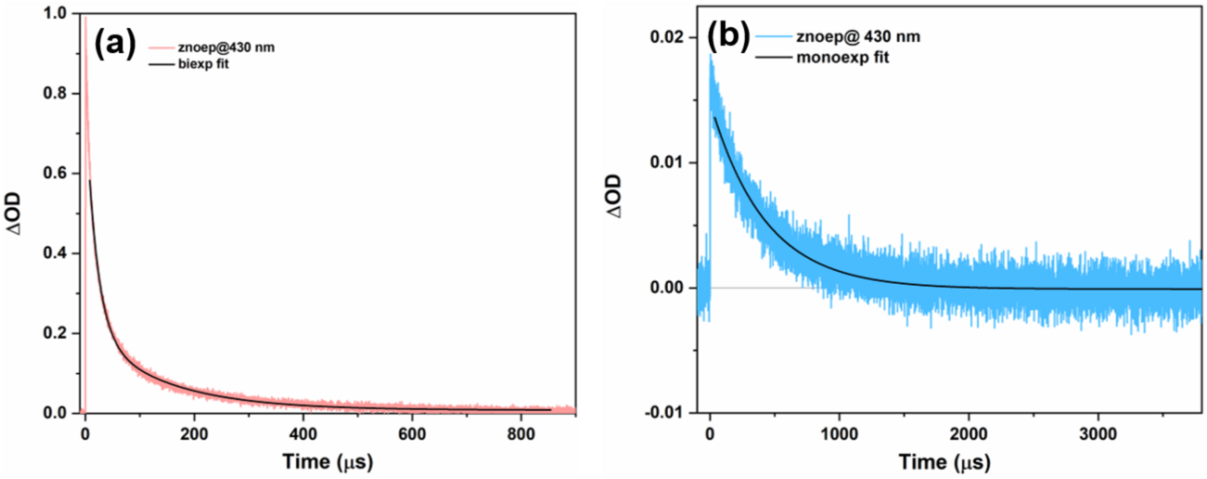

Figure S34: (a) Time trace of ZnOEP at 430 nm when excited with 6.5 mJ/pulse at 572 nm. (b) Time trace of ZnOEP at 430 nm when excited with 120 μJ/pulse at 572 nm.

***Table S5**. Fits to triplet decay of ZnOEP at different excitation powers, as shown in Figure S32.*

Power @ 572 nm	Type of exponential fit	Lifetimes (amplitude)
6 mJ/ pulse	Biexponential	20.21 μs (75%) & 141.52 μs (25%)
120 μJ/pulse	Monoexponential	424 μs

8.1 Determination of the triplet extinction coefficient:

For the measurement of the triplet extinction coefficient, the samples were excited at 570 nm. The concentration of the donor (ZnOEP) was 5 μM and the acceptor, **Tcdimer** was 20 μM. The extinction coefficient of the triplet excited state of the dimer was determined by using the energy transfer (EnT) method.[1] It can be described by the following equations:

$^3D^* \rightarrow D$ Intersystem crossing of the donor: $\tau_{0,D} = {}^{1}/_{k_{0,D}}$

$^3D^* + A \rightarrow D + {}^3A^*$ Energy transfer from donor to acceptor: k_{EnT}

$^3A^* \rightarrow A$ Intersystem crossing of acceptor: $\tau_A = {}^{1}/_{k_A}$

Here, D is the energy donor which is ZnOEP and A is the acceptor which is **Tcdimer**. The energy transfer efficiency and related equations are:

$$\phi_{EnT} = {}^{k_{EnT}[A]}/_{(k_{EnT}[A] + k_{0,D})} \text{……………(S1)}$$

$$\varepsilon_A = {}^{\varepsilon_D \times \Delta Abs_A}/_{\Delta Abs_D \times \phi_{EnT}} \text{………………(S2)}$$

The molar extinction coefficient of ZnOEP at 570 nm was taken as 37,716 $M^{-1}cm^{-1}$ as obtained from https://omlc.org/spectra/PhotochemCAD/html/050.html. There is a non-zero molar extinction coefficient (6,199 $M^{-1}cm^{-1}$) around 545-550 nm but, at 500 ns, the delta absorbance of the donor at 545 nm was found to be zero because of the overlapping ESA. So, we took a value of 37,716 – 6,199 = 31,517 $M^{-1}cm^{-1}$ as the value of the molar extinction coefficient for the donor.

From the plots Figure S35(a) and (b) below the following rate constants were obtained:

k_A= 2,774 s^{-1} $k_{0,D}$= 3,060.81 s^{-1} $k_{EnT}[A]$= 16,436.16 s^{-1}

From the spectra Figure S35 (c) the ΔAbs values of the donor and **Tcdimer** were obtained at 500 ns and 100 µs respectively.

ΔAbs_D= 0.01 at 570 nm

ΔAbs_A= 0.0042 at 525 nm

Substituting these values into equations (S1) and (S2), we get, ϕ_{EnT}= 0.843 and ε_A at 525 nm to be 15,700 $M^{-1}cm^{-1}$.

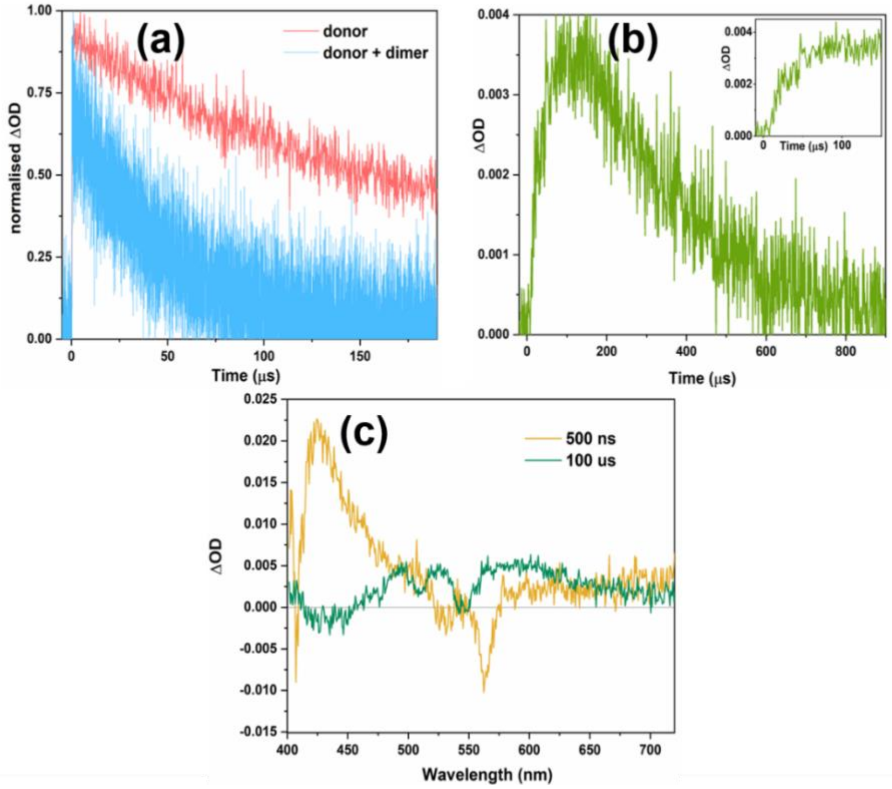

*Figure S35: (a) Time profile of the donor (ZnOEP) and that of the donor and **Tcdimer** mixture at 460 nm (b) Time profile of the donor and **Tcdimer** mixture at 525 nm with the inset showing the rise in few microseconds (c) Spectra of the donor and **Tcdimer** mixture: At 500 ns, the spectra mostly consist of the triplet state of ZnOEP and at 100 µs, the spectra mostly consist of the triplet state of **Tcdimer**.*

8.2 Determination of the triplet yield in Tcdimer:

Above we have determined the ε_T to be 15,700 $M^{-1}cm^{-1}$ at 525 nm. We have used three different methods to calculate the triplet yield using both the nsTA and fsTA setups.

8.2.1 Method I:

The spectral deconvolution method[9] was used to calculate the triplet quantum yield of **Tcdimer** from the fsTA data in toluene. The spectra at timepoints (0.2-0.5 ps) and (200-300 ps) were chosen and assigned to the first excited singlet state (S_1) and the correlated triplet pair state (TT), Figure S36. Then the basis spectra for S_1 and TT were obtained by adding the above spectra to a scaled ground state absorption spectrum, respectively, till no peaks of the absorption spectrum can be seen. The basis spectrum of the ground state bleach (GSB) was taken to be the absorption spectrum scaled to a similar absorbance as of the delta absorbances in the fsTA data. These basis spectra (GSB, S_1 and TT) were then normalised to the GSB that they share (i.e. divided by the peak absorption of the ground state absorption spectra used), and then a linear regression was done on the experimental TA data using MATLAB to yield the time dependent populations associated with each basis spectrum that best reproduces the TA data.

We see a quantitative 1:1 conversion from the S_1 to the TT state as shown below (90-120 % TT yield), Figure S36e. Similar analysis was extended to the nsTA data. Spectra at 30 ns was assigned to the TT state and the spectra at 200 ns to the T_1 state, Figure S36b. The rest of the analysis is similar to the one carried out above. Here we see that the free triplet yield lies between 95-145 %, Figure S36f.

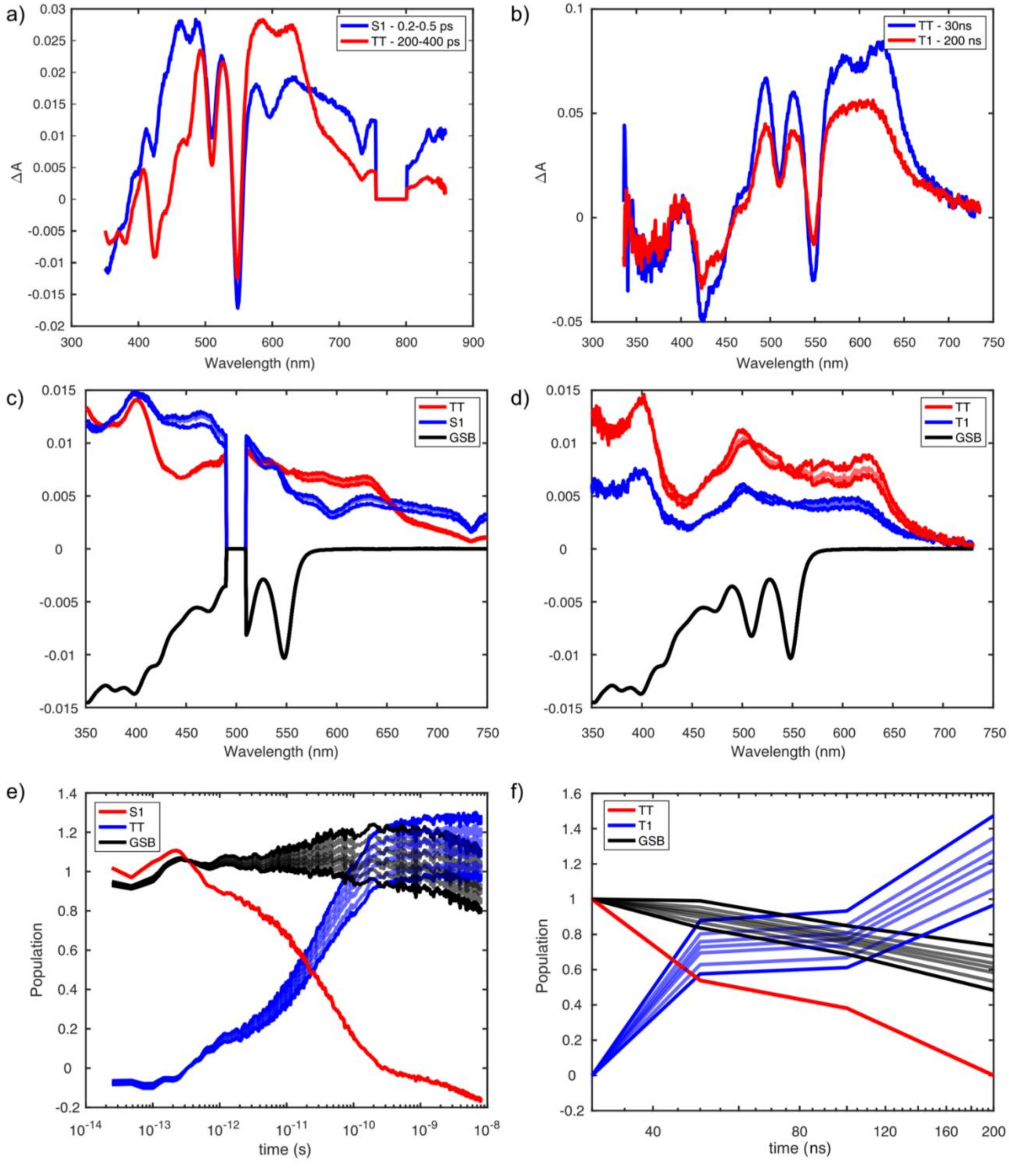

Figure S36. (a) fsTA spectra used to represent the S1 and TT TA spectra of ***Tcdimer****. (b) nsTA spectra used to represent the TT and T1 TA spectra. (c) Obtained basis spectra for S1, TT and GSB on the fs time scale. (d) Obtained basis spectra for TT, T1 and GSB on the ns time scale. (e) Extracted population dynamics from spectral deconvolution of fsTA data using the basis spectra in (c). (f) extracted population dynamics from spectral deconvolution of nsTA data using the basis spectra in (d).*

8.2.2 Method II:

Using ZnTPP as the reference because of its high triplet quantum yield (90%) and large triplet molar absorption coefficient (78,000 $M^{-1}cm^{-1}$ at 470 nm),[10] the triplet quantum yield of the dimer was calculated according to the following equation (S3).

These nsTA measurements were carried out in 1cm×1cm cuvette in toluene as the solvent. The samples were excited at 545 nm at very low powers of 95 µJ/pulse so as to avoid the effects of the annihilation of the triplet excited states of the ZnTPP. The nsTA spectra were measured at a delay of 700 ns.

$$\phi_{T,dimer} = \frac{\Delta Abs_{dimer}}{\Delta Abs_{ZnTPP}} \times \frac{\varepsilon_{T,ZnTPP}}{\varepsilon_{T,dimer}} \times \frac{1-10\^{}(-Abs_{545nm,ZnTPP})}{1-10\^{}(-Abs_{545nm,dimer})} \times \phi_{T,ZnTPP} \ldots\ldots\ldots\ldots (S3)$$

Here, ϕ_T, ε_T and ΔAbs and Abs stand for the quantum yield of the excited triplet states formed, the molar absorption coefficient of the triplet state, delta absorbance in nsTA at 700 ns and absorbance at 545 nm in the steady state measurement respectively. The values (summarized in Table S6) were obtained from the plots below (Figure S37 (a) and (b)) and substituted in equation (S3) to give the triplet quantum yield of **Tcdimer**. The triplet quantum yield of the dimer at 700 ns was determined to be $76 \pm 17\%$.

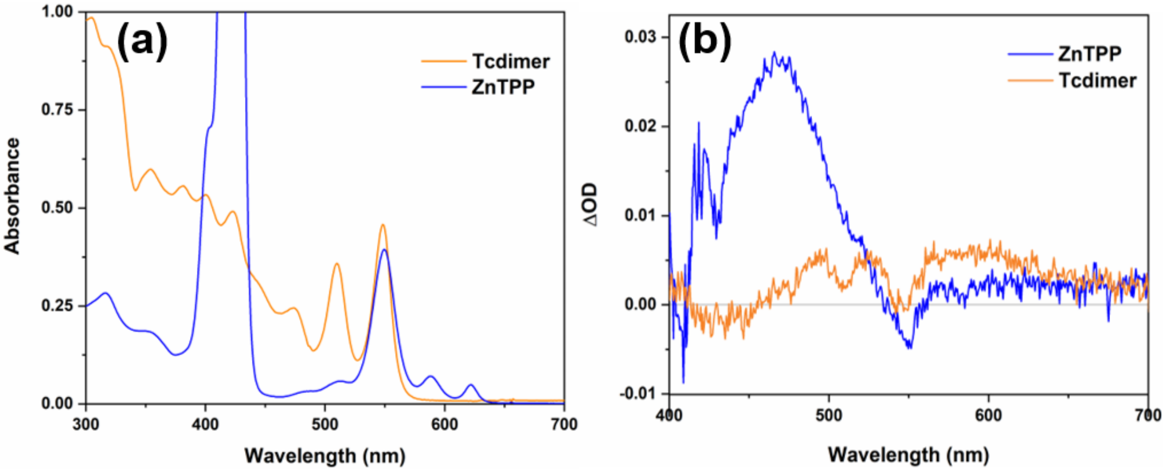

*Figure S37: (a) Absorbance spectra of **Tcdimer** and ZnTPP in toluene (b) nsTA spectra of the above at 700 ns.*

***Table S6**: Table summarizing the values used for determining the triplet yield of **Tcdimer in toluene**.*

	ΔAbs at 700 ns	Abs at 545 nm	ε_T(M^{-1}cm^{-1})	ϕ_T	$\Delta(\Delta Abs)$	$\Delta\varepsilon_T$(M^{-1}cm^{-1})
ZnTPP	0.027 (470 nm)	0.35	78000	0.90	0.0007	8000
Tcdimer	0.005 (525 nm)	0.4	15700	0.72	0.0009	1613

Error calculation:

The error associated with the triplet yield calculation is described in equation (S4).

$$\frac{\Delta\phi_{T,dimer}}{\phi_{T,dimer}} = \sqrt{\left(\frac{\Delta(\Delta Abs_{dimer})}{\Delta Abs_{dimer}}\right)^2 + \left(\frac{\Delta(\Delta Abs_{ZnTPP})}{\Delta Abs_{ZnTPP}}\right)^2 + \left(\frac{\Delta(\varepsilon_{T,ZnTPP})}{\varepsilon_{T,ZnTPP}}\right)^2 + \left(\frac{\Delta(\varepsilon_{T,dimer})}{\varepsilon_{T,dimer}}\right)^2} \ldots\ldots\ldots\ldots (S4)$$

Considering the large uncertainty of the relative measurements, we believe the triplet yields are closer to 100% in the **Tcdimer**, as estimated by Method I.

Similarly, free triplet yield of Tcdimer in PhCN was calculated using the values provided in table S7 at 700 ns and the error calculated as per equation S4 to yield a value of $33.7 \pm 8.2\%$. The excitation wavelength was 550 nm with a power of 105 µJ/pulse. The plots used to get the values in table S7 are provided in Figure S38.

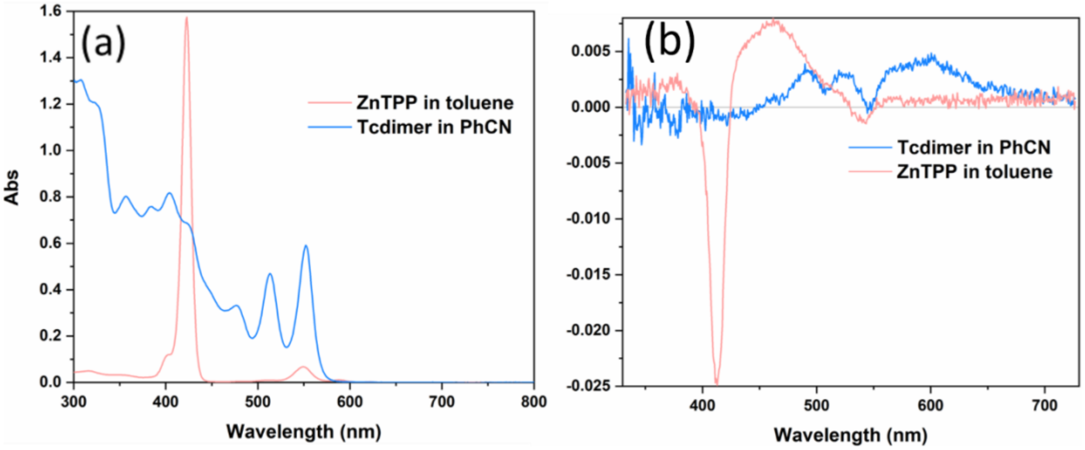

*Figure S38: (a) Absorbance spectra of **Tcdimer in PhCN** and ZnTPP in toluene (b) nsTA spectra of the above at 700 ns.*

***Table S7**: Table summarizing the values used for determining the triplet yield of **Tcdimer in PhCN**.*

	ΔAbs at 700 ns	Abs at 550 nm	ε_T(M^{-1}cm^{-1})	ϕ_T	$\Delta(\Delta Abs)$	$\Delta\varepsilon_T$(M^{-1}cm^{-1})
ZnTPP	0.0067(470 nm)	0.066	78000	0.90	0.0005	8000
Tcdimer	0.0026(525 nm)	0.562	15700	0.32	0.0005	1613

8.2.3 Method III:

Alternatively, we measured the spot size of the beam in the fsTA setup and used it to calculate the concentration of the excited singlet states formed. The fraction of light intensity of the pump laser (excitation wavelength: 500 nm) passing through a solution (Tcdimer in toluene) in a cuvette can be calculated as follows:

$$\frac{I}{I_0} = 1 - 10^{-A(500\,nm)} = 1 - 10^{-0.32} = 1 - 0.48 = 0.52$$

To calculate photons/pulse, the laser power is divided by the energy per photon and half the repetition rate of the laser. The area of the pump pulse was determined with the help of a beam profiler which measured the major and minor diameters of the ellipse-shaped pulse (Figure S39), and the cuvette length was 1 mm.

We used two different methods to determine the radii of the spot size as the laser spot was not perfectly circular. In the first method, the entire overlapped region comprising the two major spots and the probe was considered as a single spot and then the radii were determined. In the second method, the above-mentioned spot was divided into two spots and then the radii were calculated by summing the radii of the two individual spots. So, we have then two volumes and hence, two singlet and triplet yields.

Power = 1 mW Energy per photon = 3.975 × 10^{-19} J Repetition rate of the laser = 3 kHz

$$\frac{photons}{pulse} = \frac{laser\ power}{(energy\ per\ photon \times 0.5 \times repetition\ rate\ of laser)} = \frac{(1 \times 10^{-3} Js^{-1})}{3.975 \times 10^{-19} J \times 1.5 \times 10^{3} s^{-1}} = 1.67 \times 10^{12}$$

(i) Volume of the irradiated spot $V_1 = \pi \times r_1 \times r_2 \times d = 3.14 \times 192\ \mu m \times 246.5\ \mu m \times 0.1\ cm = 1.48 \times 10^{-7} L\ (one\ overlapped\ size)$

(ii) Volume of the irradiated spot $V_2 = \pi \times r_1 \times r_2 \times d = 3.14 \times 241.6\ \mu m \times 232.5\ \mu m \times 0.1\ cm = 1.76 \times 10^{-7} L\ (sum\ of\ two\ sizes)$

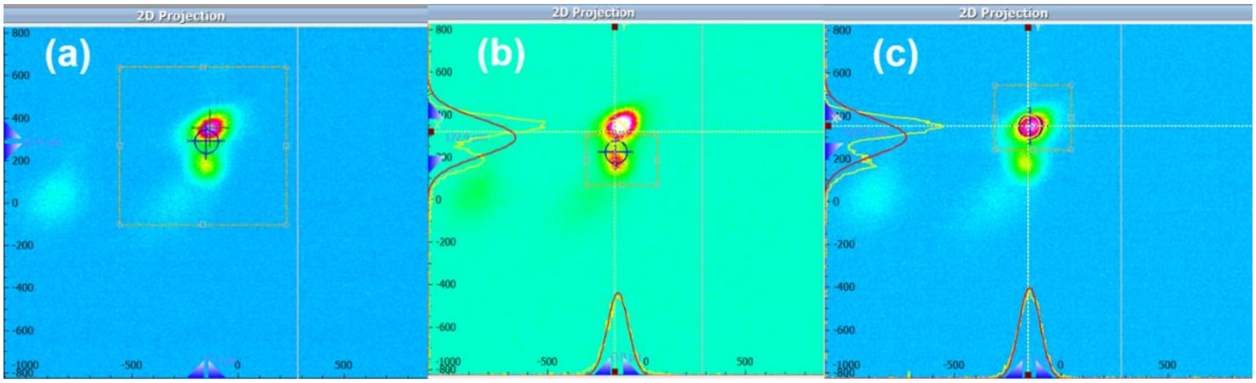

Figure S39: (a) Beam profile of the 500 nm pump (b), (c) Dividing the area in (a) in two sections

The concentration of the singlet excited states can then be calculated as follows:

$$[S_1^1] = \frac{(\frac{photons}{pulse}) \times \frac{I}{I_0}}{N_A \times V_1} = \frac{(1.67 \times 10^{12} \times 0.52)}{(1.48 \times 10^{-7} L \times 6.02 \times 10^{23} mol^{-1})} = 9.74 \times 10^{-6} M$$

$$[S_1^2] = \frac{(\frac{photons}{pulse}) \times \frac{I}{I_0}}{N_A \times V_2} = \frac{(1.67 \times 10^{12} \times 0.52)}{(1.76 \times 10^{-7} L \times 6.02 \times 10^{23} mol^{-1})} = 8.19 \times 10^{-6} M$$

Using the triplet extinction coefficient the yield of the total number of triplets at 8 ns is then calculated as follows:

$$\phi_{T1}^1 = \frac{(\frac{\Delta Abs}{\varepsilon_T \times d})}{[S_1^1]} = \frac{\frac{(0.018)}{(15700 \times 0.1)}}{9.74 \times 10^{-6}} = 1.17$$

$$\phi_{T1}^2 = \frac{(\frac{\Delta Abs}{\varepsilon_T \times d})}{[S_1^2]} = \frac{\frac{(0.018)}{(15700 \times 0.1)}}{8.19 \times 10^{-6}} = 1.39$$

Considering that the triplets at 8 ns correspond to triplet-pairs, and that by 200 ns the signal is halved, the final yield of free triplets is half of the above calculated values, around 70%, similar to that estimated in Method II. However, the estimate in method III assumes that there is no change in the extinction coefficient of a triplet in the triplet pair compared to a free triplet. This assumption might not be fully accurate, and considering the general uncertainty in the extinction coefficients, we make a combined assessment using all determined yields (methods I-III) and find it likely that the yields lie in the interval 70-100 %.

9. Calculations of driving force for electron transfer

9.1 Oxidative quenching:

The reduction potentials of **TCAQ** (**TCAQ/TCAQ•⁻** and **TCAQ•⁻/TCAQ⁻**) obtained from its CV (Figure S22) are -0.96 V and -1.84 V vs Fc^+/Fc respectively. For **Tcdimer**, the reduction and oxidation potential (vs Fc^+/Fc) is estimated to be -1.64 V and 0.77 V respectively from its CV (Figure S23). These potentials are also similar to what has been reported for **Tc-BP-Tc**.[1] So, we can conclude that the driving force calculations should apply for **Tc-BP-Tc** as well.

Considering that the ^{1}TT state energy is similar to the S_1 state, we assume that it is roughly equal to 2.25 eV, based on the intersection point of the normalized absorption and fluorescence spectra (Figure S20). We can then calculate the oxidation potential of the ^{1}TT state using the below formula:

$$Eox_{TT}^* = Eox_{dimer} - E_{0,0}/e$$

$$Eox_{TT}^* = 0.77\,V - 2.25\,V = -1.48\,V$$

The oxidation potential of the triplet state of the dimer can be calculated if we assume the triplet state energy to be around 1.25 eV.[11,12] Then we get,

$$Eox_{Triplet}^* = Eox_{dimer} - E_{0,0}/e$$

$$Eox^*_{Triplet} = 0.77\ V - 1.25\ V = \ -0.48\ V$$

The driving force for electron transfer from the ^{1}TT state of the dimer to **TCAQ** can be calculated as per the following:[13]

$$\Delta G = -e(Ered_{TCAQ/TCAQ^{\bullet-}} - Eox^*_{TT})$$

$$\Delta G = -e(-0.96V - (-1.48V))$$

$$\Delta G = -0.52\ eV$$

The negative Gibbs free energy change suggests that the electron transfer from the TT state of the dimer to the ground state of **TCAQ** to yield **TCAQ**$^{\bullet-}$ is feasible.

The driving force for electron transfer from the triplet state of the dimer to **TCAQ** can be calculated as per the following:

$$\Delta G = -e(Ered_{TCAQ/TCAQ^{\bullet-}} - Eox^*_{Triplet})$$

$$\Delta G = -e(-0.96V - (-0.48V))$$

$$\Delta G = 0.48\ eV$$

The positive Gibbs free energy change suggests that the electron transfer from the triplet state of the dimer to the ground state of **TCAQ** to yield **TCAQ**$^{\bullet-}$ is not feasible.

9.2 Reductive quenching:

The oxidation potential of **DIPEA/DIPEA**$^{\bullet-}$ in THF is 0.93 V vs SCE.[14] Converting it with respect to Fc^+/Fc, we get the oxidation potential to be 0.53 V.

The reduction potential of the ^{1}TT state of the dimer can be calculated as below.

$$Ered^*_{TT} = Ered_{dimer} + E_{0,0}/e$$

$$Ered^*_{TT} = -1.64\ V + 2.25\ V = 0.61\ V$$

The reduction potential of the triplet state of the dimer can be calculated as below.

$$Ered^*_{Triplet} = Ered_{dimer} + E_{0,0}/e$$

$$Ered^*_{TT} = -1.64\ V + 1.25\ V = -0.39\ V$$

The driving force for electron transfer from **DIPEA** to the TT state of the dimer can be calculated as per the following:

$$\Delta G = -e(Ered^*_{TT} - Eox_{DIPEA/DIPEA^{\bullet-}})$$

$$\Delta G = -e(0.61V - 0.53\ V)$$

$$\Delta G = -0.08\ eV$$

The negative Gibbs free energy change suggests that the electron transfer from **DIPEA** to the TT state of the dimer is feasible.

The driving force for electron transfer from **DIPEA** to the triplet state of the dimer can be calculated as per the following:

$$\Delta G = -e(Ered^*_{Triplet} - Eox_{DIPEA/DIPEA^{\bullet-}})$$

$$\Delta G = -e(-0.39V - 0.53\ V)$$

$$\Delta G = 0.92\ eV$$

The positive Gibbs free energy change suggests that the electron transfer from **DIPEA** to the triplet state of the dimer is feasible.

Similarly, the driving force of the other electron donors and acceptors can be calculated. They are summarized below in Table S8. (For **TTF**, the potentials in DCM are taken from Martín.[15] For Chloranil, in DCM, the potential is taken from Nakamura *et al.*[1] For **4F-TCNQ** in DCM, the potentials are taken from Kivala et al.[16]

Table S8*: Table having values of reduction potentials, oxidation potentials (vs Fc^+/Fc) of the dimer and involved quenchers, and driving force for electron transfer.*

Eox = 0.77 V<br>Ered = -1.64 V<br>(**Tcdimer**)	Eox	Ered	Ered*(triplet)<br>Or<br>Eox*(triplet)	Ered*(TT)<br>Or<br>Eox*(TT)	ΔG(triplet, substrate)	ΔG(TT, substrate)
**DIPEA**	0.53V	-	-0.39V	0.61V	0.92 eV	-0.08eV
**TTF**	-0.03V,<br>0.27V	-	-0.39V	0.61 V	0.36eV,0.66eV	-0.64eV,-0.34eV
**Chloranil**	-	-0.36V	-0.48 V	-1.48 V	-0.12eV	-1.12eV
**TCAQ**	-	-0.96 V,-1.84V	-0.48 V	-1.48 V	0.48eV,1.36eV	-0.52eV,0.36eV
**4F-TCNQ**	-	0.19 V,-0.48 V	-0.48 V	-1.48 V	-0.67eV,0.0 eV	-1.67eV,-0.98 eV

10. Additional quenching experiments

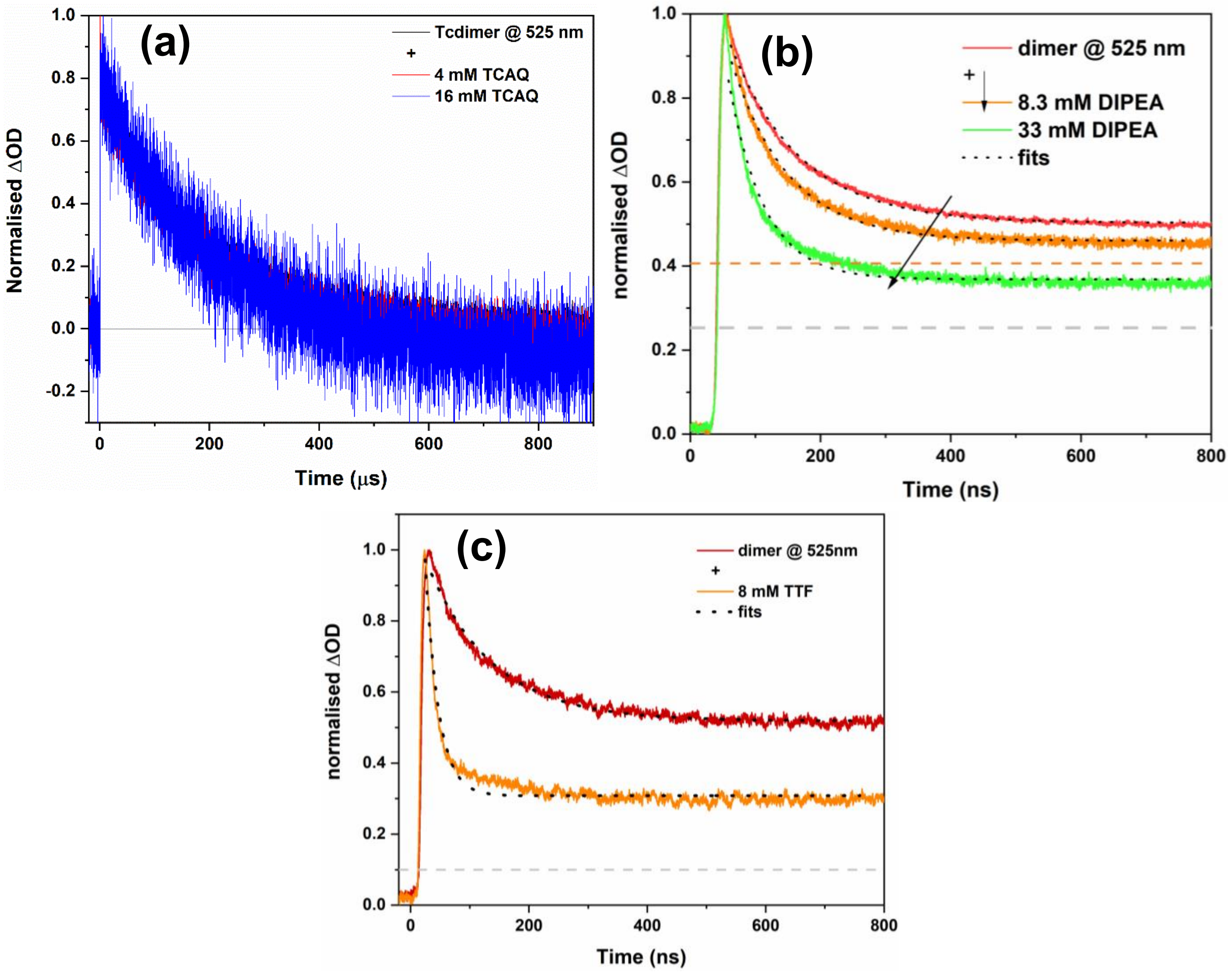

*Figure S40: (a) Time profiles at 525 nm when **Tcdimer is excited at** 545 **nm** in THF upon adding increasing concentrations of **TCAQ**, showing no quenching of the triplet lifetime. (b) Time profiles at 525 nm of **Tcdimer** upon adding increasing concentrations of **DIPEA**, showing quenching of the TT state.(c)Time profiles at 525 nm of*

***Tcdimer** upon adding 8mM TTF. The black dotted lines represent the fits and the colored dashed lines represent the expected intensity if we assume the TT state acts as the singlet without any recombination.*

The amount of quenching of the TT state is estimated from the reduction in the first time component in the decay at 525 nm, which monitors the TT and T_1 excited state absorption. Without quencher the TT decay from 100% signal to 50 % occurs with a time constant of 107 ns in THF. With 4 mM TCAQ this decay is reduced to 66 ns, yielding a quenching efficiency of: $1 - \tau_{TCAQ}/\tau_0 = 1 - 66/107 = 0.38 = 38\%$ (Table S9 and Figure 3a in main manuscript).

The remaining triplet signal if all quenched TT states are unable to form triplets would then originate from the 62% remaining TT population. As the T_1 signal is half as intense as the TT signal as we have concluded that 1 TT state results in only 1 T_1, the 62% TT remaining after quenching would yield a triplet population resulting in 31% of the original signal at 525 nm.

***Table S9**. Fitting parameters to TT decay profiles measured at 525 nm. * At these higher concentrations, the quenching efficiency calculated would be dynamic quenching efficiency as contributions from static quenching can exist.*

Samples in THF	TT Lifetimes	Quenching efficiency	Expected triplet intensity	Observed triplet intensity
Tcdimer	107 ns	-	-	50%
Tcdimer with 4 mM TCAQ	66 ns	38 %	31%	36%
Tcdimer with 16 mM TCAQ*	23 ns	80%	10%	25%
Tcdimer with 8 mM DIPEA	88 ns	17%	41.5%	46%
Tcdimer with 33 mM DIPEA	53 ns	50%	25%	36%
Tcdimer with 8 mM TTF*	23 ns	80%	10%	25%

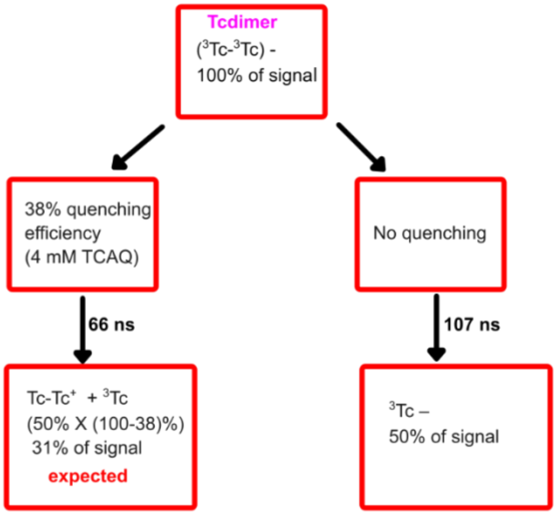

Figure S41: Flowchart showing the calculation for the expected signal intensity given in Table S9. Initially, if we start with 100% of signal at the TT state, without any quencher, we get 50% of signal after 100 ns corresponding to one free triplet. In the presence of quencher, 4mM TCAQ, the lifetime gets quenched to 66 ns, based on which we get 38% as the quenching efficiency. We can then calculate the expected triplet intensity based on the unquenched TT population (100 -38) % which gives 31% as the Tc-Tc$^+$ does not contribute to the signal.

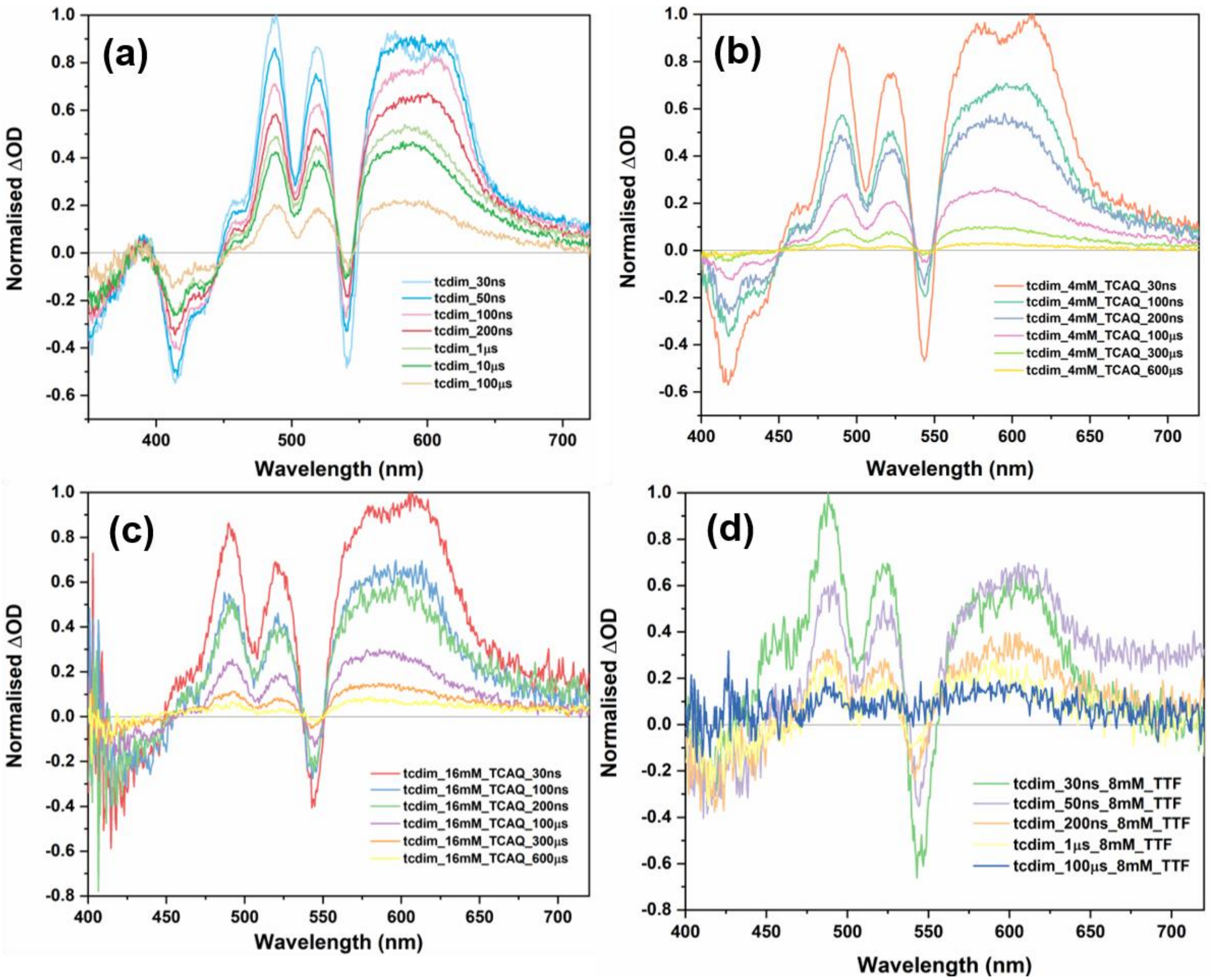

Figure S42: (a) nsTA spectra of ***Tcdimer*** *in THF when excited at 545 nm (b) nsTA spectra of* ***Tcdimer*** *in THF with 4mM* ***TCAQ*** *when excited at 545 nm (c) nsTA spectra of* ***Tcdimer*** *in THF with 16 mM* ***TCAQ*** *when excited at 545 nm (d) nsTA spectra of* ***Tcdimer*** *in THF with 8 mM* ***TTF*** *when excited at 545 nm*

As reference, the quenching of **TIPS-Tc** and **Tcmonomer** S_1 by **TCAQ** and **TTF** was also investigated, shown in Figure S42-S45. The fluorescence quenching in TCSPC experiments was used to verify quenching of the S_1 state. The relative triplet yield was determined from the amplitude of the long-lived triplet signal in nsTA measurements.

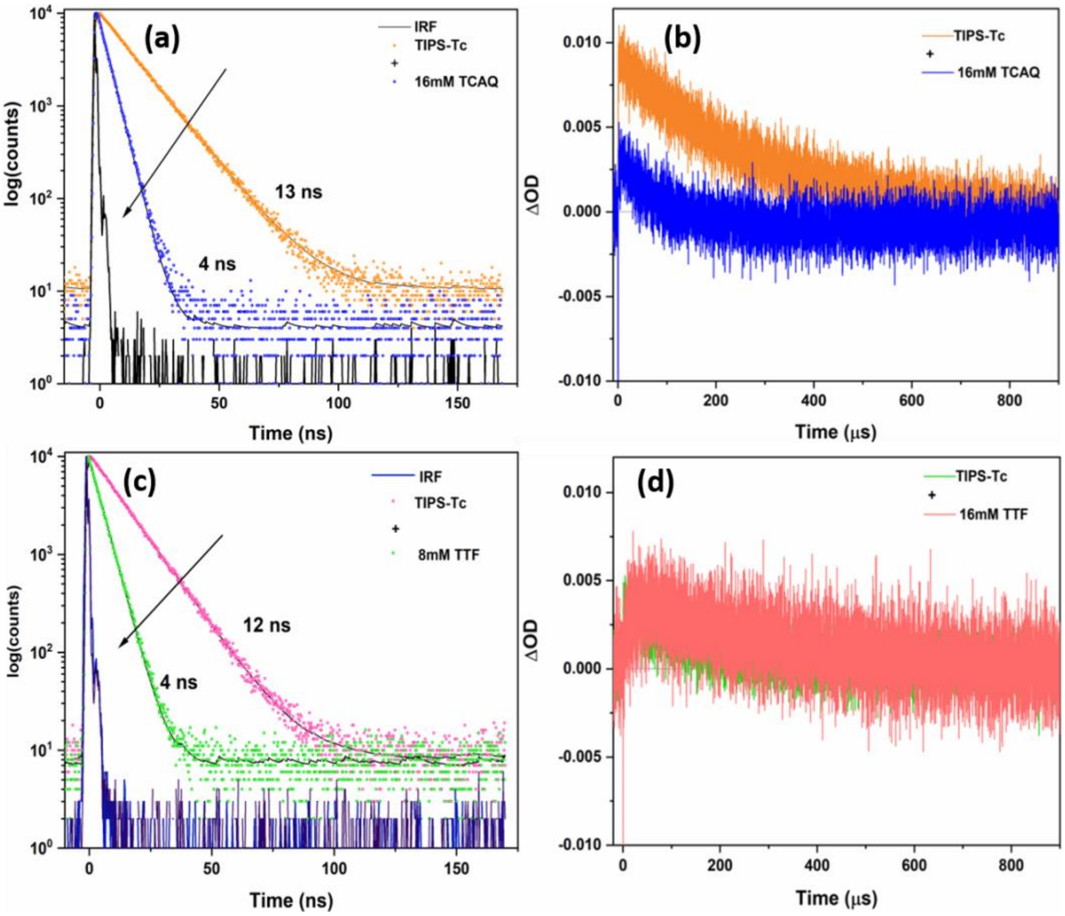

*Figure S43: (a) Fluorescence decays of **TIPS-Tc** at 550 nm upon addition of 16 mM **TCAQ** in THF. (b) Time profiles of delta absorbance at 502 nm of **TIPS-Tc** upon addition of 16 mM **TCAQ** in THF. (c) Fluorescence decays of **TIPS-Tc** at 550 nm upon addition of 8 mM **TTF** in toluene. (d) Time profiles of delta absorbance at 502 of **TIPS-Tc** upon addition of 8 mM **TTF** in toluene.*

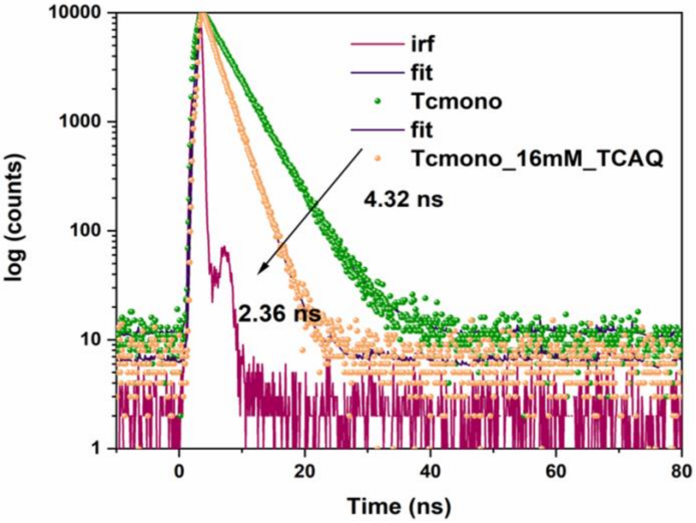

*Figure S44: TCSPC decay at 546 nm of **Tcmonomer** (when excited at 470 nm) on adding 16 mM **TCAQ** in THF. The lifetime (monoexponential fit) gets quenched from 4.2 ns to 2.4 ns upon adding 16 mM **TCAQ**.*

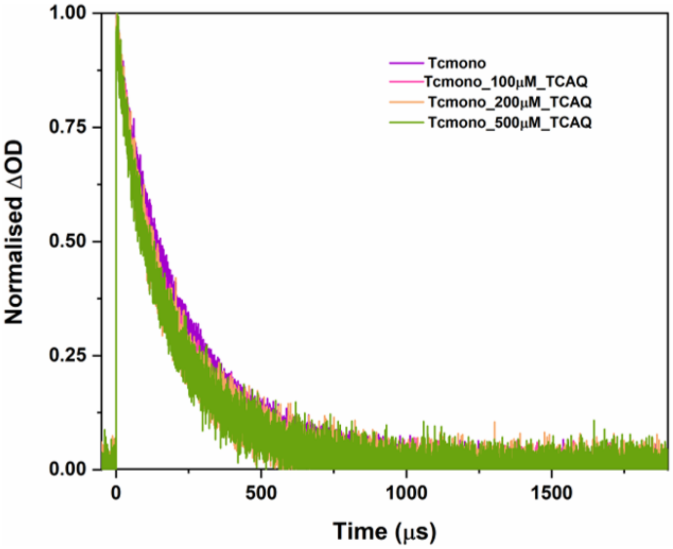

Figure S45: The time trace of ***Tcmonomer*** *at 516 nm upon adding increasing concentrations of* ***TCAQ*** *in THF showing negligible quenching of the triplet state.*

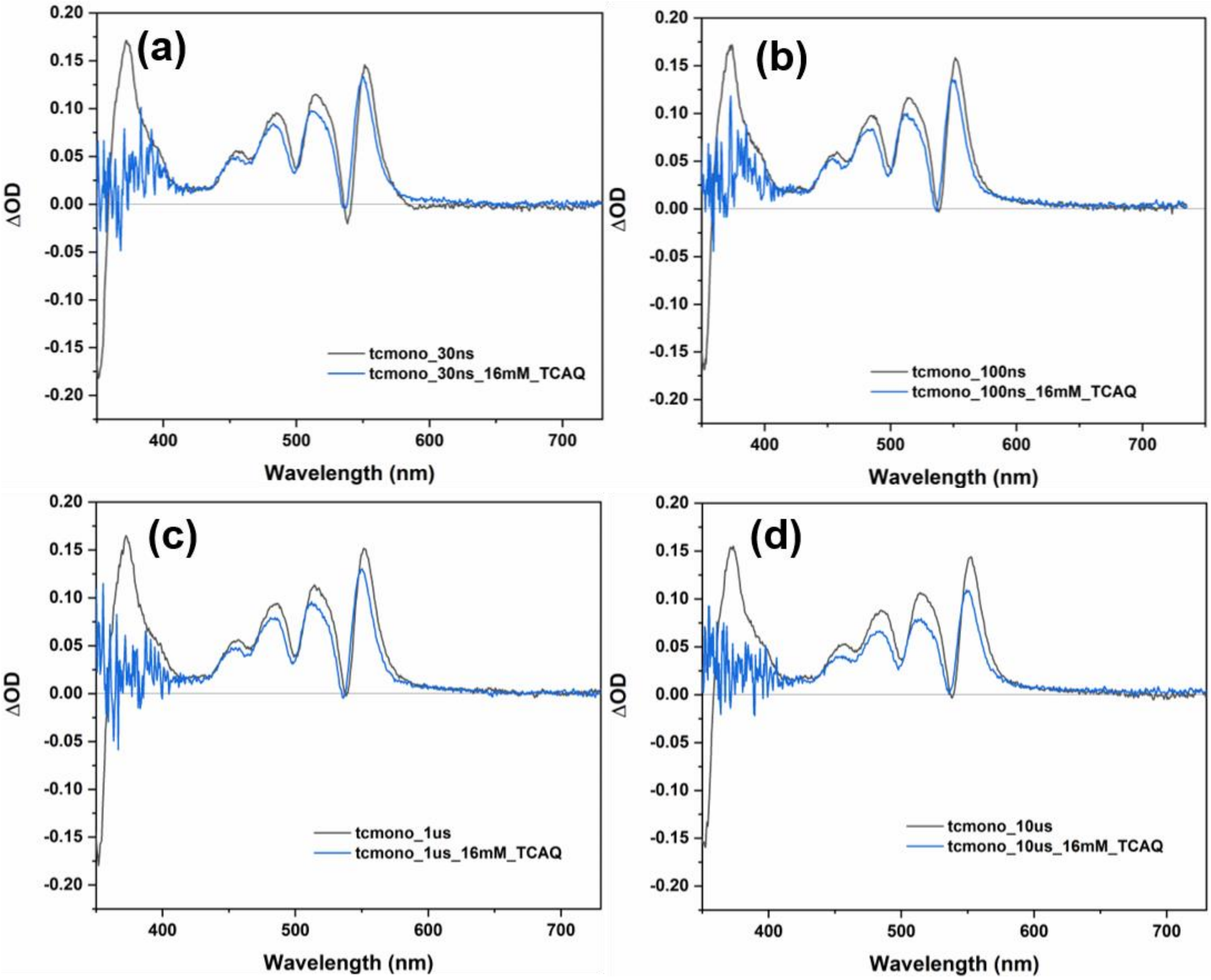

Figure S46: Spectra of ***Tcmonomer*** *at different time intervals comparing the spectra with (blue) and without (black) 16 mM* ***TCAQ****. The samples were excited at 542 nm in THF.*

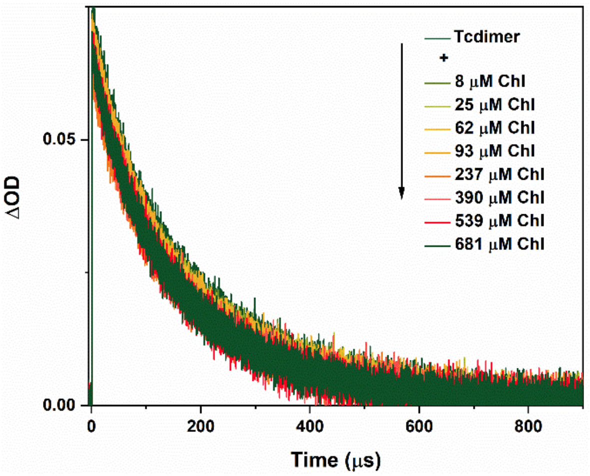

*Figure S47: The time trace of **Tcdimer** at 600 nm upon adding increasing concentrations of **Chl** in THF, showing no quenching of the triplet.*

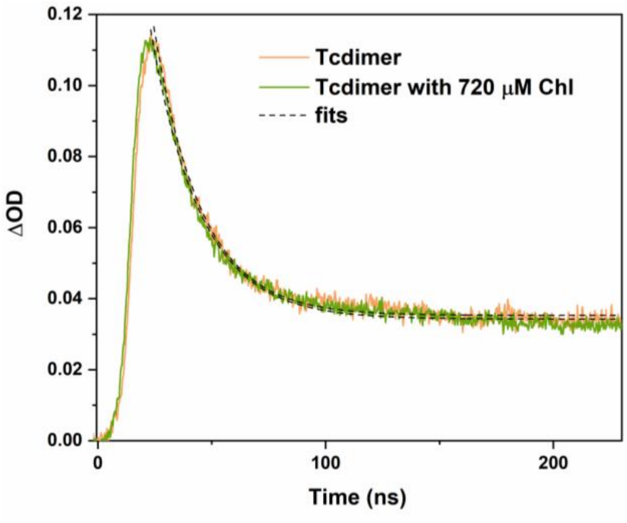

*Figure S48: The time trace of **Tcdimer** at 525 nm upon adding 720 uM concentration of **Chl** in PhCN, showing no quenching of the TT state.*

11. Determination of the electron transfer yield:

11.1 ET yield from Tcdimer to Chl

To determine the electron transfer yield from **Tcdimer** to chloranil (**Chl**), the following equation was used:

$$\phi_{ET} = \frac{\Delta Abs_{Chl-}}{\Delta Abs_{ZnTPP}} \times \frac{\varepsilon_{T,ZnTPP}}{\varepsilon_{Chl-}} \times \frac{1-10\text{^}(-Abs_{552nm,ZnTPP})}{1-10\text{^}(-Abs_{552nm,dimer})} \times \phi_{T,ZnTPP} \ldots\ldots (S5)$$

The experiment was done at low powers (~ 160 μJ/pulse) to avoid triplet triplet annihilation in ZnTPP. The molar extinction coefficient of reduced chloranil anion at 450 nm is 9700 $M^{-1}cm^{1}$.[17] The yield was calculated at a time delay of 10 μs using values listed in Tables S10 and S11.

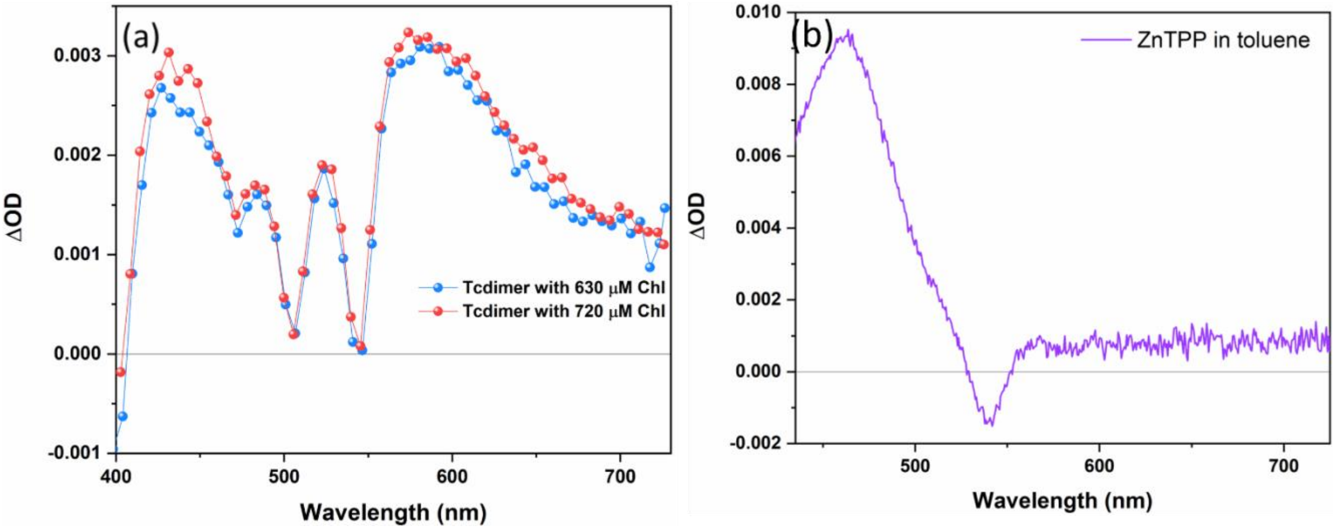

Figure S49:(a) Spectra of ***Tcdimer*** *with 630 μM and 720 μM concentrations of* ***Chl*** *in PhCN upon exciting at 552 nm at 10 μs (b) Spectra of ZnTPP in toluene upon exciting at 552 nm at 10 μs.*

Table S10*: Table summarizing the values used for determining the ET yield with* ***Tcdimer*** *at 630 μM* ***Chl****.*

	ΔAbs at 10 μs.	Abs at 552 nm	ε (M^{-1}cm^{-1})	ϕ_T	$\Delta(\Delta Abs)$	$\Delta\varepsilon$ (M^{-1}cm^{-1})
ZnTPP	0.0087 (470 nm)	0.052	78000	0.90	0.0003	8000
Chl anion	0.0022 (450 nm)	0.5 (**Tcdimer**)	9700	0.29	0.0004	990

Table S11: *Table summarizing the values used for determining the ET yield with* ***Tcdimer*** *at 720 μM* ***Chl****.*

	ΔAbs at 10 μs.	Abs at 552 nm	ε (M^{-1}cm^{-1})	ϕ_T	$\Delta(\Delta Abs)$	$\Delta\varepsilon$ (M^{-1}cm^{-1})
ZnTPP	0.0087 (470 nm)	0.052	78000	0.90	0.0003	8000
Chl anion	0.0026 (450 nm)	0.5 (**Tcdimer**)	9700	0.35	0.0004	990

The error in the ET yield was calculated using the following equation using values listed in the above table:

$$\frac{\Delta\phi_{ET}}{\phi_{ET}} = \sqrt{\left(\frac{\Delta(\Delta Abs_{Chl-})}{\Delta Abs_{Chl-}}\right)^2 + \left(\frac{\Delta(\Delta Abs_{ZnTPP})}{\Delta Abs_{ZnTPP}}\right)^2 + \left(\frac{\Delta(\varepsilon_{Chl-})}{\varepsilon_{Chl-}}\right)^2 + \left(\frac{\Delta(\varepsilon_{T,ZnTPP})}{\varepsilon_{T,ZnTPP}}\right)^2}$$

Hence, the ET yield obtained at 630 μM and 720 μM concentrations of **Chl** are 29 $\pm$ 6.8 % and 35 $\pm$ 7.1% respectively.

11.2 ET yield from Tc-BP-Tc to 4F-TCNQ

In a similar manner, the ET yield from **Tc-BP-Tc** triplet to **4F-TCNQ** at 2 μs was calculated using equation S5 and values listed in Table S12 and Table S13:

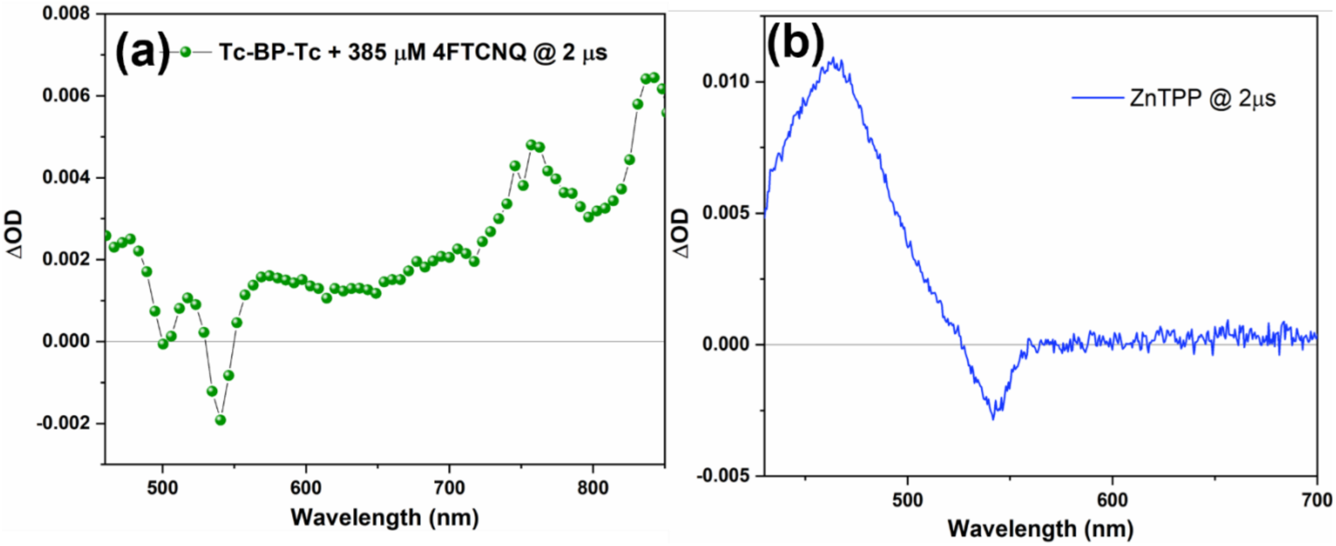

*Figure S50:(a) Spectra of **Tc-BP-Tc** with 385 μM concentration of **4F-TCNQ** in PhCN upon exciting at 548 nm at 2 μs (b) Spectra of ZnTPP in toluene upon exciting at 548 nm at 2 μs.*

Table S12: *Table summarizing the values used for determining the ET yield with **Tc-BP-Tc** at 385 μM **4F-TCNQ**.*

	ΔAbs at 2 μs.	$Abs\ at\ 548\ nm$	ε ($M^{-1}cm^{-1}$)	ϕ_T	$\Delta(\Delta Abs)$	$\Delta\varepsilon$ ($M^{-1}cm^{-1}$)
ZnTPP	0.01 (470 nm)	0.096	78000	0.90	0.0003	8000
4F-TCNQ anion	0.0044 (757 nm)	0.55 **(Tc-BP-Tc)**	19000	0.45	0.0008	1938

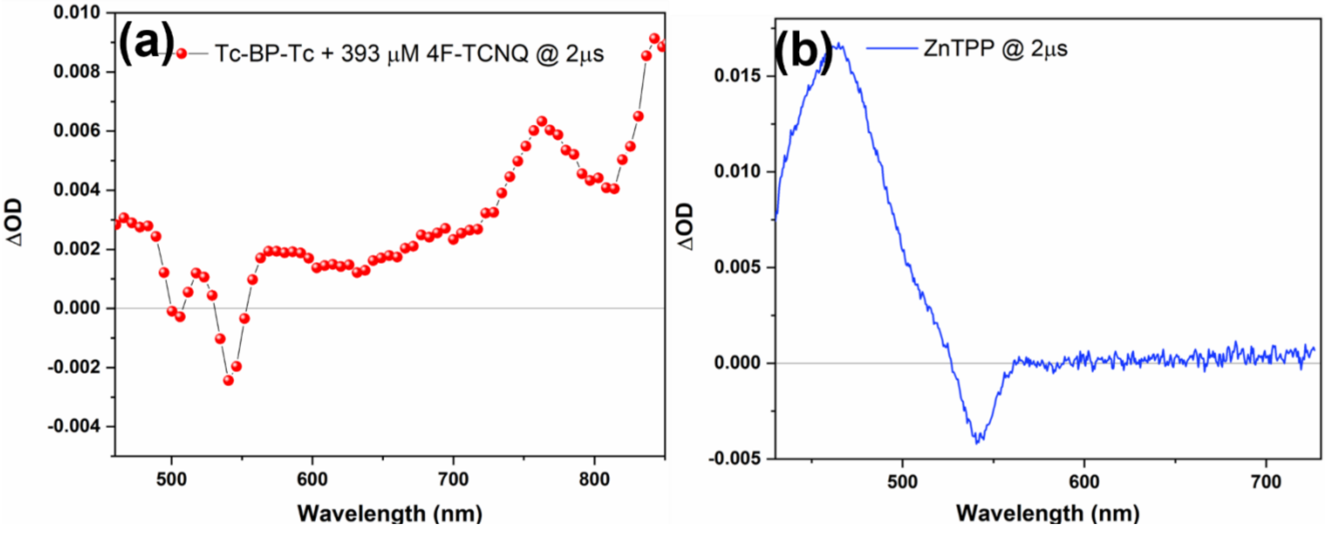

Figure S51:(a) Spectra of Tc-BP-Tc with 393 μM concentration of 4F-TCNQ in PhCN upon exciting at 548 nm at 2 μs (b) Spectra of ZnTPP in toluene upon exciting at 548 nm at 2 μs.

Table S13: *Table summarizing the values used for determining the ET yield with **Tc-BP-Tc** at 393 μM **4F-TCNQ**.*

	ΔAbs at 2 μs.	$Abs\ at\ 548\ nm$	ε ($M^{-1}cm^{-1}$)	ϕ_T	$\Delta(\Delta Abs)$	$\Delta\varepsilon$ ($M^{-1}cm^{-1}$)
ZnTPP	0.016 (470 nm)	0.13	78000	0.90	0.0003	8000
4F-TCNQ anion	0.0061 (757 nm)	0.67 **(Tc-BP-Tc)**	19000	0.47	0.0008	1938

The reported literature value of the molar extinction coefficient of the reduced **4F-TCNQ** anion at 757 nm was used.[18] The error is also calculated as in section 11.1, and we obtain electron transfer yields from **Tc-BP-Tc** to **4F-TCNQ** of 45 ± 10% and 47 ± 8% for **4F-TCNQ** concentrations of 385 µM and 393 µM, respectively.

Table S14. *Fitting parameters to TT decay profiles measured at 520 nm for **Tc-BP-Tc**.* At these higher concentrations the quenching efficiency calculated would be dynamic quenching efficiency as contributions from static quenching can exist.*

Samples in THF	TT Lifetimes	Quenching efficiency	Expected triplet intensity	Observed triplet intensity
Tc-BP-Tc	372 ns	-	-	36%
Tc-BP-Tc with 4 mM TCAQ	154 ns	58 %	15%	18%
Tc-BP-Tc with 16 mM TCAQ*	41 ns	89%	4%	8%

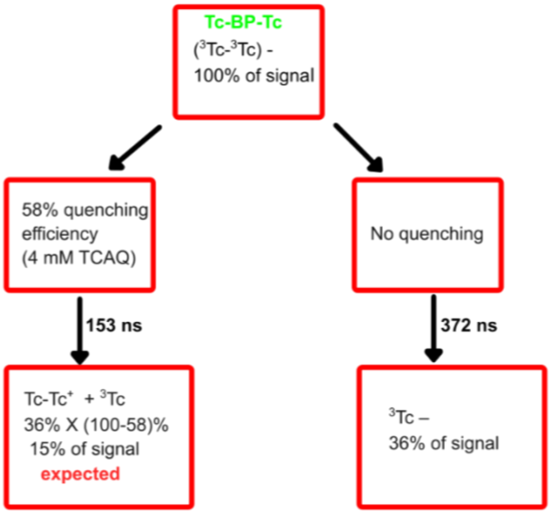

Figure S52: Flowchart showing the calculation for the expected signal intensity given in Table S14. Initially, if we start with 100% of signal at the TT state, without any quencher, we get 36% of signal after 100 ns corresponding to one free triplet. In the presence of quencher, 4mM TCAQ, the lifetime gets quenched to 153 ns, based on which we get 58% as the quenching efficiency. We can then calculate the expected triplet intensity based on the unquenched TT population (100 -58) % which gives 15% as the Tc-Tc⁺ does not contribute to the signal.

11.3 Collision rate calculations:

The collision rate was calculated as per the following equation:

$$k_T = k_T^0 + k_c[Q]$$

Where k_T represents decay of the triplet in the presence of the quencher, k_T^0 represents the decay of the unquenched triplet, k_c represents the collision rate and $[Q]$ is the concentration of the quencher.

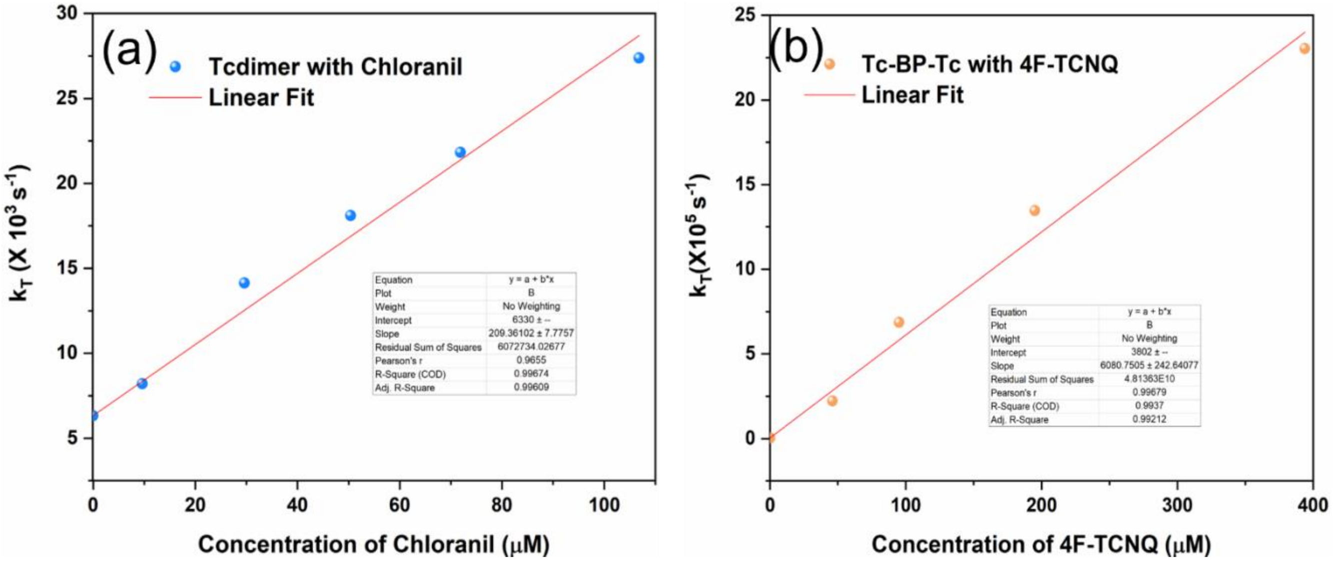

Figure S53: Triplet decay rates of (a) Tcdimer with Chloranil upto 100 μM, with the slope yielding a value of $2 \times 10^8 M^{-1}s^{-1}$ *as the collision rate. Beyond 100 μM, the decays were biexponential due to which a linear fit was not suitable. (b) Tc-BP-Tc with 4F-TCNQ wih the slope yielding a value of* $6 \times 10^9 M^{-1}s^{-1}$ *as the collision rate.*

12. Low Temperature nsTA experiments:

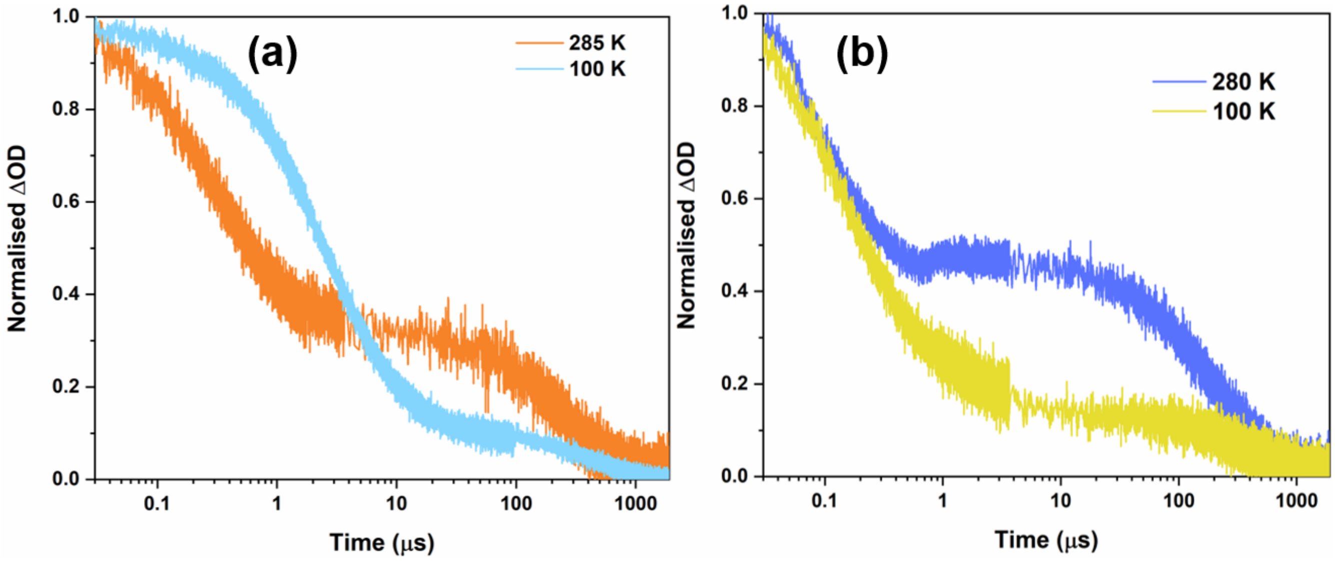

Figure S54:(a) The time profiles of the signal at 519 nm of ***Tc-BP-Tc*** *in MeTHF when excited at 543 nm. The initial decay (TT state) increases from 400 ns to 3.5 μs from 285 K to 100 K. (b) The time profiles of the signal at 525 nm of* ***Tcdimer*** *in MeTHF when excited at 545 nm. The initial decay (TT state) increases from 110 ns to 350 ns from 280 K to 100 K.*

13 Additional tr-EPR data:

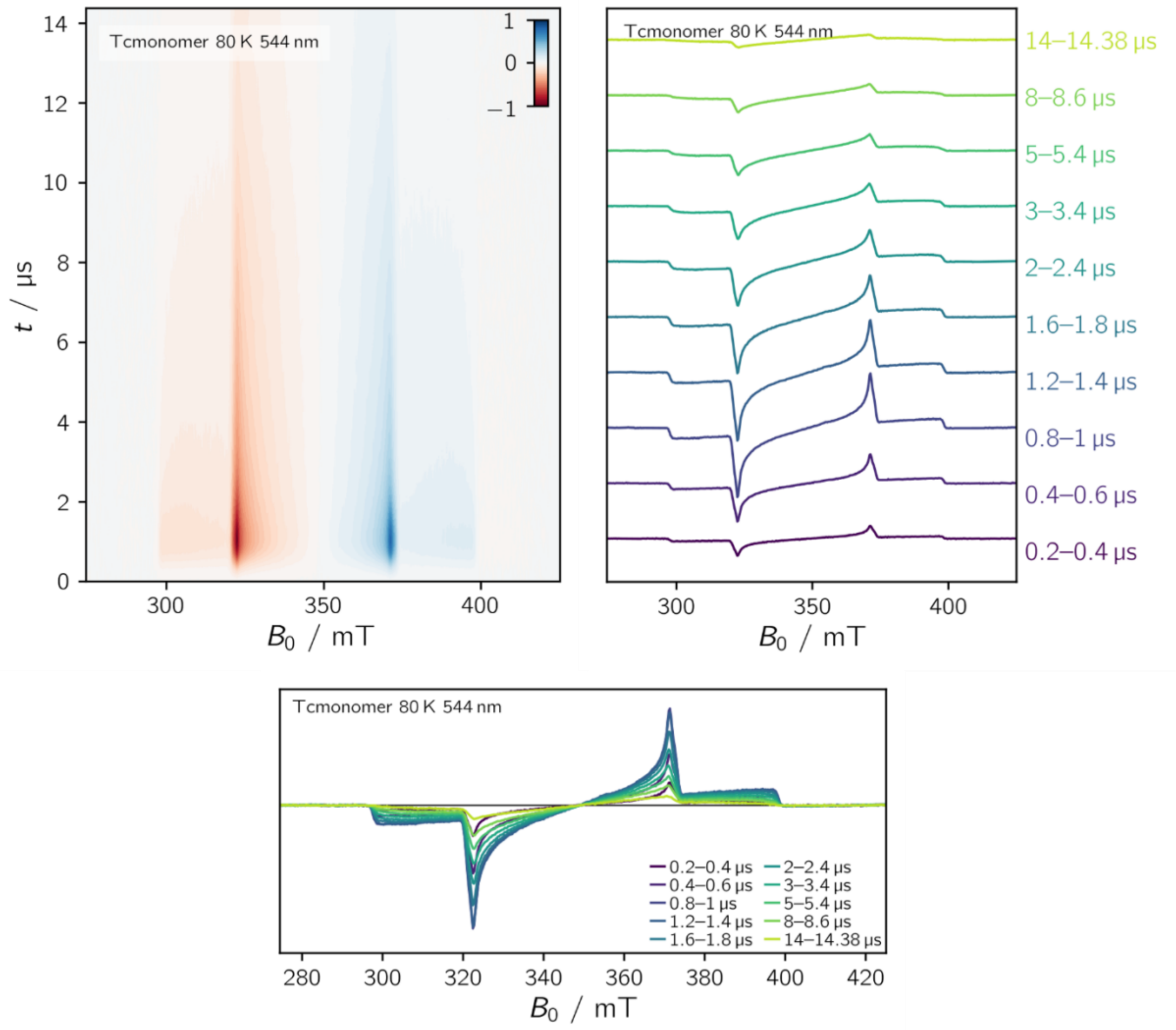

Figure S55: Time-dependent evolution of the tr-EPR spectra of ***Tcmonomer*** *recorded at 80 K.*

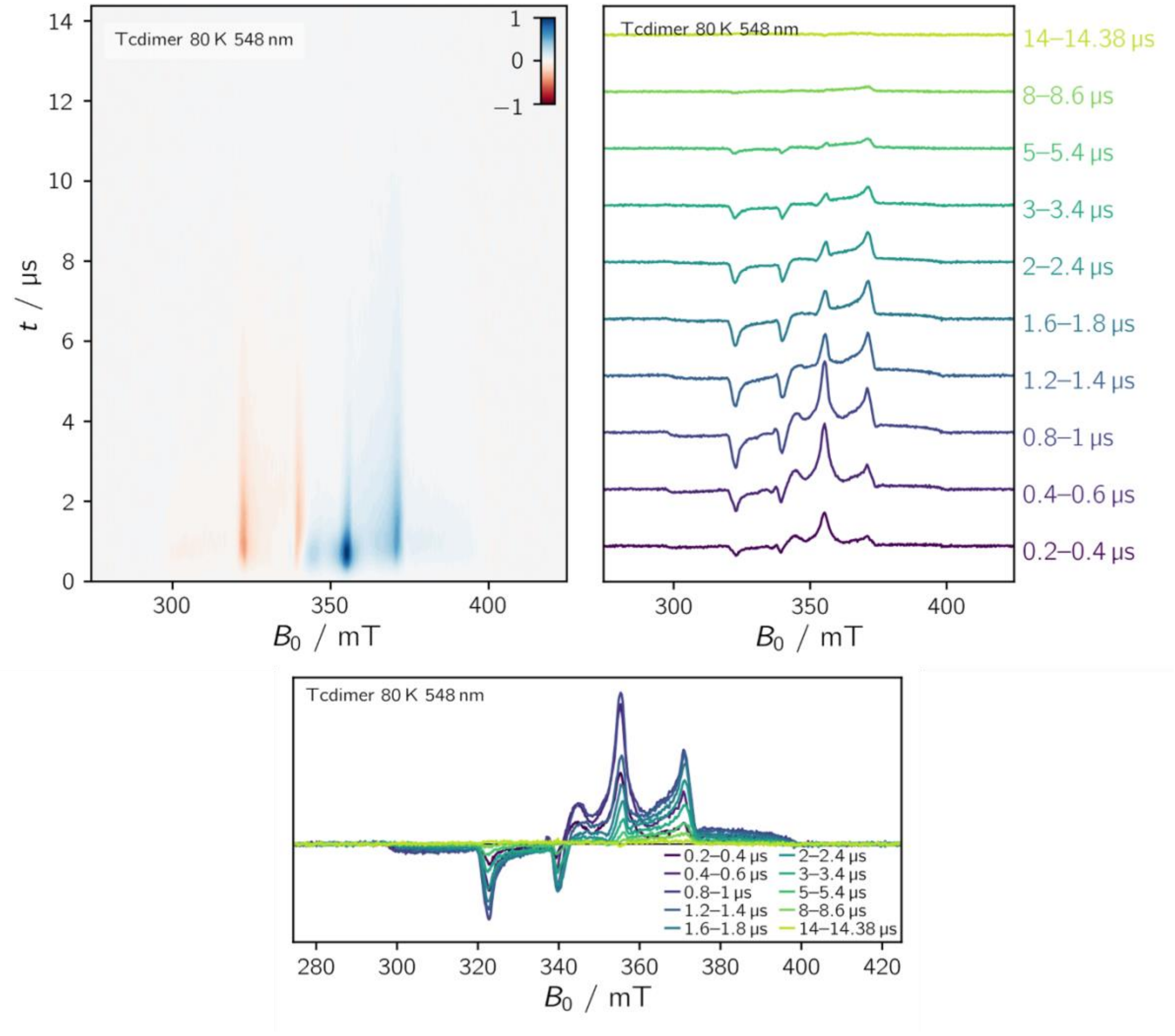

*Figure S56: Time-dependent evolution of the tr-EPR spectra of **Tcdimer** recorded at 80 K.*

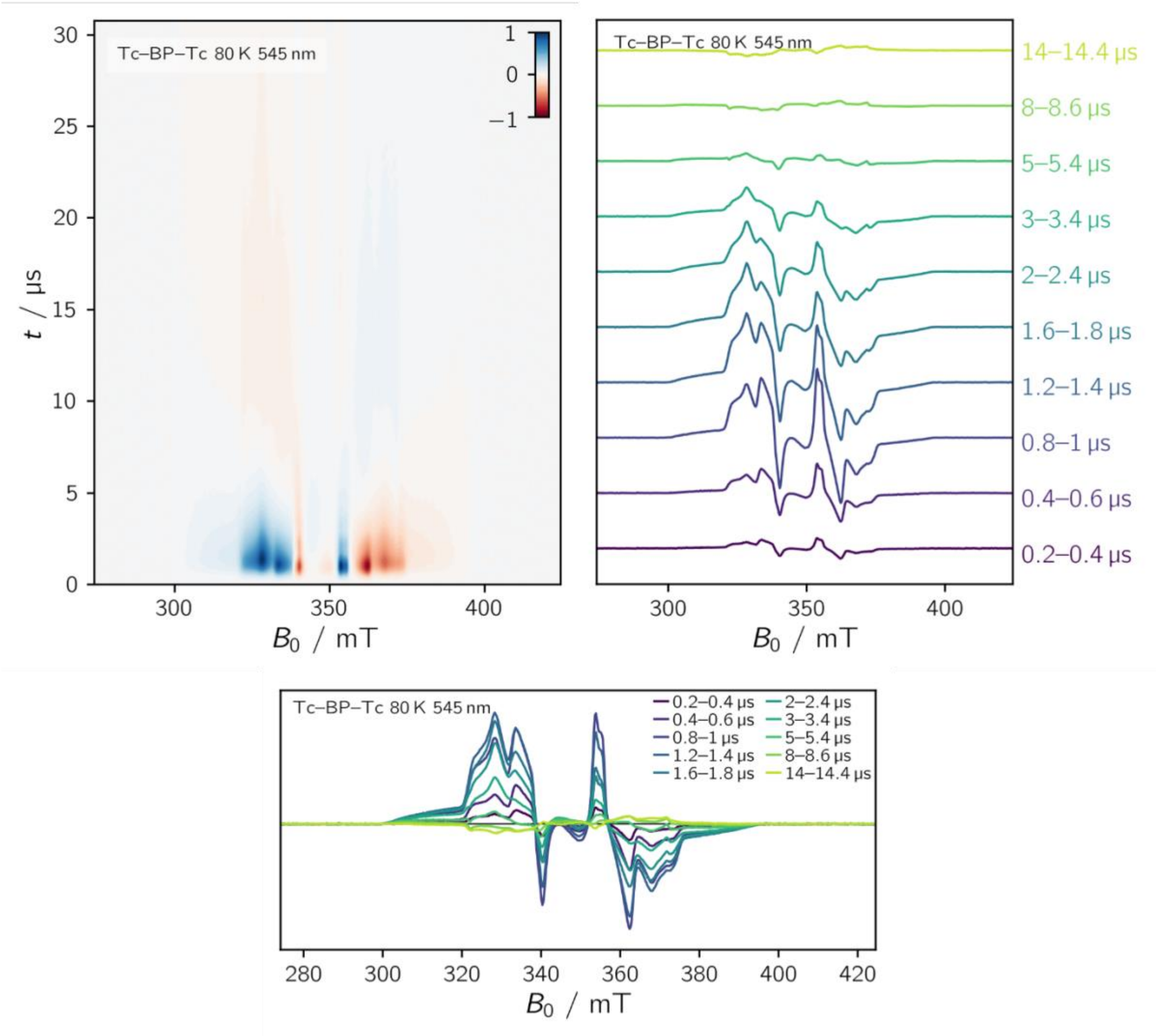

*Figure S57: Time-dependent evolution of the tr-EPR spectra of **Tc-BP-Tc** recorded at 80 K.*

Pulsed EPR data:

Field-dependent transient nutation experiments were performed at the Q-band for Tcdimer and are presented in Figure S58. Assuming S and M_S are good quantum numbers and the mw radiation excites a single transition, the observed nutation frequencies follow

$$\omega_{nut}(m_s, m_{S+1}) = \omega_1\sqrt{S(S+1) - m_S(m_S+1)}$$

where $\omega_1 = g_1\mu_B B_1/\hbar$ If the $\overrightarrow{B_1}$ field strength can be determined from a reference measurement and the g-factors are known, the S and M_S quantum numbers can be determined from the nutation frequency. The field-positions chosen for the transient nutation experiments are indicated in Figure S56(a) alongside the two-pulse echo-detected field sweep and the time- and frequency-domain transient nutation data are presented in Figure S56(b,c). The transient nutation data indicate two clear nutation frequencies in agreement with the $\sqrt{2\omega_1}$ and $\sqrt{6\omega_1}$ frequencies, characteristic of the $|1, \pm1\rangle \leftrightarrow |1, 0\rangle$ and $|2, \pm1\rangle \leftrightarrow |2, 0\rangle$ transitions, providing evidence for

the existence of strongly coupled triplet pairs and free triplets at different field positions, consistent with the discussion and assignment of the trEPR spectrum in the main text.

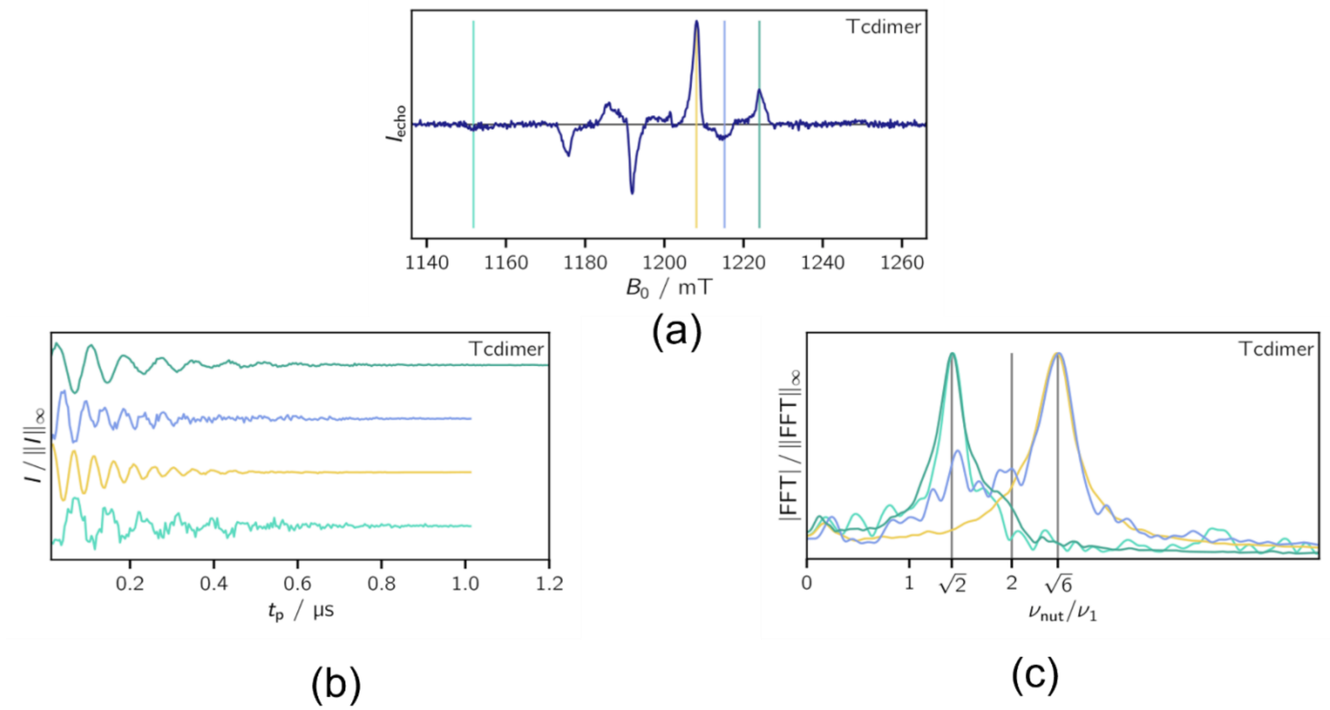

Figure S58: (a) Echo-dected fieldsweep for Tcdimer recorded at the Q-band, 80 K with a 700 ns delay following the 548 nm LASER pulse. The field positions used in transient nutation experiments are indicated by the coloured vertical lines. Transient nutation data as a function of the length of the first microwave pulse tp (b) and the corresponding Fourier transforms (c) for Tcdimer recorded in frozen toluene solution at 80 K. The frequency abssica is normalised against a reference nutation frequency measured for a spin-1/2. Frequencies relevant for triplet and quintet states are indicated with the grey vertical lines.

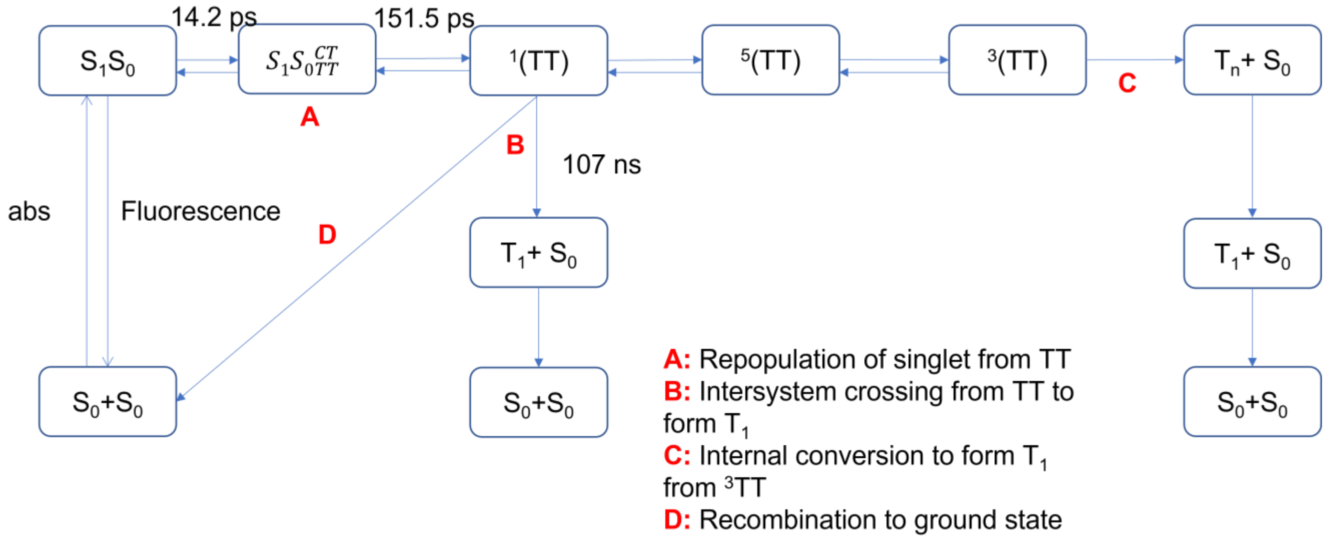

Scheme 1: Scheme showing possible pathways occurring in Tcdimer upon photoexcitation

14. Computational details

14.1 Absorption of Tcdimer, TIPS-Tc and linker

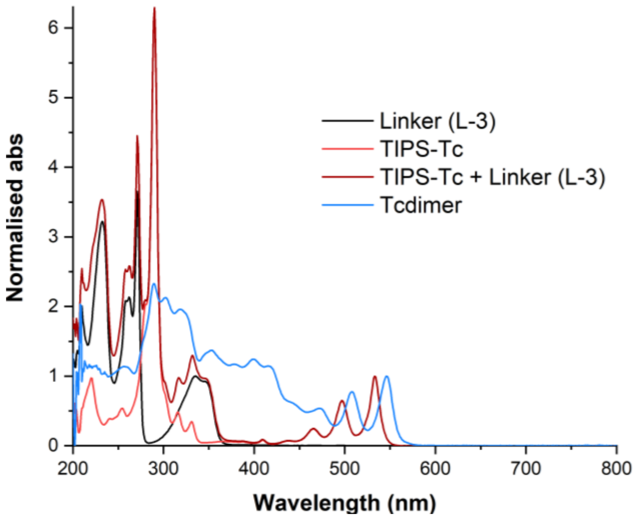

Figure S59: Experimental UV-Visible spectra of the linker, TIPS-Tc and Tcdimer in THF.

Absorption features of **Tcdimer**, in particular that observed experimentally in the 380-420 nm region, were explored computationally considering individual relevant fragments. All geometries were optimized at the CAM-B3LYP/6-31G(d) level of theory and vertical excitations were obtained using TDADFT at the same level.

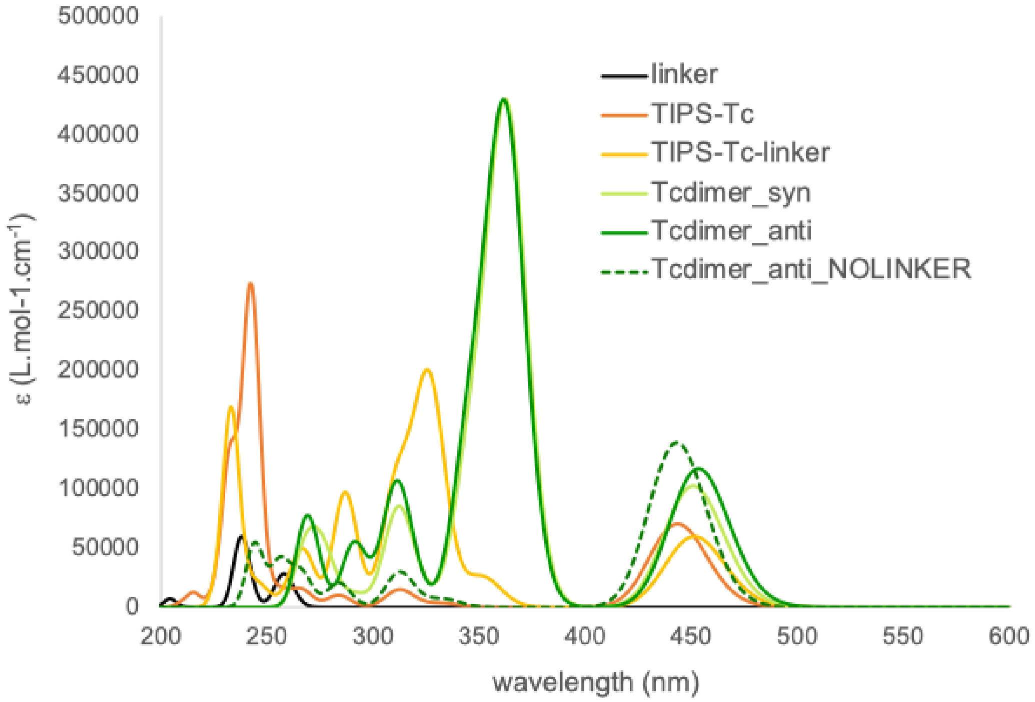

Figure S60. Simulated UV-Vis spectra of the linker (black line), TIPS-Tc (orange), TIPS-Tc-linker (yellow), Tcdimer-syn (light green), Tcdimer-anti (green) and Tcdimer-anti without the linker (dashed dark green).

The low energy band corresponds to excitations on the tetracene unit of TIPS-Tc and TIPS-Tc-linker, and to the bright state resulting from the coupling between the excitations localized on the Tc units of the Tcdimer with and without linker (Figure S61). The absorption spectrum of the Tcdimer without the linker closely matches that of TIPS-Tc, with a distribution of states corresponding to that of the excitonic states arising from its S_1-S_3 states (Table S15).

Addition of the linker to TIPS-Tc leads to the emergence of additional states with mixed local and charge transfer (CT) character. In particular, S_2 and S_3 are dominantly CT. The band computed at about 360 nm for the Tcdimer arises from the coupling of these excitations involving the Tc units and the linker; accordingly, they do not appear when considering the Tcdimer without linker.

Table S15: Excitation energy (in eV) and associated oscillator strength for the low-lying singlet states of the fragments computed at their respective ground state geometries at the CAM-B3LYP/6-31G(d) level.

	TIPS-Tc	TIPS-Tc-linker	Tcdimer anti	Tcdimer anti no linker
S_1	2.795 (0.523)	2.746 (0.445)	2.734 (0.870)	2.793 (0.000)
S_2	3.726 (0.025)	3.525 (0.190)	2.747 (0.000)	2.797 (1.033)
S_3	3.966 (0.112)	3.797 (1.411)	3.419 (3.001)	3.720 (0.052)
S_4		3.984 (0.779)	3.493 (0.000)	3.720 (0.000)
S_5			3.584 (1.087)	3.964 (0.016)
S_6			3.853 (0.000)	3.964 (0.207)
S_7			3.922 (0.000)	
S_8			3.929 (0.210)	

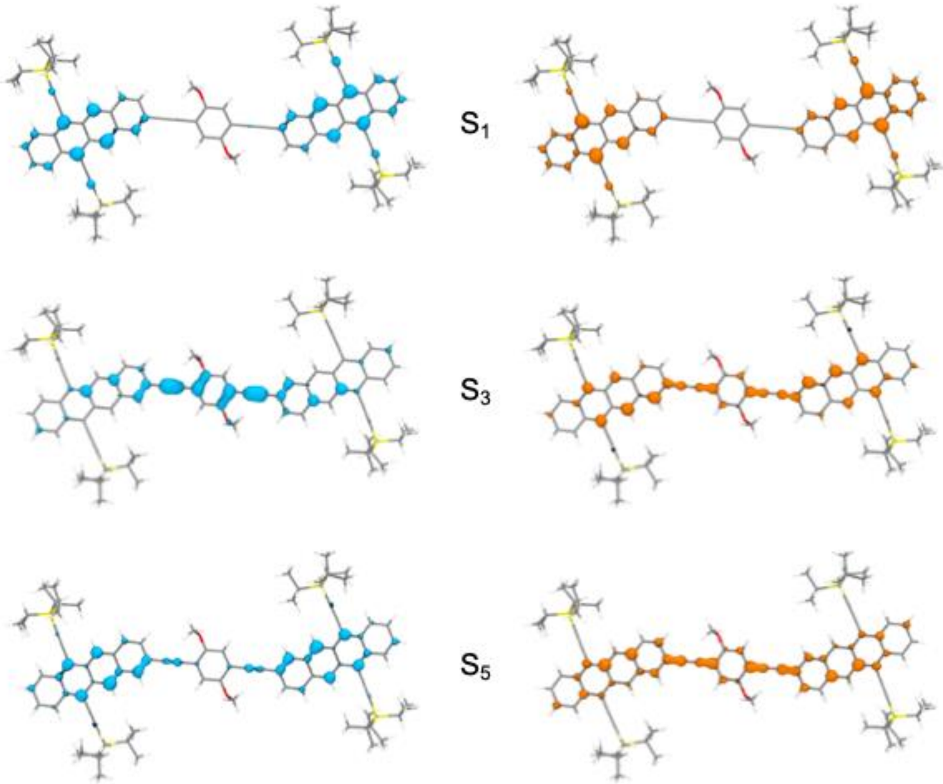

Figure S61. Electron/hole pair densities (orange/blue) for the S_1, S_3 and S_5 states of Tcdimer-anti as obtained at its ground state geometry at the CAM-B3LYP/6-31G(d) level in gas phase.

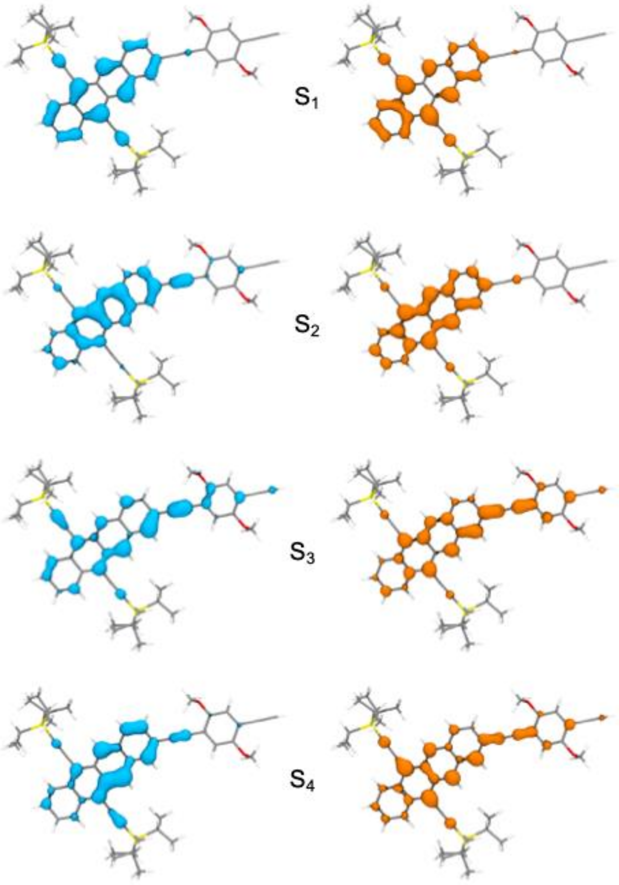

Figure S62. Electron/hole pair densities (orange/blue) for the S_1-S_4 states of TIPC-Tc-linker as obtained at its ground state geometry at the CAM-B3LYP/6-31G(d) level in gas phase.

14.2 Tcdimer anti/syn conformers

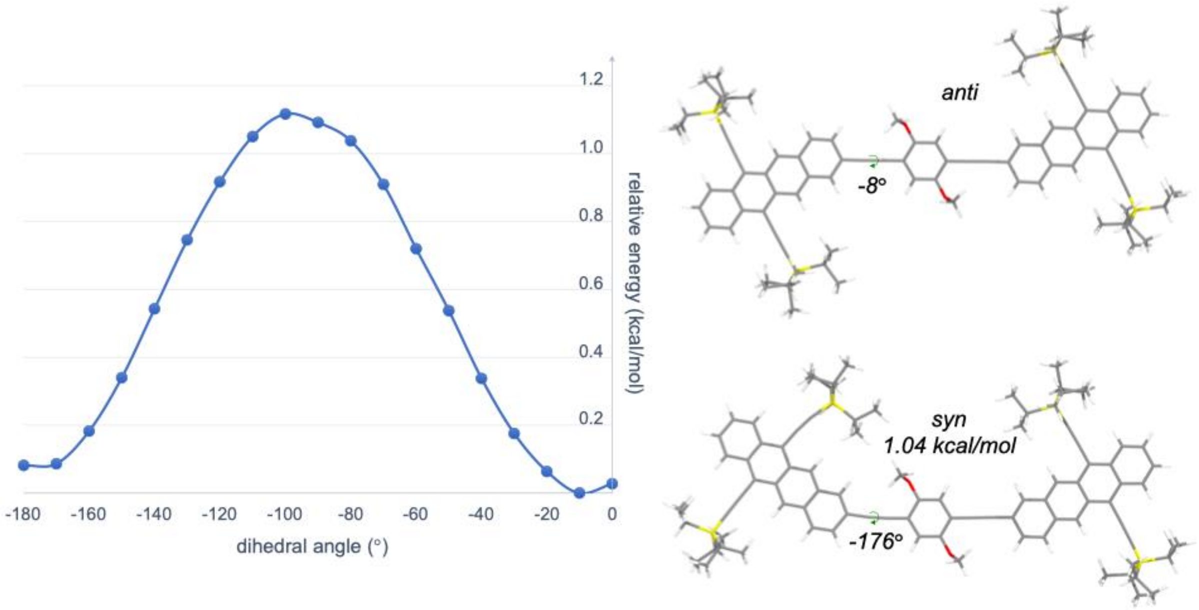

Figure S63. (left) Rigid scan around the acetylene bond and (right) geometry of the anti and syn conformers of the ***Tcdimer*** *with associated value of the dihedral angle and relative Gibbs free energy.*

The *syn* conformer is found to be only slightly less stable than the *anti* counterpart, and accessible via a low rotational barrier (Figure S63).

14.3 Photophysical properties of anticonformers of Tcdimer with chloranil and TCAQ

Ground and quintet states geometries of **Tcdimer-Chl**, **Tc-dimer-TCAQ** and **Tc-BP-Tc** without and with chloranil, were optimized using density functional theory (DFT) at the CAM-B3LYP/6-31G(d) level. Several starting geometries were considered to explore the conformational space of the dimers. CT state geometries were optimized within the framework of time-dependent DFT at the same level of theory. On the basis of the lowest energy structure obtained for each dimer, vertical excitations were computed at the same level of theory within the framework of time-dependent DFT with the Tamm-Dancoff approximation (TDADFT). All calculations were done with the Gaussian 16 program package.[19] The DrawMol program was used for visualisation of the molecular orbitals.[20]

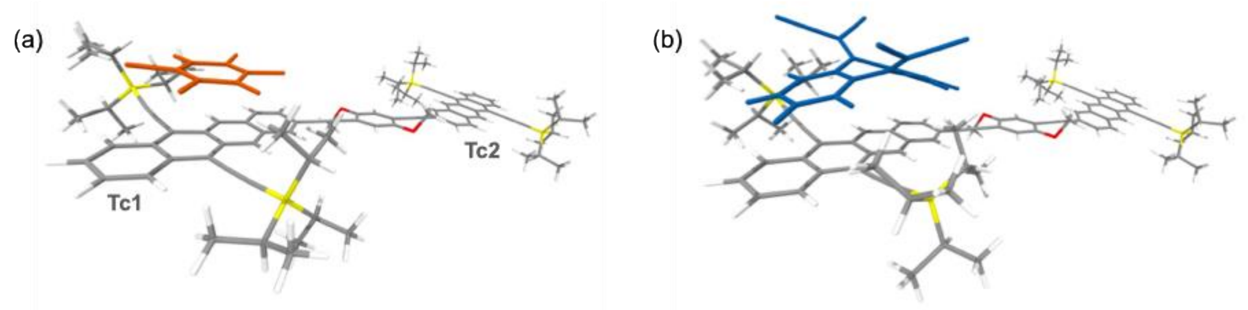

Figure S64: Geometry of the lowest energy dimer of (a) ***Tcdimer-Chl*** *and (b)* ***Tcdimer-TCAQ****.*

Table S16*: Excitation energy (in eV) and contributions (>5%) for the low-lying triplet states of* ***Tcdimer-Chl*** *computed at the ground state and triplet-pair (quintet) optimized geometries.*

state	ΔE	contrib. %	
GS geometry			
^{3}LE2 (Tc2)	1.299	H→L+2	72
		H→L+1	8
		H-1→L+2	8
^{3}LE1 (Tc1)	1.328	H-1→L+1	72
		H→L+1	6
		H-1→L+2	5
^{3}CT	1.442	H-1→L	84
		H→L	9
TT (LE1+LE2)	2.627		
quintet geometry			
^{3}LE2 (Tc2)	0.693	H→L+2	83
		H→L+1	5
^{3}LE1 (Tc1)	0.736	H-1→L+1	82
^{3}CT	1.168	H-1→L	92
		H→L	5
TT (LE1+LE2)	1.429		
CT geometry			
^{3}CT	0.594	H-1→L	16

		H→L	81
^{3}LE1 (Tc1)	1.062	H-1→L+1	15
		H→L+1	71
^{3}LE2 (Tc2)	1.300	H-1→L+2	69
		H→L+2	15

***Table S17**: Excitation energy (in eV) and contributions for the low-lying triplet states of **Tcdimer-TCAQ** computed at the ground state and triplet-pair (quintet) optimized geometries.*

state	ΔE	contrib. %	
GS geometry			
^{3}LE1 (Tc1)	1.288	H-1→L+1	80
^{3}LE2 (Tc2)	1.299	H→L+2	82
^{3}CT	2.341	H-1→L	49
		H-2→L+1	6
TT (LE1+LE2)	2.587		
quintet geometry			
^{3}LE1 (Tc1)	0.666	H-1→L	23
		H-1→L+1	67
^{3}LE2 (Tc2)	0.693	H→L+2	90
^{3}CT	2.099	H-1→L	69
		H-1→L+1	24
TT (LE1+LE2)	1.359		
CT geometry*			
^{3}LE1' (Tc1)	1.013	H-1→L	10
		H-1→L+1	31
		H→L	13
		H→L+1	37
^{3}CT	1.107	H-1→L	32
		H-1→L+1	9
		H→L	42
		H→L+1	11
^{3}LE2' (Tc2)	1.299	H-1→L+2	46
		H→L+2	39

*here, the two LE states nomenclature is maintained for ease of comparison throughout the different geometries considered, a prime mark is used to denote their more mixed nature

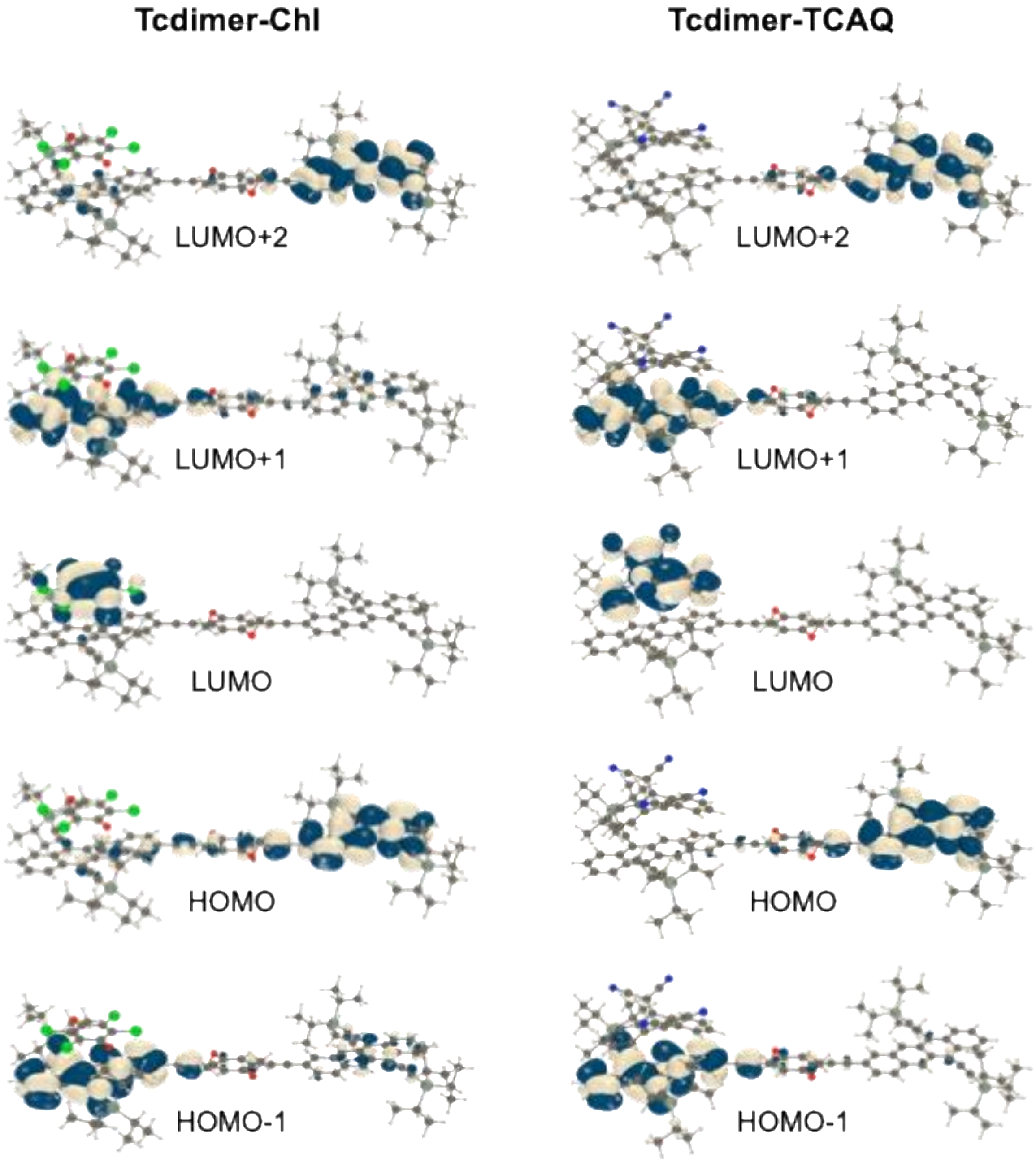

Figure S65: Molecular orbitals of (left) ***Tcdimer-Chl*** *and (right)* ***Tcdimer-TCAQ*** *at their respective ground state geometry.*

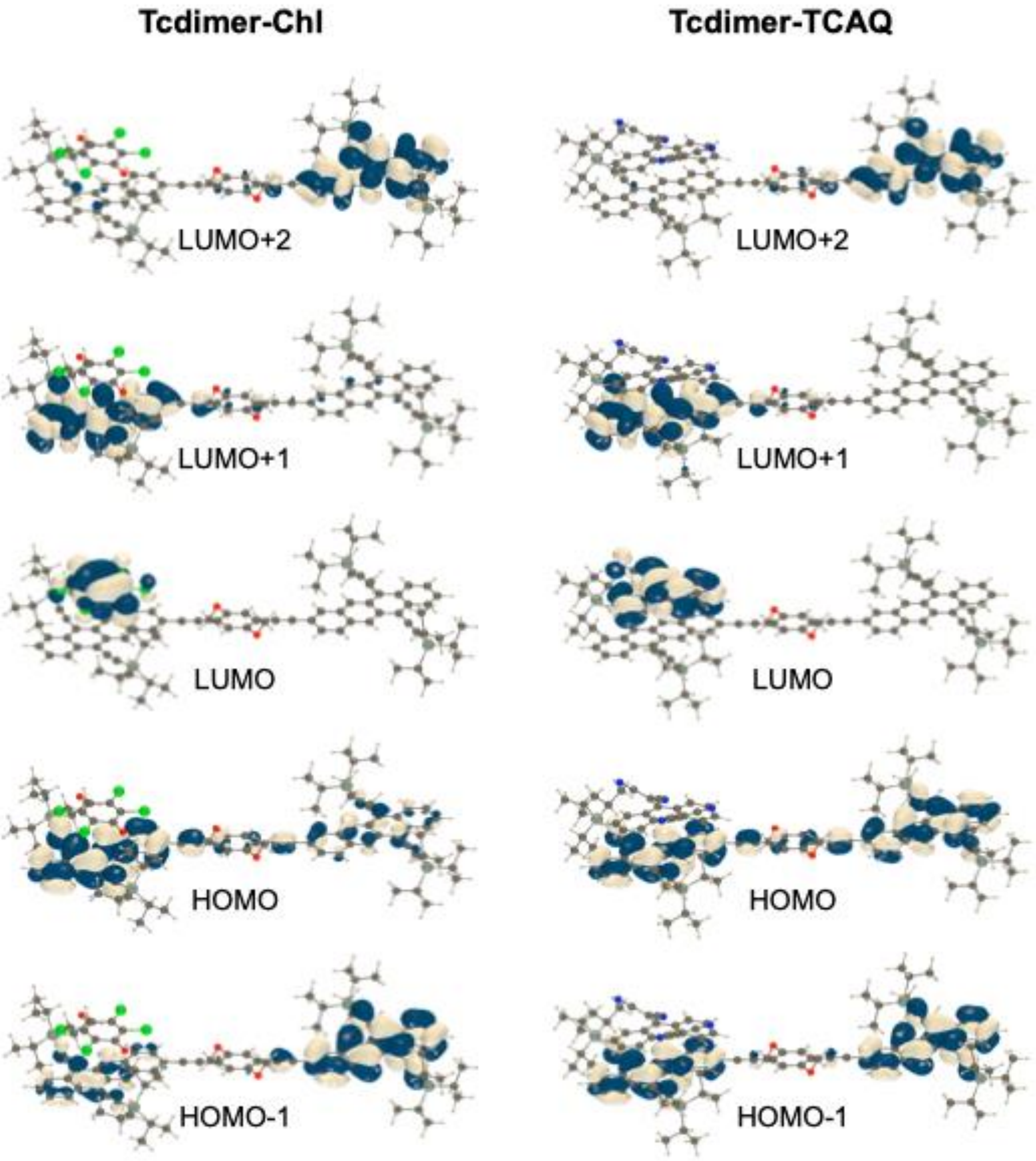

Figure S66: Molecular orbitals of (left) ***Tcdimer-Chl*** *and (right)* ***Tcdimer-TCAQ*** *at their respective CT state geometry.*

14.4 Photophysical properties of *syn* conformers with chloranil

To address the possible effect of conformational heterogeneity, we explored the ***syn01*-Chl** and ***syn02*-Chl** Tcdimers, where the chloranil molecule sits on one or the other of the Tc units. The two optimized dimers and the ***anti*-Chl** (**Tcdimer-Chl**) one show relative Gibbs free energies within 1 kcal/mol, in the following order: *syn02*-Chl < *anti*-Chl < *syn01*-Chl). On the basis of the two *syn* structures obtained, we computed vertical excitations at the CAM-B3LYP/6-31G(d) level of theory within the framework of time-dependent DFT with the Tamm-Dancoff approximation (TDADFT).

*Table S18: Excitation energy (in eV) and contributions (>5%) for the low-lying triplet states of **syn01-Chl** computed at the ground state and triplet-pair (quintet) optimized geometries.*

state		ΔE	contrib. %	
GS geometry				
^{3}LE2	(Tc2)	1.297	H→L+2	72
	(Tc1→Tc2)		H-1→L+2	9
	(Tc2→Tc1)		H→L+1	8
^{3}LE1	(Tc1)	1.327	H-1→L+1	71
	(Tc2→Tc1)		H→L+1	6
	(Tc1→Tc2)		H-1→L+2	5
^{3}CT	(Tc1→Chl)	1.433	H-1→L	83
	(Tc2→Chl)		H→L	9
TT	(LE1+LE2)	2.624		
quintet geometry				
^{3}LE2	(Tc2)	0.689	H→L+2	86
^{3}LE1	(Tc1)	0.738	H-1→L+1	85
^{3}CT	(Tc1→Chl)	1.161	H-1→L	93
TT	(LE1+LE2)	1.427		

*Table S19: Excitation energy (in eV) and contributions (>5%) for the low-lying triplet states of **syn02-Chl** computed at the ground state and triplet-pair (quintet) optimized geometries.*

state		ΔE	contrib. %	
GS geometry				
^{3}LE1	(Tc1)	1.298	H→L+2	73
	(Tc2→Tc1)		H-1→L+2	9
	(Tc1→Tc2)		H→L+1	7
^{3}LE2	(Tc2)	1.325	H-1→L+1	75
	(Tc1→Tc2)		H→L+1	6
^{3}CT	(Tc2→Chl)	1.416	H-1→L	87
	(Tc1→Chl)		H→L	9
TT	(LE1+LE2)	2.623		
quintet geometry				
^{3}LE1	(Tc1)	0.693	H→L+2	84
^{3}LE2	(Tc2)	0.737	H-1→L+1	85
^{3}CT	(Tc2→Chl)	1.145	H-1→L	93
TT	(LE1+LE2)	1.430		

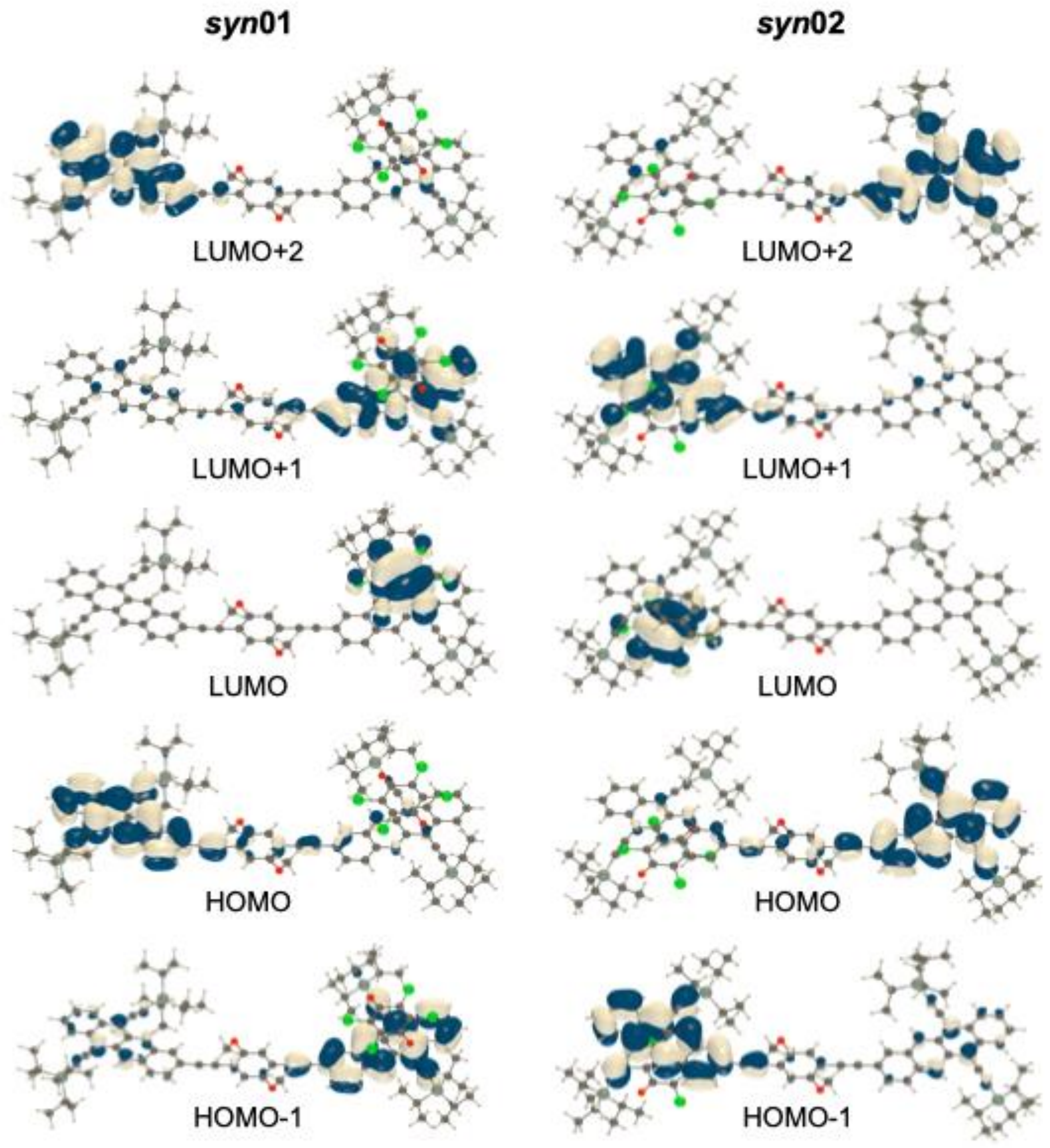

Figure S67. Molecular orbitals of (left) ***syn01-Chl*** *and (right)* ***syn02-Chl*** *at their respective ground state geometry.*

14.5 Photophysical properties of *Tc-BP-Tc and Tc-BP-Tc-Chl*

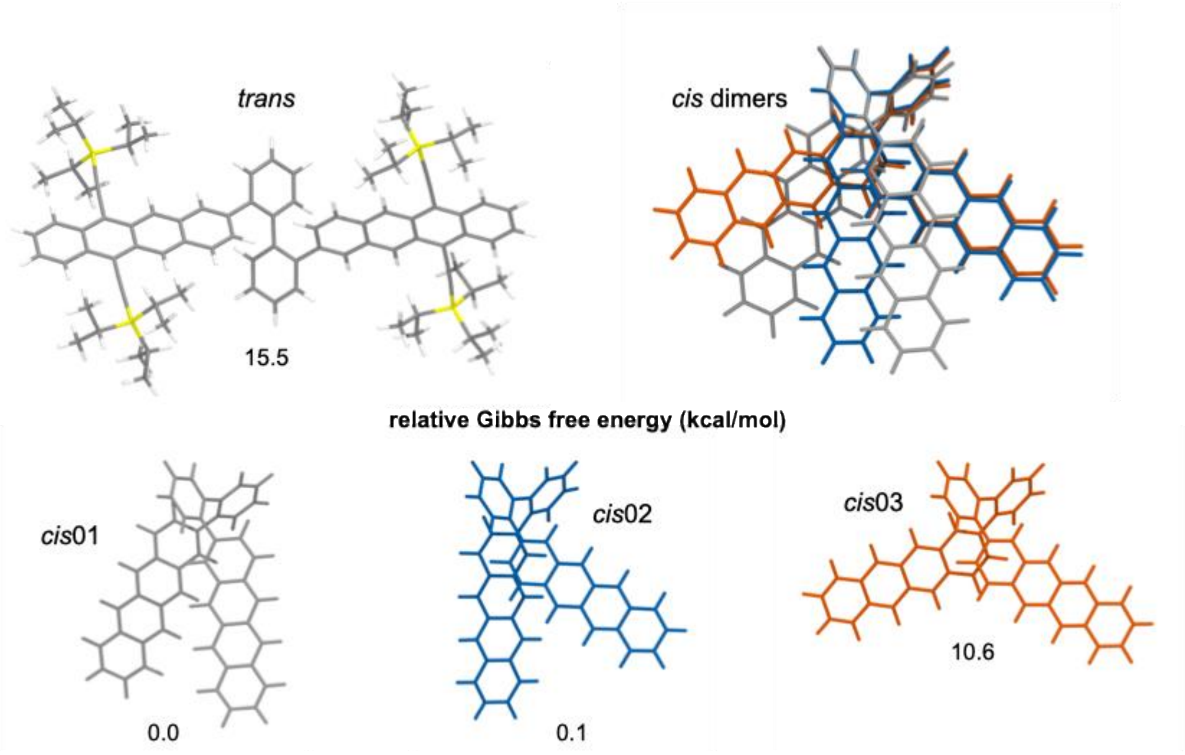

Figure S68:. Geometry of the stable trans and cis conformers of the ***Tc-BP-Tc****.*

Table S20*: Structural parameters of the cis dimers: α angle between the Tc units long axis, θ_1 biphenyl dihedral angle*

	α	θ_1
cis01	24	62
cis02	61	60
cis03	129	59

We considered both trans and cis type geometries as starting point for exploring the conformational space of **Tc-BP-Tc**. All cis dimers are found to be more stable than the trans counterpart, with notably two conformers being almost isoenergetic. These structures differ in the relative orientation of the tetracene moieties which translate into varying extent of stacking of the Tc units. In the following, we focus on the two most stable conformers, cis01 and cis02.

Table S21*: Excitation energy (in eV) and contributions (>5%) for the low-lying triplet states of* ***cis01-Chl*** *computed at the ground state and triplet-pair (quintet) optimized geometries.*

state		ΔE	contrib.	%
	GS geometry			
T_1	(Tc1→Chl)	1.304	H-1→L	15
	LE(Tc1)		H-1→L+1	26
	(Tc1→Tc2)		H-1→L+2	10
	(Tc2→Tc1)		H→L+1	14
	LE(Tc2)		H→L+2	23
T_2	(Tc1→Chl)	1.307	H-1→L	10
	LE(Tc1)		H-1→L+1	22
	(Tc1→Tc2)		H-1→L+2	5
	LE(Tc2)		H→L+2	52
T_3	(Tc1→Chl)	1.392	H-1→L	51
	LE(Tc1)		H-1→L+1	24
	(Tc2→Chl)		H→L	15

(Tc2→Tc1)		H→L+1	6
T4 (Tc1→Chl)	2.307	H-1→L	21
(Tc2→Chl)		H→L	77
TT (LE1+LE2)	2.611		
quintet geometry			
^{3}LE2 (Tc2)	0.690	H→L+2	77
		H-1→L+2	12
		H→L+1	5
^{3}LE1 (Tc1)	0.727	H-1→L+1	69
		H→L+1	13
		H-1→L	7
^{3}CT1 (Tc1→Chl)	1.094	H-1→L	74
		H→L	17
		H-1→L+1	6
^{3}CT2 (Tc2→Chl)	1.997	H→L	79
		H-1→L	18
TT (LE1+LE2)	1.417		
CT geometry			
^{3}CT1 (Tc1→Chl)	0.497	H→L	82
		H-1→L	17
^{3}LE1 (Tc1)	1.061	H→L+1	73
		H-1→L+1	18
^{3}LE2 (Tc2)	1.304	H-1→L+2	67
		H→L+2	22
^{3}CT2 (Tc2→Chl)	1.593	H-1→L	80
		H→L	17

***Table S22**: Excitation energy (in eV) and contributions (>5%) for the low-lying triplet states of **cis02-Chl** computed at the ground state and triplet-pair (quintet) optimized geometries.*

state	ΔE	contrib. %	
GS geometry			
^{3}LE2 (Tc2)	1.285	H→L+2	82
		H→L+1	7
^{3}LE1 (Tc1)	1.337	H-1→L+1	78
		H-1→L+2	6
		H-1→L	5
^{3}CT1 (Tc1→Chl)	1.413	H-1→L	91
		H-1→L+1	5
^{3}CT2 (Tc2→Chl)	2.320	H→L	96
TT (LE1+LE2)	2.622		
quintet geometry			
^{3}LE2 (Tc2)	0.645	H→L+2	84
		H→L+1	9
^{3}LE1 (Tc1)	0.736	H-1→L+1	84
		H-1→L+2	8
^{3}CT1 (Tc1→Chl)	1.143	H-1→L	97
^{3}CT2 (Tc2→Chl)	2.037	H→L	98
TT (LE1+LE2)	1.381		
CT geometry			
^{3}CT1 (Tc1→Chl)	0.587	H→L	98
^{3}LE1 (Tc1)	1.071	H→L+1	90
^{3}LE2 (Tc2)	1.288	H-1→L+2	89
^{3}CT2 (Tc2→Chl)	1.676	H-1→L	99

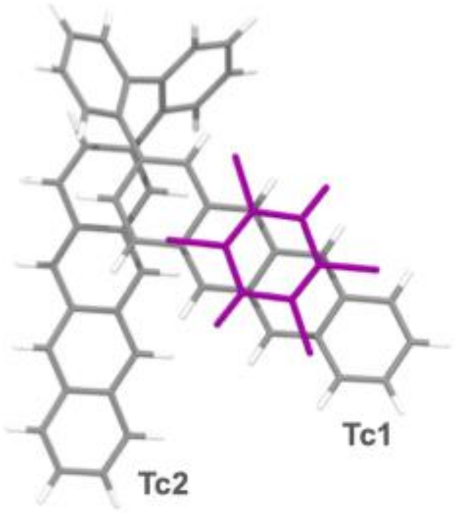

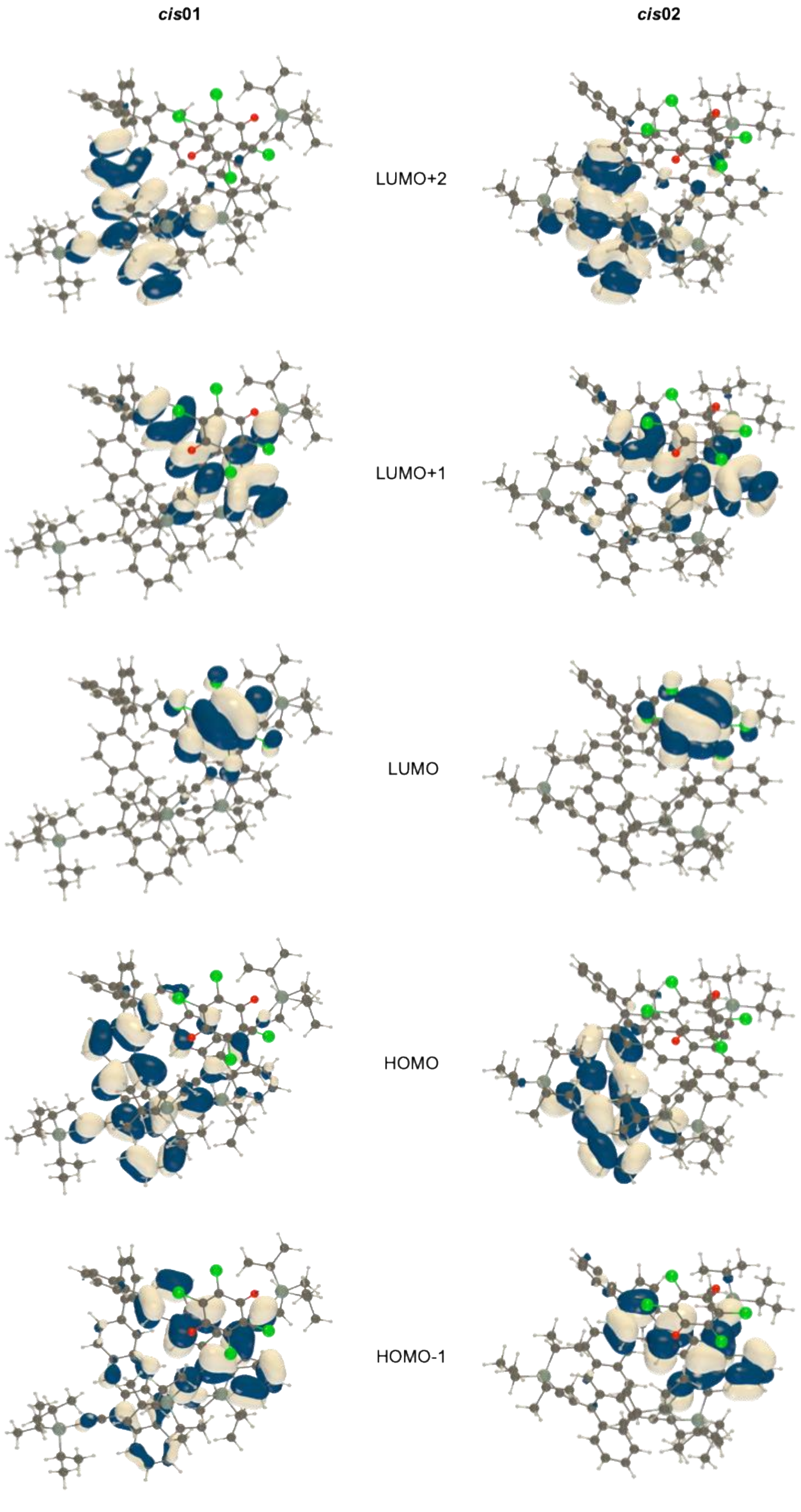

Figure S69. Molecular orbitals of (left) ***cis01-Chl*** *and (right)* ***cis02-Chl*** *at their respective ground state geometry.*

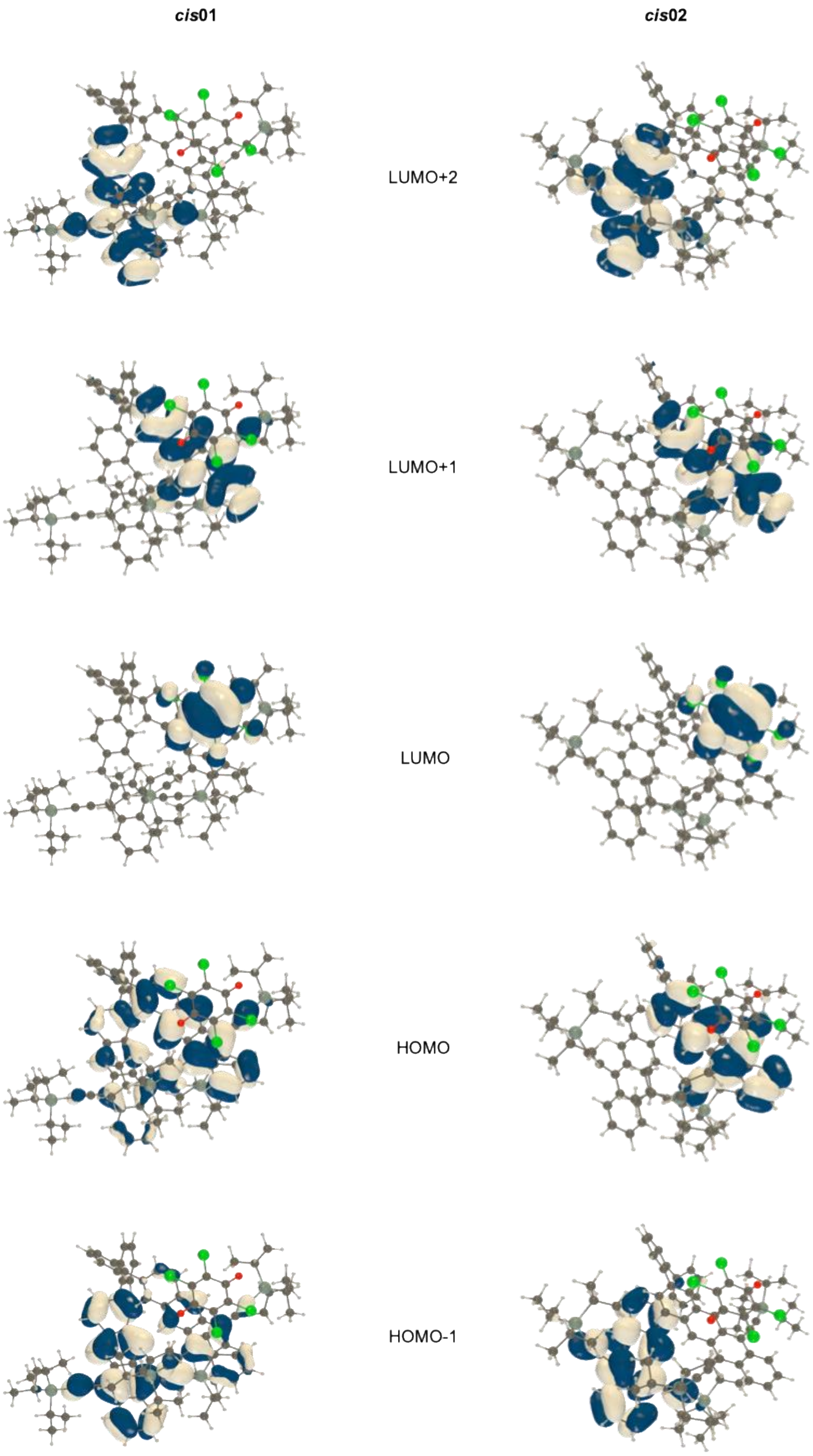

Figure S70. Molecular orbitals of (left) ***cis01-Chl*** *and (right)* ***cis02-Chl*** *at their respective CT state geometry.*

14.6 Modelling the second electron transfer in Tcdimer-Chl and Tc-BP-Tc-Chl

The second electron transfer is expected to be virtually unaffected by the first CT at the other tetracene unit. To address this, the **Tcdimer-Chl** system in its cation state is considered as the starting point for the second electron transfer, where one tetracene unit is already oxidized (^{3}Tc-Tc$^+$). At this optimized geometry, where the positive charge is localized over the non-interacting tetracene unit, the CT state energy for **Tcdimer-Chl** cation without and with a negative point charge placed next to the oxidized tetracene amounts to 1.59 eV and 1.53 eV, respectively, values comparable to that of the first electron transfer (1.44 eV, see Table S16). At the optimized geometry of ***cis*01-Chl** and ***cis*02-Chl** in their cation state, the CT state energy (without placing a negative point charge next to the oxidized tetracene unit) amounts to 1.65 eV and 1.67 eV, respectively.

15 References

(1) Nakamura, S.; Sakai, H.; Nagashima, H.; Kobori, Y.; Tkachenko, N. V.; Hasobe, T. Quantitative Sequential Photoenergy Conversion Process from Singlet Fission to Intermolecular Two-Electron Transfers Utilizing Tetracene Dimer. *ACS Energy Lett.* **2019**, *4*, 26–31. https://doi.org/10.1021/acsenergylett.8b01964.

(2) Kubin, R. F.; Fletcher, A. N. Fluorescence Quantum Yields of Some Rhodamine Dyes. *J. Lumin.* **1982**, *27*, 455–462. https://doi.org/10.1016/0022-2313(82)90045-X.

(3) Herb, K.; Tschaggelar, R.; Denninger, G.; Jeschke, G. Double Resonance Calibration of g Factor Standards: Carbon Fibers as a High Precision Standard. *J. Magn. Reson.* **2018**, *289*, 100–106. https://doi.org/10.1016/j.jmr.2018.02.006.

(4) Tait, C. E.; Krzyaniak, M. D.; Stoll, S. Computational Tools for the Simulation and Analysis of Spin-Polarized EPR Spectra. *J. Magn. Reson.* **2023**, *349*, 107410. https://doi.org/10.1016/j.jmr.2023.107410.

(5) Stoll, S.; Schweiger, A. EasySpin, a Comprehensive Software Package for Spectral Simulation and Analysis in EPR. *J. Magn. Reson.* **2006**, *178* (1), 42–55. https://doi.org/10.1016/j.jmr.2005.08.013.

(6) Sanders, S. N.; Kumarasamy, E.; Pun, A. B.; Steigerwald, M. L.; Sfeir, M. Y.; Campos, L. M.; Sanders, S. N.; Kumarasamy, E.; Pun, A. B.; Steigerwald, M. L.; Sfeir, M. Y.; Campos, L. M. Intramolecular Singlet Fission in Oligoacene Heterodimers. *Angew. Chem. - Int. Ed.* **2016**, *128*, 3434–3438. https://doi.org/10.1002/ange.201510632.

(7) Shen, K.; Qin, L.; Zheng, H.-G. Diverse Structures of Metal–Organic Frameworks via a Side Chain Adjustment: Interpenetration and Gas Adsorption. *Dalton Trans.* **2016**, *45* (41), 16205–16210. https://doi.org/10.1039/C6DT03086F.

(8) Rapp, M. R.; Weiß, R.; Wollny, A.-S.; Guldi, D. M.; Bettinger, H. F. Tuning Interactions to Control Molecular Down Conversion in [2.2]Paracyclophane Bridged Oligo-Tetracenes. *Adv. Funct. Mater.* **2024**, *34* (47), 2313576. https://doi.org/10.1002/adfm.202313576.

(9) Margulies, E. A.; Miller, C. E.; Wu, Y.; Ma, L.; Schatz, G. C.; Young, R. M.; Wasielewski, M. R. Enabling Singlet Fission by Controlling Intramolecular Charge Transfer in π-Stacked Covalent Terrylenediimide Dimers. *Nat. Chem.* **2016**, *8* (12). https://doi.org/10.1038/nchem.2589.

(10) Arshad, A.; Castellano, F. N. Homomolecular Triplet–Triplet Annihilation in Metalloporphyrin Photosensitizers. *J. Phys. Chem. A* **2024**, *128* (36), 7648–7656. https://doi.org/10.1021/acs.jpca.4c05052.

(11) Stern, H. L.; Musser, A. J.; Gelinas, S.; Parkinson, P.; Herz, L. M.; Bruzek, M. J.; Anthony, J.; Friend, R. H.; Walker, B. J. Identification of a Triplet Pair Intermediate in Singlet Exciton Fission in Solution. *Proc. Natl. Acad. Sci.* **2015**, *112* (25), 7656–7661. https://doi.org/10.1073/pnas.1503471112.

(12) Gray, V.; Allardice, J. R.; Zhang, Z.; Dowland, S.; Xiao, J.; Petty, A. J.; Anthony, J. E.; Greenham, N. C.; Rao, A. Direct vs Delayed Triplet Energy Transfer from Organic Semiconductors to Quantum Dots and Implications for Luminescent Harvesting of Triplet Excitons. *ACS Nano* **2020**, *14* (4), 4224–4234. https://doi.org/10.1021/acsnano.9b09339.

(13) Romero, N. A.; Nicewicz, D. A. Organic Photoredox Catalysis. *Chem. Rev.* **2016**, *116* (17), 10075–10166. https://doi.org/10.1021/acs.chemrev.6b00057.

(14) Bryden, M. A.; Millward, F.; Lee, O. S.; Cork, L.; Gather, M. C.; Steffen, A.; Zysman-Colman, E. Lessons Learnt in Photocatalysis – the Influence of Solvent Polarity and the Photostability of the Photocatalyst. *Chem. Sci.* **2024**, *15* (10), 3741–3757. https://doi.org/10.1039/D3SC06499A.

(15) Martín, N. Tetrathiafulvalene: The Advent of Organic Metals. *Chem. Commun.* **2013**, *49* (63), 7025–7027. https://doi.org/10.1039/C3CC00240C.

(16) Kivala, M.; Boudon, C.; Gisselbrecht, J.-P.; Enko, B.; Seiler, P.; Müller, I. B.; Langer, N.; Jarowski, P. D.; Gescheidt, G.; Diederich, F. Organic Super-Acceptors with Efficient Intramolecular Charge-Transfer Interactions by [2+2] Cycloadditions of TCNE, TCNQ, and F4-TCNQ to Donor-Substituted Cyanoalkynes. *Chem. – Eur. J.* **2009**, *15* (16), 4111–4123. https://doi.org/10.1002/chem.200802563.

(17) Gschwind, R.; Haselbach, E. Laserflash-Photolysis of the p-Chloranil/Naphthalene System: Characterization of the Naphthalene Radical Cation in a Fluid Medium,. *Helv. Chim. Acta* **1979**, *62* (4), 941–955. https://doi.org/10.1002/hlca.19790620404.

(18) Kiefer, D.; Kroon, R.; Hofmann, A. I.; Sun, H.; Liu, X.; Giovannitti, A.; Stegerer, D.; Cano, A.; Hynynen, J.; Yu, L.; Zhang, Y.; Nai, D.; Harrelson, T. F.; Sommer, M.; Moulé, A. J.; Kemerink, M.; Marder, S. R.; McCulloch, I.; Fahlman, M.; Fabiano, S.; Müller, C. Double Doping of Conjugated Polymers with Monomer Molecular Dopants. *Nat. Mater.* **2019**, *18* (2), 149–155. https://doi.org/10.1038/s41563-018-0263-6.

(19) Frisch, M. J.; Trucks, G. W.; Schlegel, H. B.; Scuseria, G. E.; Robb, M. A.; Cheeseman, J. R.; Scalmani, G.; Barone, V.; Petersson, G. A.; Nakatsuji, H.; Li, X.; Caricato, M.; Marenich, A. V.; Bloino, J.; Janesko, B. G.; Gomperts, R.; Mennucci, B.; Hratchian, H. P.; Ortiz, J. V.; Izmaylov, A. F.; Sonnenberg, J. L.; Williams; Ding, F.; Lipparini, F.; Egidi, F.; Goings, J.; Peng, B.; Petrone, A.; Henderson, T.; Ranasinghe, D.; Zakrzewski, V. G.; Gao, J.; Rega, N.; Zheng, G.; Liang, W.; Hada, M.; Ehara, M.; Toyota, K.; Fukuda, R.; Hasegawa, J.; Ishida, M.; Nakajima, T.; Honda, Y.; Kitao, O.; Nakai, H.; Vreven, T.; Throssell, K.; Montgomery Jr., J. A.; Peralta, J. E.; Ogliaro, F.; Bearpark, M. J.; Heyd, J. J.; Brothers, E. N.; Kudin, K. N.; Staroverov, V. N.; Keith, T. A.; Kobayashi, R.; Normand, J.; Raghavachari, K.; Rendell, A. P.; Burant, J. C.; Iyengar, S. S.; Tomasi, J.; Cossi, M.; Millam, J. M.; Klene, M.; Adamo, C.; Cammi, R.; Ochterski, J. W.; Martin, R. L.; Morokuma, K.; Farkas, O.; Foresman, J. B.; Fox, D. J. Gaussian 16 Rev. C.01, 2016.

(20) Liegeois, V. DrawMol. www.unamur.be/drawmol.
